# Supplementary material for: A comparative approach for species delimitation based on multiple methods of multi-locus DNA sequence analysis: A case study of the genus Giraffa (Mammalia, Cetartiodactyla)
Source: PLoS One. 2020 Feb 13;15(2):e0217956. doi: 10.1371/journal.pone.0217956 (PMC7018015; doi:10.1371/journal.pone.0217956)
Supplement: S1 Appendix — (PDF) [file pone.0217956.s001.pdf]

S1 Appendix. Classifications of *Giraffa*

A comparative approach for species delimitation based on multiple methods of multi-locus DNA sequence analysis: a case study of the genus *Giraffa* (Mammalia, Cetartiodactyla)

Alice Petzold[1,2], Alexandre Hassanin[1,2]*

1 Institut de Systématique, Évolution, Biodiversité (ISYEB), Sorbonne Université, MNHN, CNRS, EPHE, Paris, France

2 Muséum national d'Histoire naturelle, CP51, 55 rue Buffon - 75005 Paris, France

*Correspondence: alexandre.hassanin@mnhn.fr

Table A. Taxonomic classifications proposed for *Giraffa camelopardalis*

Year	Author	Species	Subspecies (Synonyms)
**1758**	Linnaeus	*Cervus camelopardalis*	-
**1762**	Brisson	*Giraffa camelopardalis*	-
**1827**	Saint-Hilaire	**(1)** *"Girafe du nord"*<br>**(2)** *"Girafe du Cap"*	-
**1899**	De Winton	*Giraffa camelopardalis*	*G. c. camelopardalis*<br>*G. c. reticulata*
**1901**	Thomas	**(1)** *Giraffa camelopardalis*<br><br>**(2)** *Giraffa reticulata*	**(1)** *G. c. camelopardalis*<br>*G. c. peralta*<br>**(2)** *G. r. reticulata*
**1914**	Lydekker	**(1)** *Giraffa camelopardalis*<br><br><br><br><br><br><br><br><br><br><br><br>**(2)** *Giraffa reticulata*	**(1)** *G. c. camelopardalis*<br>*G. c. angolensis*<br>*G. c. antiquorum*<br>*G. c. capensis*<br>*G. c. congoensis*<br>*G. c. cottoni*<br>*G. c. infumata*<br>*G. c. peralta*<br>*G. c. rothschildi*<br>*G. c. thornicrofti*<br>*G. c. tippelskirchi*<br>*G. c. wardi*<br>**(2)** *G. r. reticulata*<br>*G. r. nigrescens*
**1971**	Krumbiegel	**(1)** *Giraffa camelopardalis*<br><br><br><br><br><br><br>**(2)** *Giraffa capensis*	**(1)** *G. c. camelopardalis*<br>*G. c. antiquorum*<br>*G. c. congoensis*<br>*G. c. cottoni*<br>*G. c. peralta*<br>*G. c. reticulata*<br>*G. c. rothschildi*<br>**(2)** *G. c. capensis*<br>*G. c. angolensis*<br>*G. c. infumata*<br>*G. c. tippelskirchi*<br>*G. c. thornicrofti*<br>*G. c. wardi*

**1976**	Dagg and Foster	*Giraffa camelopardalis*	*G. c. camelopardalis*<br>*G. c. angolensis*<br>*G. c. antiquorum*<br>*G. c. giraffa*<br>*G. c. peralta*<br>*G. c. reticulata*<br>*G. c. rothschildi*<br>*G. c. thornicrofti*<br>*G. c. tippelskirchi*
**2007**	Brown et al.	**(1)** *Giraffa reticulata*<br>**(2)** *Giraffa rothschildi*<br>**(3)** *Giraffa peralta*<br>**(4)** *Giraffa tippelskirchi*<br>**(5)** *Giraffa giraffa*<br>**(6)** *Giraffa angolensis*	-*
**2011**	Grubb and Groves	**(1)** *Giraffa camelopardalis*	**(1)** *G. c. camelopardalis*<br>*G. c. rothschildi*
		**(2)** *Giraffa antiquorum*	**(2)** *G. a. antiquorum*
		**(3)** *Giraffa peralta*	**(3)** *G. p. peralta*
		**(4)** *Giraffa reticulata*	**(4)** *G. r. reticulata*
		**(5)** *Giraffa tippelskirchi*	**(5)** *G. t. tippelskirchi*
		**(6)** *Giraffa thornicrofti*	**(6)** *G. t. thornicrofti*
		**(7)** *Giraffa giraffa*	**(7)** *G. g. giraffa*
		**(8)** *Giraffa angolensis*	**(8)** *G. a. angolensis*
**2016**	Fennessy et al.	**(1)** *Giraffa camelopardalis*	**(1)** *G. c. camelopardalis*<br>*G. c. antiquorum*<br>*G. c. peralta*<br>*G. c. rothschildi*
		**(2)** *Giraffa reticulata*	**(2)** *G. r. reticulata*
		**(3)** *Giraffa tippelskirchi*	**(3)** *G. t. tippelskirchi*<br>*G. t. thornicrofti*
		**(4)** *Giraffa giraffa*	**(4)** *G. g. angolensis*<br>*G. g. giraffa*

*all used subspecies were elevated to species rank

References:

Brisson MJ. Regnum animale in classes IX distributum sive Synopsis methodica. Haak, Paris, Leiden; 1762.

Brown DM, Brenneman RA, Koepfli KP, Pollinger JP, Milá B, Georgiadis NJ, et al. Extensive population genetic structure in the giraffe. BMC Biol. 2007; 5:1-13.

Dagg AI, Foster JB. The giraffe: its biology, behaviour, and ecology. New York: Van Nostrand Reinhold Company; 1976.

De Winton WE. On the giraffe of Somaliland. J Nat Hist. 1899; 4: 211-212.

Fennessy J, Bidon T, Reuss F, Kumar V, Elkan P, Nilsson MA, et al. Multi-locus analyses reveal four giraffe species instead of one. Curr Biol. 2016; 26: 2543-2549.

Groves C, Grubb P. Ungulate taxonomy. Maryland: JHU Press; 2011.

IUCN Red List of Threatened Species. Available from: http://www.iucnredlist.org

Kingdon J, Happold D, Butynski T, Hoffmann M, Happold M, Kalina J. Mammals of Africa. New York: Bloomsbury Publishing; 2013.

Krumbiegel I. Die Giraffe (*Giraffa camelopardalis*). Lutherstadt Wittenberg: A. Ziemsen Verlag; 1971.

Linnaeus CV. Systema Naturae. X ed. vol. 1. Holmiae Salvii; 1758.

Lydekker R. Catalogue of the ungulate mammals in the British Museum (Natural History). 3rd ed. London: British Museum Trustees; 1914. pp. 234-257.

Saint-Hilaire G. Quelques considérations sur la Girafe. Ann Sci Nat. 1827; 210-237.

Thomas O. On the five-horned Giraffe obtained by Sir Marry Johnston near Mount Elgon. J Zool. 1901; 71: 474-483.
