# Supplementary material for: A comparative approach for species delimitation based on multiple methods of multi-locus DNA sequence analysis: A case study of the genus Giraffa (Mammalia, Cetartiodactyla)
Source: PLoS One. 2020 Feb 13;15(2):e0217956. doi: 10.1371/journal.pone.0217956 (PMC7018015; doi:10.1371/journal.pone.0217956)
Supplement: S2 Appendix — (PDF) [file pone.0217956.s002.pdf]

S2 Appendix. Phylogenetic analyses

A comparative approach for species delimitation based on multiple methods of multi-locus DNA sequence analysis: a case study of the genus *Giraffa* (Mammalia, Cetartiodactyla)

Alice Petzold[1,2], Alexandre Hassanin[1,2]*

1 Institut de Systématique, Évolution, Biodiversité (ISYEB), Sorbonne Université, MNHN, CNRS, EPHE, Paris, France

2 Muséum national d'Histoire naturelle, CP51, 55 rue Buffon - 75005 Paris, France

*Correspondence: alexandre.hassanin@mnhn.fr

Table A. Overview of the 82 haplotypes used for the mitochondrial analyses, including their code, accession number, and the list of individuals. In case of independent upload of *cytochrome b* and *D-Loop* sequence, respective accession numbers are separated by slash (*cytochrome b* / *D-Loop*).

n	Haplotype	Representative individual	∑	Geographic origin (frequency)	Accession numbers
1	**RET1**	EU088319_*reticulata*_3	**22**	Ol Jogi, Laikipia, Kenya **(7)**; Pyramid, Kenya **(5)**; Sweetwaters, Laikipia, Kenya **(1)**; Meru NP, Kenya **(5)**; Samburu NR, Kenya **(2)**; Stuttgart Zoo, Germany **(2)**	**EU088319:** MeruReG10, SamburuRe4810, MeruReG11, MeruReG3, OlJogiReR8, MeruReG9, OlJogiRe27, SweetwaRe8, PyramidRe9, PyramidRe11, PyramidRe12, SamburuRe5, PyramidRe5, OlJogiRe13, OlJogiRe9, MeruReG7, OlJogiReR5, PyramidRe7, OlJogiRe15, OlJogiRe2; **LT628387/ LT628414**; **MG262296/ MG257963**
2	**RET2**	RET_RetNue3	1	Nuernberg Zoo **(1)**	**LT628410/ LT628383**
3	**RET3**	EU088320_*reticulata*_4	**15**	Ol Jogi, Laikipia, Kenya **(4)**; Pyramid, Kenya **(2)**;Sweetwaters, Laikipia, Kenya **(2)**; Samburu NR, Kenya **(7)**	**EU088320:** SamburuRe4812, SamburuRe4813, SamburuRe4816, OlJogiReR6, SamburuRe4815, SamburuRe4818, SamburuRe4819, OlJogiRe25, SweetwaRe1, SweetwaRe2, OlJogiRe10, OlJogiRe12, PyramidRe3, SamburuRe4, PyramidRe8
4	**RET4**	EU088322_*reticulata*_6	**13**	Ol Jogi, Laikipia, Kenya **(3)**; Pyramid, Kenya **(2)**; Sweetwaters, Laikipia, Kenya **(6)**; Meru NP, Kenya **(1)**; Samburu NR, Kenya **(1)**	**EU088322:** SamburuRe4817, OlJogiRe21, MeruReG4, SweetwaReGS44, SweetwaRe6, SweetwaReGS43, SweetwaRe11, SweetwaRe5, SweetwaRe4, OlJogiRe3, PyramidRe10, PyramidRe1, OlJogiRe4
5	**RET5**	EF442272_*reticulata*_Zenith	7	Singean African Reserve, France **(2)**; Nuernberg Zoo, Germany **(3)**; Pyramid, Kenya **(1)**; Rotterdam Zoo, Netherlands **(1)**	**EF442272:** Zenith, Désirée; **EU088325:** PyramidRe4; **LT628409/ LT628382**; **LT628411/ LT628384**; **LT628413/ LT628386**; **MG262293/ MG257960**

**6**	**RET6**	EU088324_*reticulata*_8	**2**	Pyramid, Laikipia, Kenya **(2)**	**EU088324:** PyramidRe2, PyramidRe6
**7**	**RET7**	EU088323_*reticulata*_7	**2**	Sweetwaters, Laikipia, Kenya **(1)**; Rotterdam Zoo, Netherlands **(1)**	**EU088323**; **MG262294/ MG257961**
**8**	**RET8**	RET_RetNue5	**3**	Nuernberg Zoo, Germany **(1)**; Rotterdam Zoo, Netherlands **(1)**; Loisaba Wildlife Conservancy, Kenya **(1)**	**LT628412/ LT628385**; **MG262295/ MG257962**; **MG262285/ MG257952**
**9**	**RET9**	EU088321_*reticulata*_5	**1**	**?**	**?**
**10**	**RET10**	*reticulata*_ISC03	**1**	Ishqbini Conservancy, Kenya **(1)**	**KY865164/ KY865127**
**11**	**RET11**	*reticulata*_ISC04	**1**	Ishqbini Conservancy, Kenya **(1)**	**KY865165/ KY865128**
**12**	**RET12**	*reticulata*_ISC05	**1**	Ishqbini Conservancy, Kenya **(1)**	**KY865166/ KY865129**
**13**	**RET13**	*reticulata*_ISC08	**1**	Ishqbini Conservancy, Kenya **(1)**	**KY865167/ KY865130**
**14**	**PER1**	EF442274_*peralta*	**28**	Harikanassou Region, Niger **(15)**; Koure, Niger **(12)**; Niamey-Dosso Road, Niger **(1)**	**EU088317:** Niger15, Niger16, Niger17, Niger19, Niger10, Niger22, Niger23, Niger28, Niger7, Niger12, Niger13, Niger14, Niger2B, Niger3C, Niger21; **EF442274:** PER1, PER2, PER3, DOSSO; **HG975175/ HG975277**; **HG975176/ HG975278**; **HG975180/ HG975282**; **HG975181/ HG975283**; **HG975183/ HG975285**; **HG975184/ HG975286**; **HG975185/ HG975287**; **MG262299/ MG257966**; **MG262297/ MG257964**
**15**	**PER2**	*peralta*_WA36	**1**	Koure, Niger **(1)**	**HG975177/ HG975279**
**16**	**PER3**	*peralta*_WA623	**2**	Koure, Niger **(2)**	**HG975186/ HG975288; HG975188/ HG975290**
**17**	**PER4**	EU088318_*peralta*_2	**12**	Harikanassou Region, Niger **(7)**; Koure, Niger **(5)**	**EU088318:** Niger20, Niger26, Niger27, Niger18, Niger5, Niger1A, Niger4D; **HG975178/ HG975280**; **HG975182/ HG975284**; **HG975187/ HG975289**; **MG262301/ MG257968**; **MG262300/ MG257967**
**18**	**PER5**	*peralta*_WA705	**1**	Koure, Niger **(1)**	**MG262298/ MG257965**

19	**ROTH1**	EF442273_*rothschildi*_Roby	**59**	Nakuru NP; Kenya **(7)**; Ruma, Kenya **(9)**; Thoiry Zoological Park, France **(1)**; La Palmyre Zoo, France **(1)**; Murchison Falls NP, Uganda **(41)**	**EF442273:** Eole_1137, Roby_A4216; **EU088326:** NakuruRoGN13, NakuruRoGN15, RumaRoGR33, RumaRoGR34, RumaRoGR38, RumaRoGR39, RumaRoGR40, NakuruRoGN16, NakuruRoGN18, NakuruRoGN19, NakuruRoGN23, NakuruRoGN28, RumaRoGR36, RumaRoGR37, RumaRoGR42, RumaRoGR31, MFNPF3Roth, MFNPF4Roth, MFNPF5Roth, MFNPF7Roth, MFNPF9Roth, MFNPF10Roth, MFNPF12Roth, MFNPF13Roth, MFNPF11Roth, MFNPF14Roth, MFNPF15Roth, MFNPF16Roth, MFNPM1Roth, MFNPM2Roth, MFNPM3Roth, MFNPM4Roth, MFNPM5Roth, MFNPM8Roth, MFNPM17Roth, MFNPM18Roth, MFNPM19Roth, MFNPM20Roth, MFNPM22Roth, MFNPM23Roth, MFNPM24Roth**;** **HG975130/ HG975232**; **HG975131/ HG975233**; **HG975132/ HG975234**; **HG975133/ HG975235**; **HG975134/ HG975236**; **HG975135/ HG975237**; **HG975136/ HG975238**; **HG975137/ HG975239**; **HG975138/ HG975240**; **MG262286/ MG257953**; **MG262287/ MG257954**; **MG262288/ MG257955**; **MG262289/ MG257956**; **MG262290/ MG257957**; **MG262291/ MG257958**; **MG262292/ MG257959**
20	**ROTH2**	EU088327_*rothschildi*_11	**2**	Nakuru NP, Kenya **(1)**; Ruma, Kenya **(1)**	**EU088327:** NakuruRoGN14, RumaRoGR32
21	**ROTH3**	EU088328_*rothschildi*_12	**8**	Ruma, Kenya **(2)**; Nakuru NP, Kenya **(6)**	**EU088328:** NakuruRoGN24, RumaRoGR41, NakuruRoGN17, NakuruRoGN20, NakuruRoGN22, NakuruRoGN29, NakuruRoGN30, RumaRoGR35
22	**CAM1**	*camelopardalis*_BaNP4	**1**	Badingilo National Park, South Sudan **(1)**	**LT628396/ LT628369**
23	**CAM2**	*camelopardalis*_ETH1	**1**	Gambella National Park, Ethiopia **(1)**	**LT628397/ LT628370**
24	**CAM3**	*camelopardalis*_BaNP3	**1**	Badingilo National Park, South Sudan **(1)**	**LT628395/ LT628368**
25	**CAM4**	*camelopardalis*_ETH2	**2**	Gambella National Park, Ethiopia **(2)**	**MG262281/ MG257948**; **MG262282/ MG257949**
26	**ANT1**	*antiquorum*_GNP04	**2**	Garamba National Park, DRC **(2)**	**HG975129/ HG975231**; **MG262284/ MG25795**
27	**ANT2**	EF442265_*antiquorum*_Rafiki	**2**	Vincennes Zoo, Paris **(2)**	**EF442265:** Rafiki_ZA4128, Uma_ZA4124
28	**ANT3**	EF442268_*antiquorum*_Waza	**3**	Waza NP, Cameroon **(2)**, Bouba Njida NP, Cameroon **(1)**	**EF442268:** WAZA1, WAZA2, BOUBA
29	**ANT4**	EF442266_*antiquorum*_Zakouma	**2**	Zakouma NP, Chad **(2)**	**EF442266:** ZAK1, ZAK2
30	**ANT5**	*antiquorum*_ZNP01	**1**	Zakouma NP, Chad **(1)**	**HG975179/ HG975281**
31	**ANT6**	EF442267_*antiquorum*_Sarah	**3**	Antwerp Zoo, Belgium **(1)**; Vincennes Zoo, France **(1)**; Shambe National Park, South Sudan **(1)**	**EF442267:** Sarah_M9271, Valere_M9633B**; LT628417/ LT628390**
32	**ANT7**	*antiquorum*_SNR1	**4**	Shambe National Park, South Sudan **(1)**; Garamba National Park, Democratic Republic of Congo (DRC) **(3)**	**LT628389/ LT628416**; **HG975127/ HG975229**; **HG975128/ HG975230**; **MG262283/ MG257950**

**33**	**THO1**	*thornicrofti*_LVNP8_01	**34**	Luangwa Valley NP, Zambia **(34)**	**HF571134 - HF571167** / **HF571176 - HF571209**
**34**	**TIP1**	EU088330_*tippelskirchi*_14	**16**	Seronera Serengeti NP, Tanzania **(3)**; Ndutu Serengeti NP, Tanzania **(7)**; Lobo Serengeti NP, Tanzania **(4)**; Manyara NP, Tanzania **(2)**	**EU088330:** SeroneraM1404, SeroneraM4735, SeroneraM4741, NdutuM4860, NdutuM4861, NdutuM4858, NdutuM4865, NdutuM4864, NdutuM4868, NdutuM4869, Lobo2097, Lobo2054, Lobo2067, Lobo2068, Manyara4845, Manyara4846
**35**	**TIP2**	EU088337_*tippelskirchi*_21	**3**	Seronera Serengeti NP, Tanzania **(1)**; Ndutu Serengeti NP, Tanzania **(2)**	**EU088337:** SeroneraM4743, NdutuM4862, NdutuM4870
**36**	**TIP3**	EU088340_*tippelskirchi*_24	**1**	Lobo Serengeti NP, Tanzania **(1)**	**EU088340**
**37**	**TIP4**	EU088338_*tippelskirchi*_22	**3**	Ndutu Serengeti NP, Tanzania **(2)**; Ngorongoro Serengeti NP, Tanzania **(1)**	**EU088338:** NdutuM4859, NdutuM4867, NgorogoroM4903
**38**	**TIP5**	EU088339_*tippelskirchi*_23	**1**	Varicho, Serengeti NP; Tanzania **(1)**	**EU088339**
**39**	**TIP6**	EF442269_*tippelskirchi*_Saburi	**36**	Basel Zoo, Switzerland **(2)**; Athi River Ranch, Kenya **(5)**; Chyulu Hills, Kenya **(13)**; Manyara NP, Tanzania **(5)**; Lake Naivasha, Tanzania **(4)**; Tarangire NP; Tanzania **(7)**	**EF442269:** Wari_990681, Saburi_950677; **EU088329:** TarangireM2244, TarangireM2232, ChyuluM21, AthiM7, ChyuluM12, ChyuluM22, ChyuluM5, AthiM6, AthiM3, AthiM14, ChyuluM1, ChyuluM11, ChyuluM17, ChyuluM18, ChyuluM19, ChyuluM2, ChyuluM24, ChyuluM6, NaivashaM5021, NaivashaM5026, NaivashaM5022, ManyaraM4848, Chyulu16, TarangireM2245, TarangireM2274, AthiM24, TarangireM2234, TarangireM2236, TarangireM2267, NaivashaM5018, Manyara4841, Manyara4842, Manyara4843, Manyara4847
**40**	**TIP7**	EU088335_*tippelskirchi*_19	**1**	Athi River Ranch Kenya **(1)**	**EU088335**
**41**	**TIP8**	*tippelskirchi*_SGR01	**6**	Selous Game Reserve, Tanzania **(6)**	**HG975158/ HG975260**; **HG975159/ HG975261**; **HG975160/ HG975262**; **HG975161/ HG975263**; **HG975162/ HG975264**; **HG975163/ HG975265**
**42**	**TIP9**	EU088342_*tippelskirchi*_26	**2**	Tarangire NP, Tanzania **(2)**	**EU088342:** TarangireM2265, TarangireM2251
**43**	**TIP10**	EU088331_*tippelskirchi*_15	**7**	Chyulu Hills, Kenya **(2)**; Manyara NP, Tanzania **(2)**; Tarangire NP, Tanzania **(1)**; Lake Naivasha, Tanzania **(2)**	**EU088331:** TarangireM2271, ChyuluM23, ChyuluM4, NaivashaM5016, NaivashaM5017, Manyara4840, Manyara4839
**44**	**TIP11**	EU088333_*tippelskirchi*_17	**2**	Athi River Ranch, Kenya **(2)**	**EU088333:** AthiM18, AthiM1
**45**	**TIP12**	EU088332_*tippelskirchi*_16	**1**	Tarangire NP, Tanzania **(1)**	**EU088332**
**46**	**TIP13**	EU088341_*tippelskirchi*_25	**2**	Tarangire NP, Tanzania **(2)**	**EU088341:** TarangireM2237, TarangireM2259
**47**	**TIP14**	EU088336_*tippelskirchi*_20	**1**	Chyulu Hills, Kenya **(1)**	**EU088336**
**48**	**TIP15**	EU088334_*tippelskirchi*_18	**9**	Athi River Ranch, Kenya **(9)**	**EU088334:** AthiM8, AthiM21, AthiM5, AthiM13, AthiM2, AthiM11, AthiM15, AthiM22, AthiM23

**49**	**GFA1**	EF442271_*giraffa*_Phalaborwa	**51**	Kruger National Park, South Africa **(10)**; Serondella Zimbabwe **(1)**; Phalabowa Limpopo, South Africa **(1)**; Moremi Game Reserve, Botswana **(5)**; Sun Hotel Livingstone, Zambia **(4)**; Vumbura Concession, Botswana **(10)**; Chobe National Park, Botswana **(7)**; Mosi-oa-Tunya NP, Zambia **(10)**; Khamab Kalahari Reserve, South Africa **(3)**	**EF442271**; **EU088343:** Botswana903, Kruger9, Kruger5, Kruger7, Kruger8, Kruger20, Kruger25, Kruger27, Kruger1, Kruger12, Kruger14; **HF571212**; **HF571215**; **HF571216**; **HG975203**; **HG975207**; **HG975208**; **HG975209**; **HG975211**; **HG975241**; **HG975245**; **HG975247**; **HG975248**; **HG975255**; **HG975257**; **HG975258**; **HG975266**; **HG975267**; **HG975268**; **HG975269**; **HG975270**; **HG975271**; **HG975273**; **HG975274**; **HG975275**; **HG975276**; **LT628398/ LT628371**; **LT628399/ LT628372**; **LT628400/ LT628373**; **LT628401/ LT628374**; **LT628402/ LT628375**; **LT628404/ LT628377**; **LT628405/ LT628378**; **LT628406/ LT628379**; **LT628407/ LT628380**; **LT6284087/ LT62838**; **LT628418/ LT628391**; **LT628419/ LT628392**; **LT628420/ LT628393**; **LT628421/ LT628394**
**50**	**GFA2**	*giraffa* _SNNP	**1**	Sioma Ngwezi NP, Zambia **(1)**	**LT628415/ LT628388**
**51**	**GFA3**	*giraffa*_BW01	**7**	Bwabwata National Park, Namibia **(7)**	**HG975087/ HG975189**; **HG975088/ HG975190**; **HG975089/ HG975191**; **HG975090/ HG975192**; **HG975091/ HG975193**; **HG975092/ HG975194**; **HG975093/ HG975195**
**52**	**GFA4**	*giraffa*_CNP9_08	**1**	Chobe NP, Botswana **(1)**	**HG975104/ HG975206**
**53**	**GFA5**	*giraffa*_MGR9_20	**1**	Moremi Game Reserve, Botswana **(1)**	**HG975151/ HG975253**
**54**	**GFA6**	*giraffa* _MTNP6	**1**	Mosi-oa-Tunya NP, Zambia **(1)**	**LT628403/ LT628376**
**55**	**GFA7**	EF442270_*giraffa*_Tango	**5**	Thoiry Reserve, France **(1)**; Kruger National Park, South Africa **(1)**; Khamab Kalahari Reserve, South Africa **(3)**	**EF442270**; **EU088344:** Kruger23; **HG975152/ HG975254**; **HG975154/ HG975256**; **HG975157/ HG975259**
**56**	**GFA8**	*giraffa*_CNP9_06	**1**	Chobe NP, Botswana **(1)**	**HG975102/ HG975204**
**57**	**GFA9**	*giraffa*_CNP9_07	**1**	Chobe NP, Botswana **(1)**	**HG975103/ HG975205**
**58**	**GFA10**	*giraffa*_MGR9_11	**4**	Moremi Game Reserve, Botswana **(2)**; Chobe NP, Botswana **(2)**	**HF571168/ HF571210**; **HF571169/ HF571211**; **HF571175/ HF571217**; **HG975108/ HG975210**
**59**	**GFA11**	*giraffa*_NXP9_01	**2**	Nxai Pans, Botswana **(1)**; Vumbura Concession, Botswana **(1)**	**HF571171/ HF571213**; **HG975170/ HG975272**
**60**	**GFA12**	*giraffa*_MGR9_05	**1**	Moremi Game Reserve, Botswana **(1)**	**HG975142/ HG975244**
**61**	**GFA13**	*giraffa*_MGR9_02	**7**	Moremi Game Reserve, Botswana **(7)**	**HG975140/ HG975242**; **HG975141/ HG975243**; **HG975144/ HG975246**; **HG975147/ HG975249**; **HG975148/ HG975250**; **HG975149/ HG975251**; **HG975150/ HG975252**
**62**	**ANG1**	EF442264_*angolensis*_Lisbon6A	**4**	Lisbon Zoo, Portugal **(1)**; Etosha NP, Namibia **(3)**	**EF442264**; **HG975110/ HG975212**; **HG975112/ HG975224**; **HG975125/ HG975227**
**63**	**ANG2**	EF442263_*angolensis*_Lisbon5A	**7**	Lisbon Zoo, Portugal **(1)**; Etosha NP, Namibia **(5)**; Hoarsib River, Namibia **(1)**	**EF442263**; **EU088350**: HSBM3; **HG975114/ HG975216**; **HG975116/ HG975218**; **HG975117/ HG975219**; **HG975119/ HG975221**; **HG975121/ HG975223**
**64**	**ANG3**	*angolensis*_ENP08	**2**	Etosha NP, Namibia **(2)**	**HG975115/ HG975217**; **HG975120/ HG975222**

**65**	**ANG4**	EU088345_*angolensis*_29	**13**	Etosha NP, Namibia **(13)**	**EU088345:** ENPF1, ENPF6, ENPF3, ENPF4, ENPM1, ENPM2, ENPM3, ENPM5, ENPF5, ENPF2; **EU088349:** ENPM7, ENPM4; **HG975228**
**66**	**ANG5**	EU088346_*angolensis*_30	**2**	Etosha NP, Namibia **(1)**; Kamanjab, Namibia **(1)**	**EU088346:** ENPF7, KMJM1
**67**	**ANG6**	EU088347_*angolensis*_31	**33**	Hoanib River, Namibia **(27)**; Hoarusib River, Namibia **(2)**; Northwestern Namibia **(4)**	**EU088347:** HNBF1, HNBF2, HNBF3, HNBF4, HNBF6, HNBF7, HNBF8, HNBM7, HNBM3, HNBM4, HNBM5, HNBM8, HNBM6, KHBM1, KHBM2, KHBM3, KHBF1; **KY865151/ KY865114**; **KY865152/ KY865115**; **KY865153/ KY865116**; **KY865154/ KY865117**; **KY865155/ KY865118**; **KY865156/ KY865119**; **KY865157/ KY865120**; **KY865158/ KY865121**; **KY865159/ KY865122**; **KY865160/ KY865123**; **KY865161/ KY865124**; **KY865162/ KY865125**; **KY865168/ KY865131**; **KY865171/ KY865134**; **KY865172/ KY865135**; **KY865173/ KY865136**
**68**	**ANG7**	EU088348_*angolensis*_32	**8**	Etosha NP, Namibia **(7)**; Hoanib River, Namibia **(1)**	**EU088348:** ENPM6, ENPF8; **HG975111/ HG975213**; **HG975112/ HG975214**; **HG975113/ HG975215**; **HG975123/ HG975225**; **HG975124/ HG975226**; **KY865149/ KY865112**
**69**	**ANG8**	EU088351_*angolensis*_35	**3**	Hoarusib River, Namibia **(1)**; Northwestern Namibia **(2)**	**EU088351**; **KY865170/ KY865133**; **KY865174/ KY865137**
**70**	**ANG9**	*angolensis*_CKGR9_01	**3**	Central Kalahari Game Reserve, Botswana **(3)**	**HG975094/ HG975196**; **HG975096/ HG975198**; **HG975098/ HG975200**
**71**	**ANG10**	*angolensis*_CKGR9_05	**1**	Central Kalahari Game Reserve, Botswana **(1)**	**HG975097/ HG975199**
**72**	**ANG11**	*angolensis*_CKGR9_02	**2**	Central Kalahari Game Reserve, Botswana **(2)**	**HG975095/ HG975197**; **HG975099/ HG975201**
**73**	**ANG12**	*angolensis*_ENP13	**1**	Etosha NP, Namibia **(1)**	**HG975118/ HG975220**
**74**	**ANG13**	*angolensis*_CKGR9 08	**1**	Central Kalahari Game Reserve, Botswana **(1)**	**HG975100/ HG975202**
**75**	**ANG14**	*angolensis*_NWN03	**1**	Northwestern Namibia **(1)**	**KY865169/ KY865132**
**76**	**ANG15**	*angolensis*_HSB04	**1**	Hoarusib River, Namibia **(1)**	**KY865163/ KY865126**
**77**	**ANG16**	*angolensis*_HNB001	**1**	Hoanib River, Namibia **(1)**	**KY865148/ KY865111**
**78**	**ANG17**	*angolensis*_HNB102	**1**	Hoanib River, Namibia **(1)**	**KY865150/ KY865113**
**79**	**ANG18**	*angolensis*_BVC001	**7**	Bubye Valley Conservancy, Zimbabwe **(7)**	**KY865138/ KY865101**; **KY865139/ KY865102**; **KY865140/ KY865103**; **KY865141/ KY865104**; **KY865143/ KY865106**; **KY865146/ KY865109**; **KY865147/ KY865110**
**80**	**ANG19**	*angolensis*_BVC005	**1**	Bubye Valley Conservancy, Zimbabwe **(1)**	**KY865142/ KY865105**
**81**	**ANG20**	*angolensis*_BVC007	**1**	Bubye Valley Conservancy, Zimbabwe **(1)**	**KY865144/ KY865107**
**82**	**ANG21**	*angolensis*_BVC008	**1**	Bubye Valley Conservancy, Zimbabwe **(1)**	**KY865145/ KY865108**

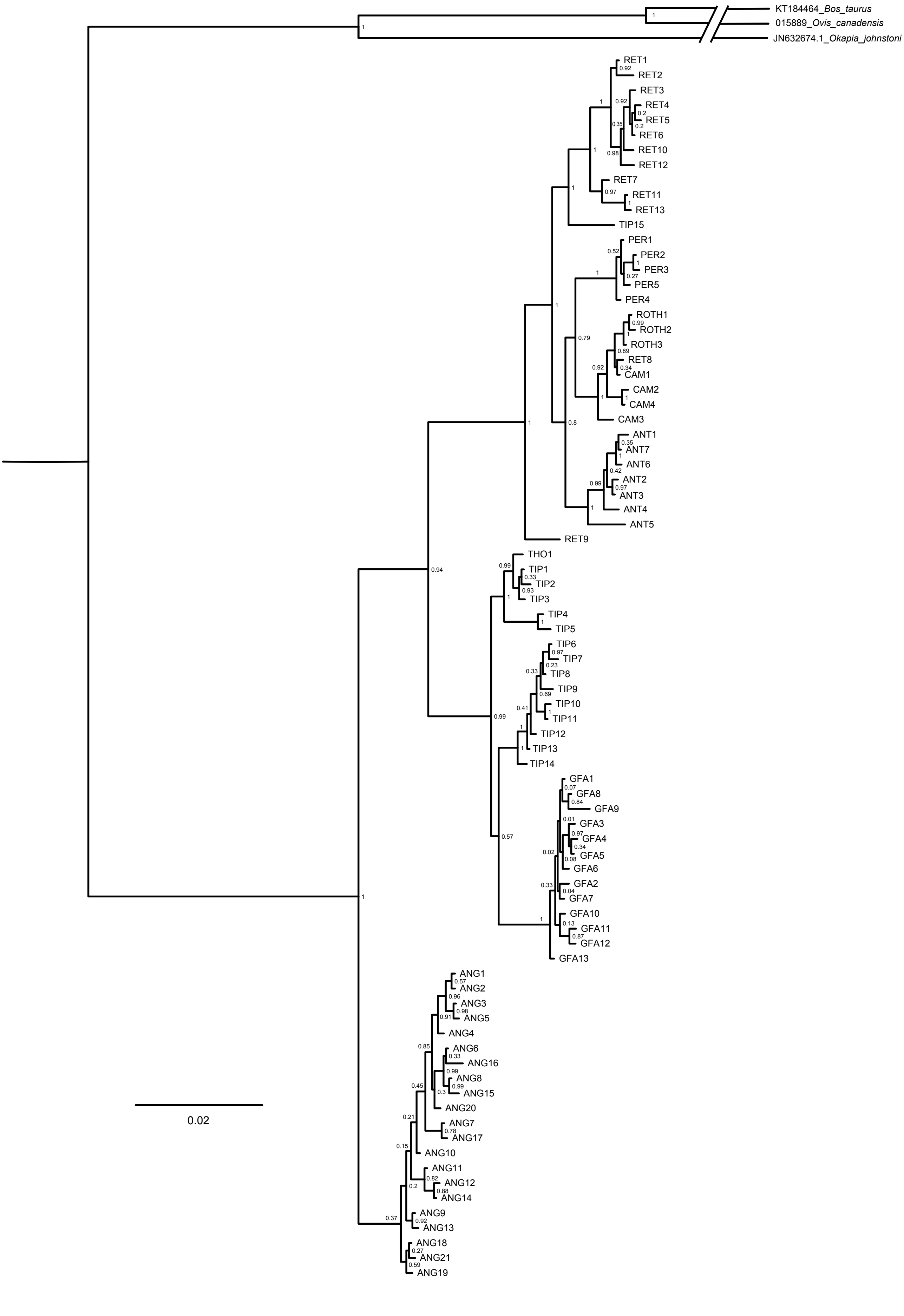

Fig A. Bayesian trees from the analyses of the mitochondrial fragment (*cytochrome b* and the partial 5' region) including 82 haplotypes of 507 giraffe individuals and three outgroup taxa. The posterior probability values are indicated for each node.

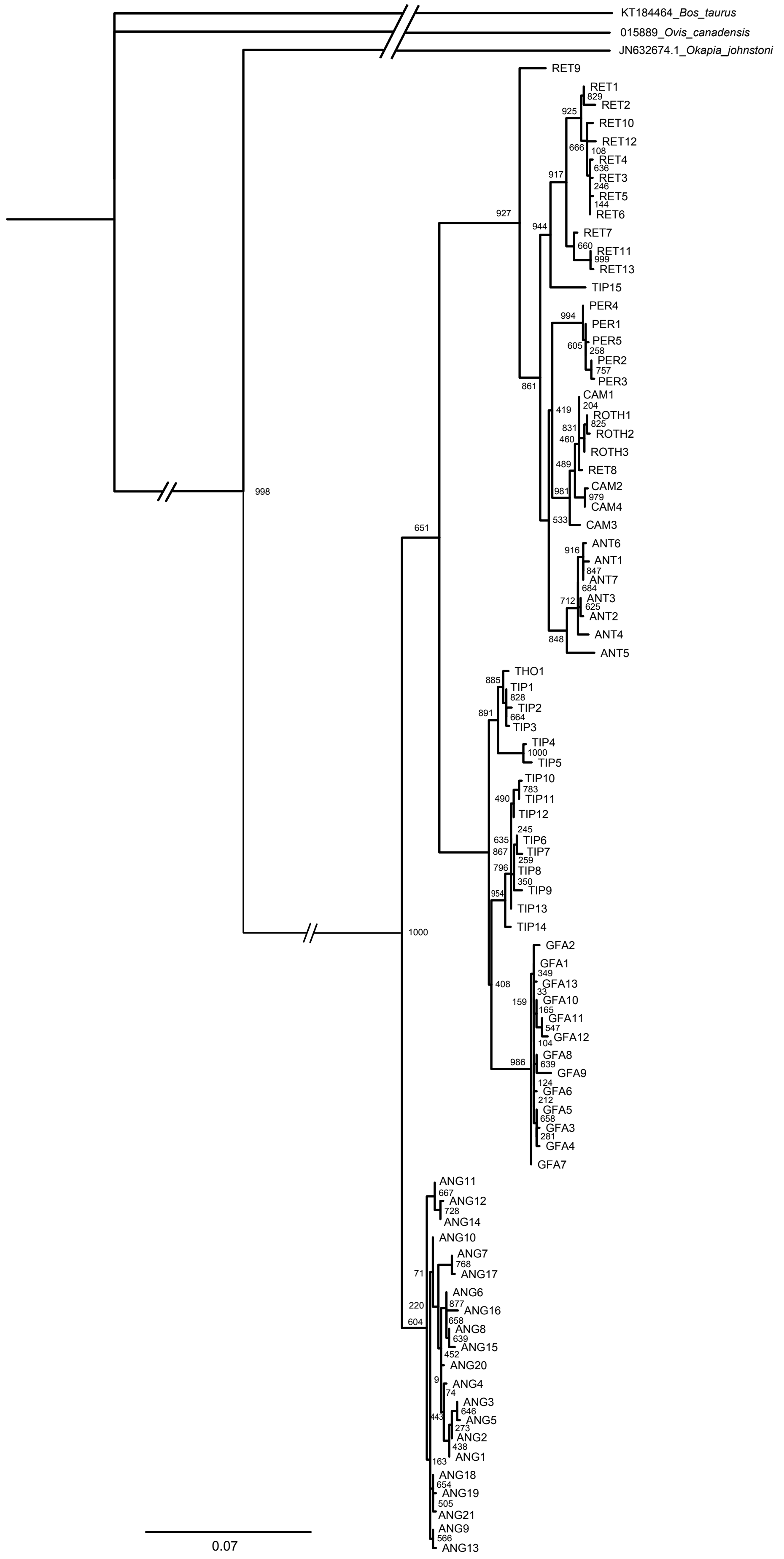

Fig B. PhyML trees from the Maximum Likelihood analyses of the mitochondrial fragment (*cytochrome b* and the partial 5' region) including 82 haplotypes from 507 giraffe individuals and three outgroup taxa.

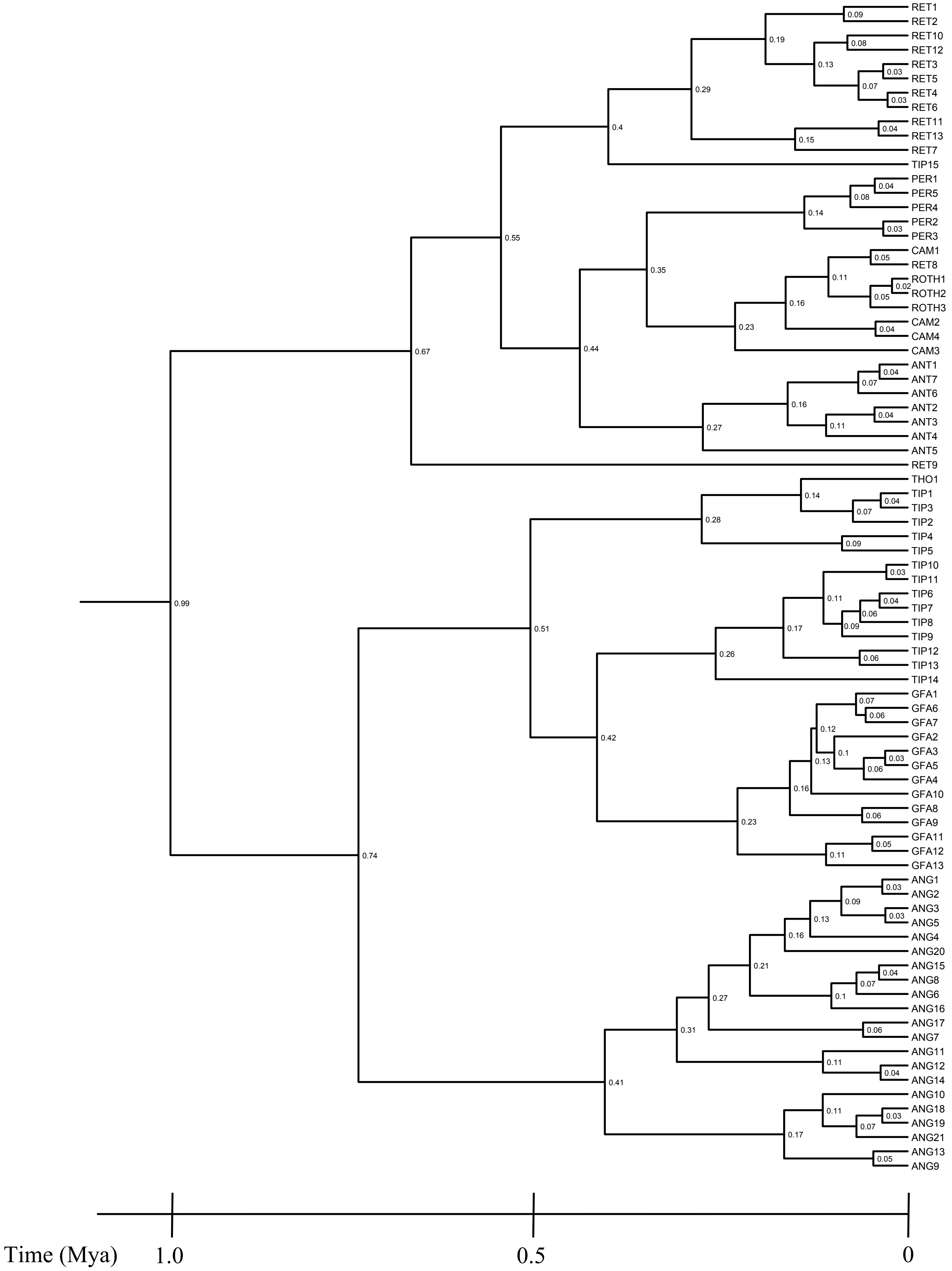

Fig C. Chronogram obtained from the analysis of the mitochondrial fragment (*cytochrome b* and the partial 5’ region) including 82 giraffe haplotypes and three outgroup taxa (not shown). The datations were estimated using BEAST v.2.4.7 with the following priors: relaxed clock Log normal; calibration for the MRCA of Giraffa = 1 +/ 0.1 Mya ; Yule model; 100000000 generations; Log and tree every 10000.

Table B. Individual code, designation of subspecies and origin of 137 giraffes used in the phylogenetic and population structure analyses of the nuclear dataset

Individual	Subspecies designation	Origin
**WA026**	*peralta*	Koure, Niger
**WA036**	*peralta*	Koure, Niger
**WA606**	*peralta*	Koure, Niger
**WA117**	*peralta*	Koure, Niger
**WA609**	*peralta*	Koure, Niger
**WA612**	*peralta*	Koure, Niger
**WA614**	*peralta*	Koure, Niger
**WA619**	*peralta*	Koure, Niger
**WA621**	*peralta*	Koure, Niger
**WA622**	*peralta*	Koure, Niger
**WA623**	*peralta*	Koure, Niger
**WA700**	*peralta*	Koure, Niger
**WA705**	*peralta*	Koure, Niger
**WA707**	*peralta*	Koure, Niger
**WA708**	*peralta*	Koure, Niger
**WA720**	*peralta*	Koure, Niger
**GNP01**	*antiquorum*	Garamba National Park, DR Congo
**GNP02**	*antiquorum*	Garamba National Park, DR Congo
**GNP03**	*antiquorum*	Garamba National Park, DR Congo
**GNP04**	*antiquorum*	Garamba National Park, DR Congo
**GNP05**	*antiquorum*	Garamba National Park, DR Congo
**SNR1**	*antiquorum*	Shambe National Park, South Sudan
**SNR2**	*antiquorum*	Shambe National Park, South Sudan
**ZNP01**	*antiquorum*	Zakouma National Park, Chad
**BaNP4**	*camelopardalis*	Badingilo National Park, South Sudan
**ETH1**	*camelopardalis*	Gambella National, Ethiopia
**ETH2**	*camelopardalis*	Gambella National, Ethiopia
**ETH3**	*camelopardalis*	Gambella National, Ethiopia
**MF01**	*rothschildi*	Murchison Falls National Park, Uganda
**MF02**	*rothschildi*	Murchison Falls National Park, Uganda
**MF03**	*rothschildi*	Murchison Falls National Park, Uganda
**MF04**	*rothschildi*	Murchison Falls National Park, Uganda
**MF05**	*rothschildi*	Murchison Falls National Park, Uganda
**MF06**	*rothschildi*	Murchison Falls National Park, Uganda
**MF07**	*rothschildi*	Murchison Falls National Park, Uganda
**MF09**	*rothschildi*	Murchison Falls National Park, Uganda
**MF11**	*rothschildi*	Murchison Falls National Park, Uganda
**MF13**	*rothschildi*	Murchison Falls National Park, Uganda
**MF14**	*rothschildi*	Murchison Falls National Park, Uganda
**MF15**	*rothschildi*	Murchison Falls National Park, Uganda
**MF16**	*rothschildi*	Murchison Falls National Park, Uganda
**MF17**	*rothschildi*	Murchison Falls National Park, Uganda
**MF24**	*rothschildi*	Murchison Falls National Park, Uganda

**RET1**	*reticulata*	Zoo
**RET3**	*reticulata*	Zoo
**RET4**	*reticulata*	Zoo
**RET5**	*reticulata*	Zoo
**RET6**	*reticulata*	Zoo
**RETWil**	*reticulata*	Zoo
**RETWil2**	*reticulata*	Zoo
**RETRot1**	*reticulata*	Zoo
**RETRot2**	*reticulata*	Zoo
**RETRot3**	*reticulata*	Zoo
**ISC03**	*reticulata*	Ishqbini Conservancy, Kenya
**ISC04**	*reticulata*	Ishqbini Conservancy, Kenya
**ISC08**	*reticulata*	Ishqbini Conservancy, Kenya
**LWC01**	*reticulata*	Loisaba Wildlife Conservancy, Kenya
**LVNP8_18**	*thornicrofti*	Luangwa Valley National Park, Zambia
**LVNP8_19**	*thornicrofti*	Luangwa Valley National Park, Zambia
**LVNP8_20**	*thornicrofti*	Luangwa Valley National Park, Zambia
**LVNP8_21**	*thornicrofti*	Luangwa Valley National Park, Zambia
**LVNP8_22**	*thornicrofti*	Luangwa Valley National Park, Zambia
**LVNP8_23a**	*thornicrofti*	Luangwa Valley National Park, Zambia
**LVNP8_31**	*thornicrofti*	Luangwa Valley National Park, Zambia
**LVNP8_32**	*thornicrofti*	Luangwa Valley National Park, Zambia
**LVNP8_33**	*thornicrofti*	Luangwa Valley National Park, Zambia
**LVNP8_34**	*thornicrofti*	Luangwa Valley National Park, Zambia
**LVNP8_35**	*thornicrofti*	Luangwa Valley National Park, Zambia
**LVNP8_36**	*thornicrofti*	Luangwa Valley National Park, Zambia
**SGR01**	*tippelskirchi*	Selous Game Reserve, Tanzania
**SGR05**	*tippelskirchi*	Selous Game Reserve, Tanzania
**SGR06**	*tippelskirchi*	Selous Game Reserve, Tanzania
**SGR07**	*tippelskirchi*	Selous Game Reserve, Tanzania
**SGR12**	*tippelskirchi*	Selous Game Reserve, Tanzania
**SGR13**	*tippelskirchi*	Selous Game Reserve, Tanzania
**SGR14**	*tippelskirchi*	Selous Game Reserve, Tanzania
**MTNP1**	*giraffa*	Mosi-oa-Tunya National Park, Zambia
**MTNP2**	*giraffa*	Mosi-oa-Tunya National Park, Zambia
**MTNP3**	*giraffa*	Mosi-oa-Tunya National Park, Zambia
**SNNP**	*giraffa*	Sioma Ngwezi NP, Zambia
**BNP01**	*giraffa*	Bwabwata National Park, Namibia
**BNP02**	*giraffa*	Bwabwata National Park, Namibia
**BNP03**	*giraffa*	Bwabwata National Park, Namibia
**BNP04**	*giraffa*	Bwabwata National Park, Namibia
**BNP05**	*giraffa*	Bwabwata National Park, Namibia
**BNP06**	*giraffa*	Bwabwata National Park, Namibia
**BNP09**	*giraffa*	Bwabwata National Park, Namibia
**CNP9_01**	*giraffa*	Chobe National Park, Botswana
**CNP9_02**	*giraffa*	Chobe National Park, Botswana
**CNP9_03**	*giraffa*	Chobe National Park, Botswana

**KKR01**	*giraffa*	Khamab Kalahari Reserve, South Africa
**KKR02**	*giraffa*	Khamab Kalahari Reserve, South Africa
**KKR03**	*giraffa*	Khamab Kalahari Reserve, South Africa
**KKR04**	*giraffa*	Khamab Kalahari Reserve, South Africa
**KKR05**	*giraffa*	Khamab Kalahari Reserve, South Africa
**KKR07**	*giraffa*	Khamab Kalahari Reserve, South Africa
**KKR08**	*giraffa*	Khamab Kalahari Reserve, South Africa
**MGR9_01**	*giraffa*	Moremi Game Reserve, Botswana
**MGR9_02**	*giraffa*	Moremi Game Reserve, Botswana
**MGR9_03**	*giraffa*	Moremi Game Reserve, Botswana
**MGR9_04**	*giraffa*	Moremi Game Reserve, Botswana
**MGR9_05**	*giraffa*	Moremi Game Reserve, Botswana
**SUN1**	*giraffa*	Sun hotel, Livingstone, Zambia
**SUN2**	*giraffa*	Sun hotel, Livingstone, Zambia
**SUN3**	*giraffa*	Sun hotel, Livingstone, Zambia
**SUN4**	*giraffa*	Sun hotel, Livingstone, Zambia
**V23**	*giraffa*	Vumbura Concession, Botswana
**V24**	*giraffa*	Vumbura Concession, Botswana
**V25**	*giraffa*	Vumbura Concession, Botswana
**V26**	*giraffa*	Vumbura Concession, Botswana
**V27**	*giraffa*	Vumbura Concession, Botswana
**V28**	*giraffa*	Vumbura Concession, Botswana
**V29**	*giraffa*	Vumbura Concession, Botswana
**V30**	*giraffa*	Vumbura Concession, Botswana
**V31**	*giraffa*	Vumbura Concession, Botswana
**V36**	*giraffa*	Vumbura Concession, Botswana
**V37**	*giraffa*	Vumbura Concession, Botswana
**V38**	*giraffa*	Vumbura Concession, Botswana
**V39**	*giraffa*	Vumbura Concession, Botswana
**CKGR9_01**	*angolensis*	Central Kalahari Game Reserve, Botswana
**CKGR9_02**	*angolensis*	Central Kalahari Game Reserve, Botswana
**CKGR9_03**	*angolensis*	Central Kalahari Game Reserve, Botswana
**CKGR9_05**	*angolensis*	Central Kalahari Game Reserve, Botswana
**ENP04**	*angolensis*	Etosha National Park, Namibia
**ENP07**	*angolensis*	Etosha National Park, Namibia
**ENP08**	*angolensis*	Etosha National Park, Namibia
**ENP09**	*angolensis*	Etosha National Park, Namibia
**ENP11**	*angolensis*	Etosha National Park, Namibia
**ENP12**	*angolensis*	Etosha National Park, Namibia
**ENP14**	*angolensis*	Etosha National Park, Namibia
**ENP15**	*angolensis*	Etosha National Park, Namibia
**ENP16**	*angolensis*	Etosha National Park, Namibia
**ENP17**	*angolensis*	Etosha National Park, Namibia
**ENP18**	*angolensis*	Etosha National Park, Namibia
**ENP19**	*angolensis*	Etosha National Park, Namibia
**ENP20**	*angolensis*	Etosha National Park, Namibia
**ENP21**	*angolensis*	Etosha National Park, Namibia

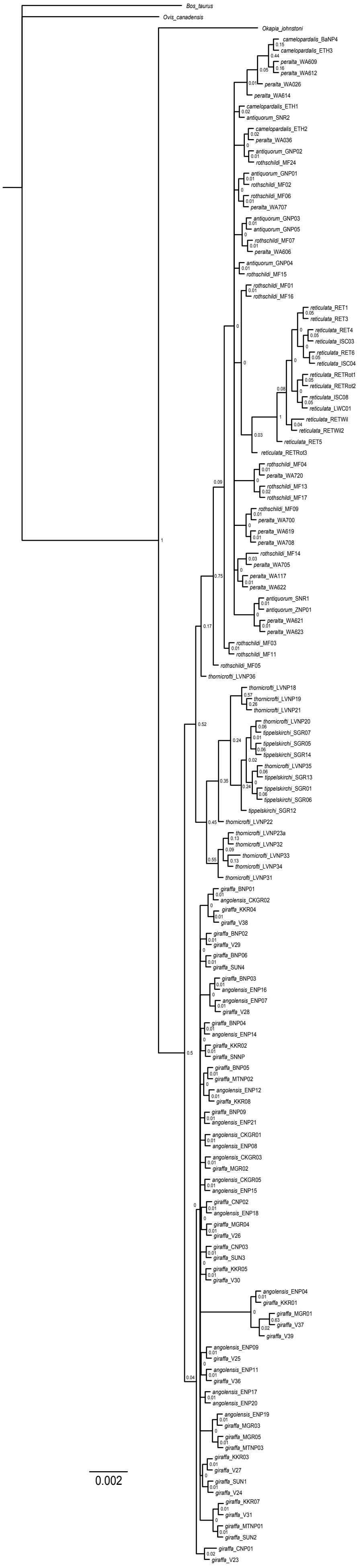

Fig D. Bayesian tree for the separate analysis of the 641 nucleotides (nt) long intron *ACP5* with the posterior probability values indicated for each node.

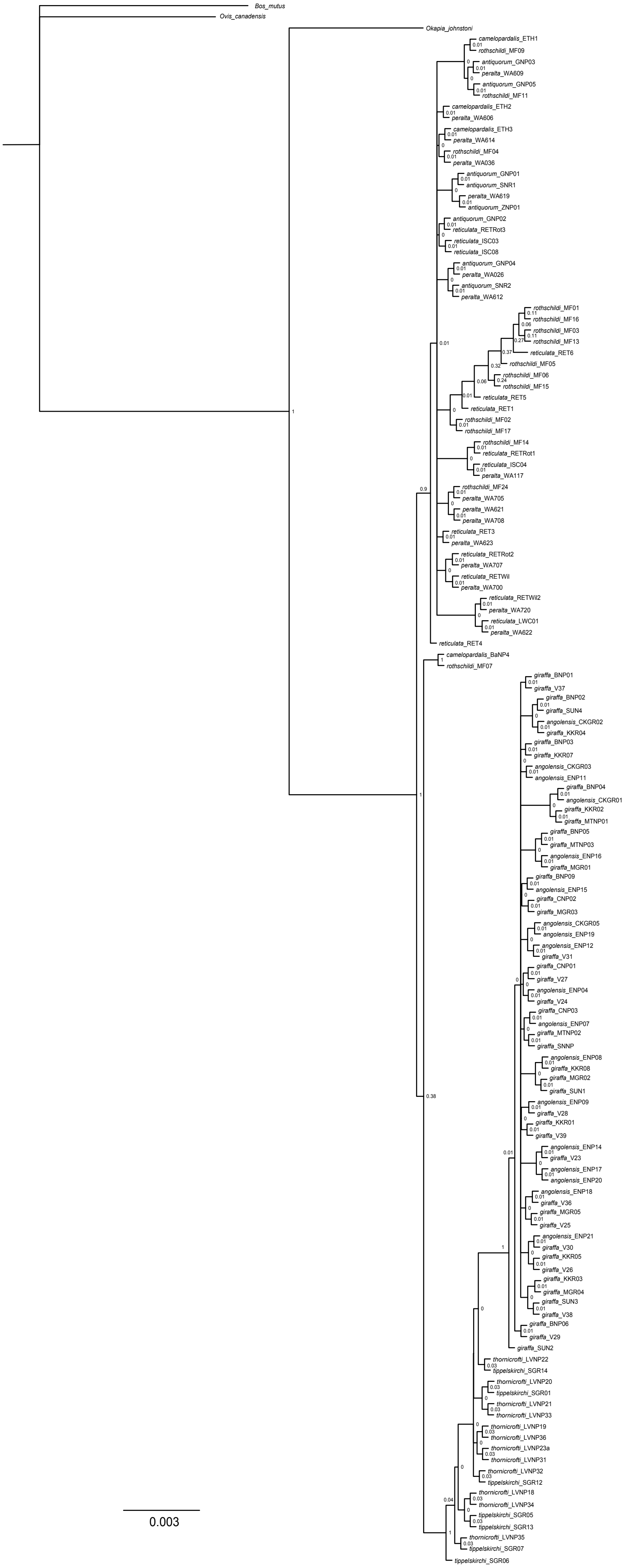

Fig E. Bayesian tree for the separate analysis of the 897 nucleotides (nt) long intron *C1orf74* with the posterior probability values indicated for each node.

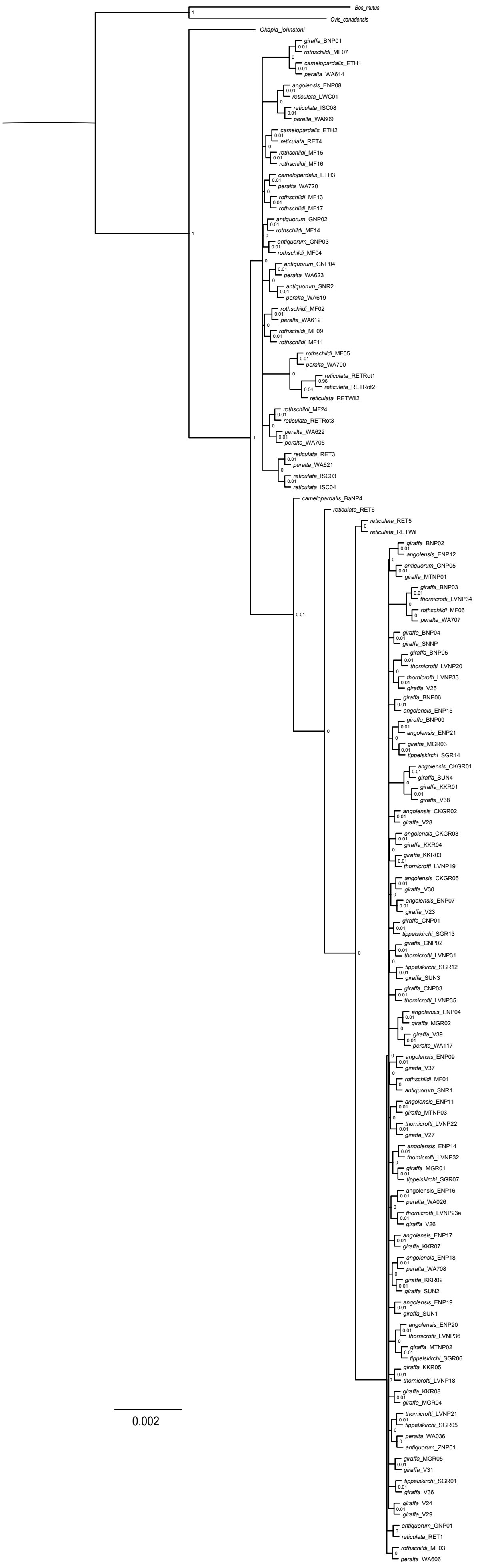

Fig F. Bayesian tree for the separate analysis of the 826 nucleotides (nt) long intron *CCT2* with the posterior probability values indicated for each node.

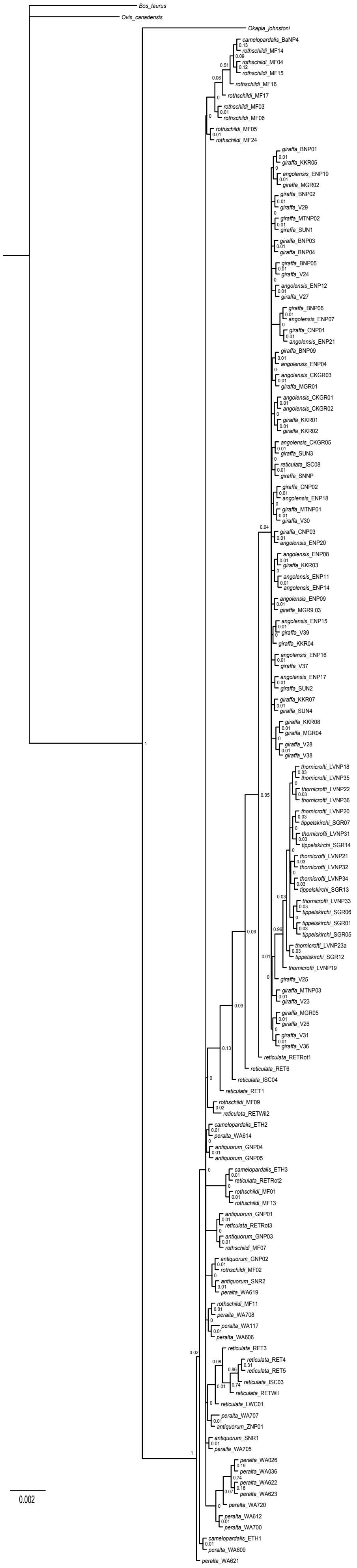

Fig G. Bayesian tree for the separate analysis of the 881 nucleotides (nt) long intron *COL5A2* with the posterior probability values indicated for each node.

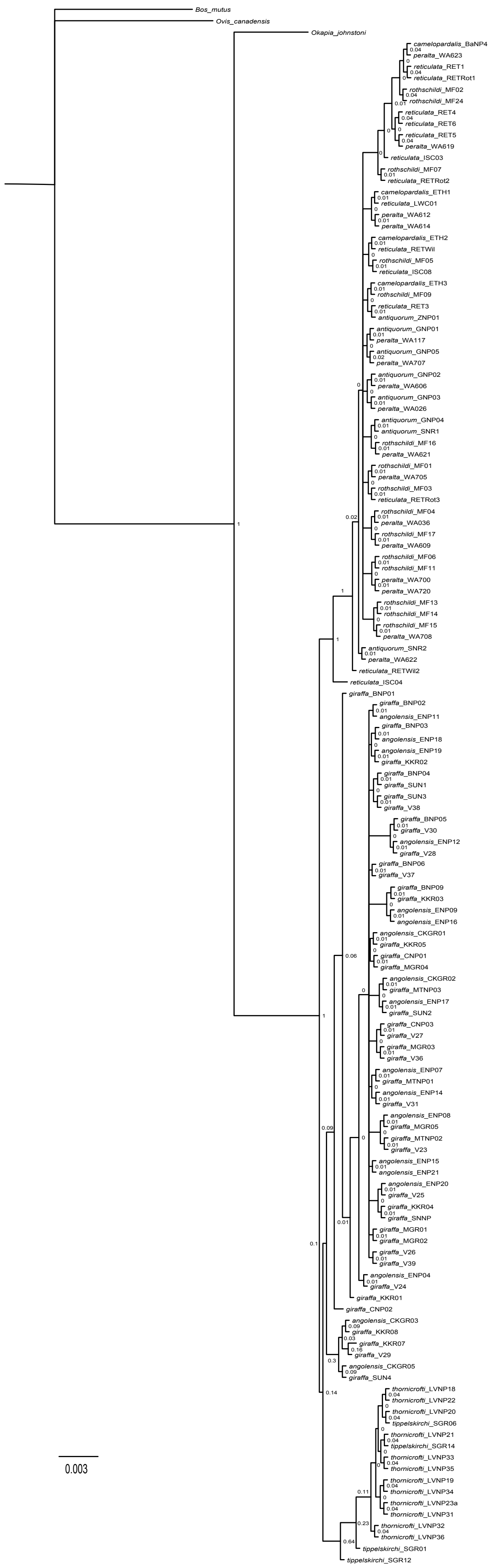

Fig H. Bayesian tree for the separate analysis of the 862 nucleotides (nt) long intron *CTAGE5* with the posterior probability values indicated for each node.

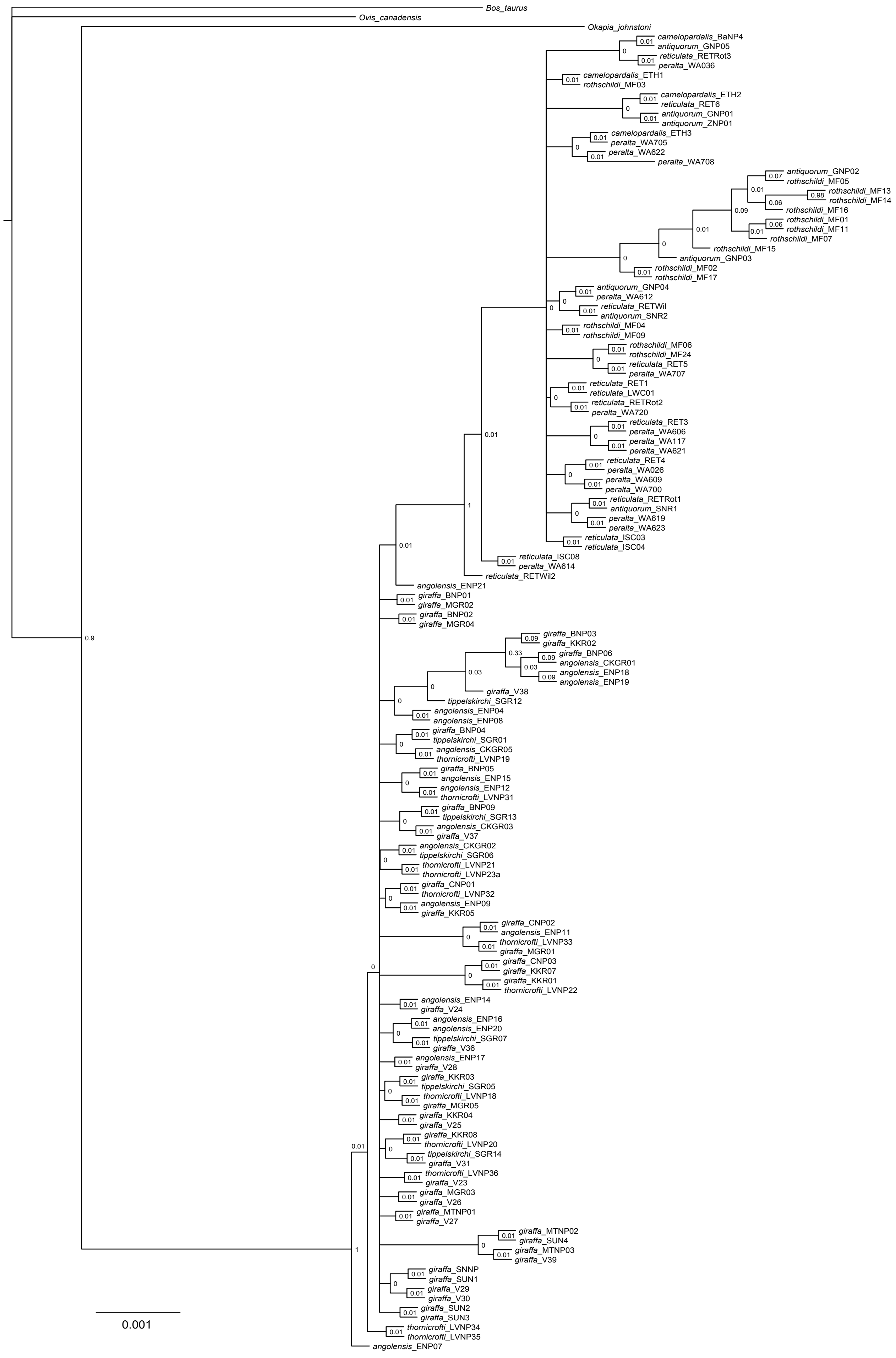

Fig I. Bayesian tree for the separate analysis of the 703 nucleotides (nt) long intron *CWF19L1* with the posterior probability values indicated for each node.

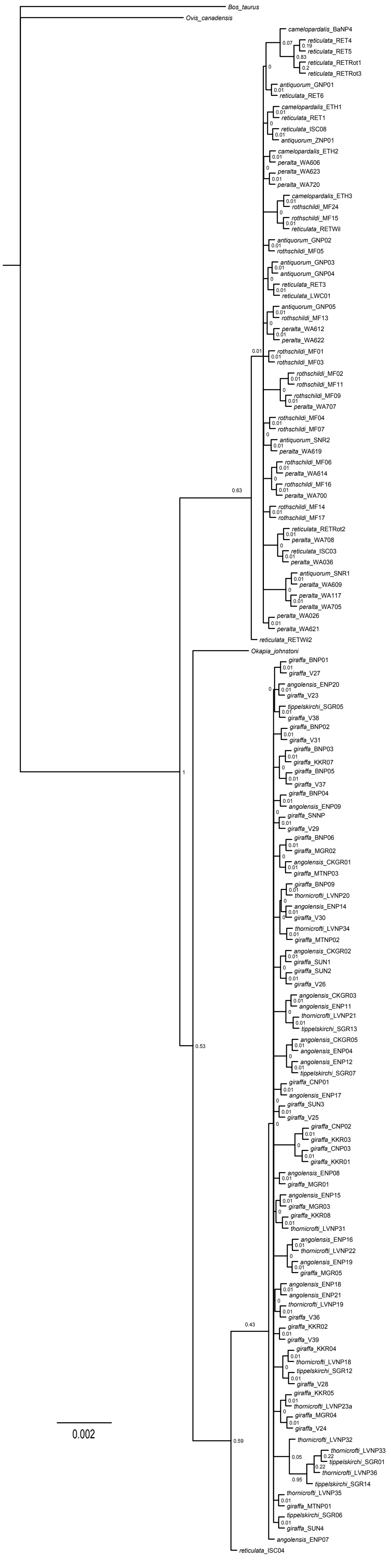

Fig J. Bayesian tree for the separate analysis of the 740 nucleotides (nt) long intron *DDX1* with the posterior probability values indicated for each node.

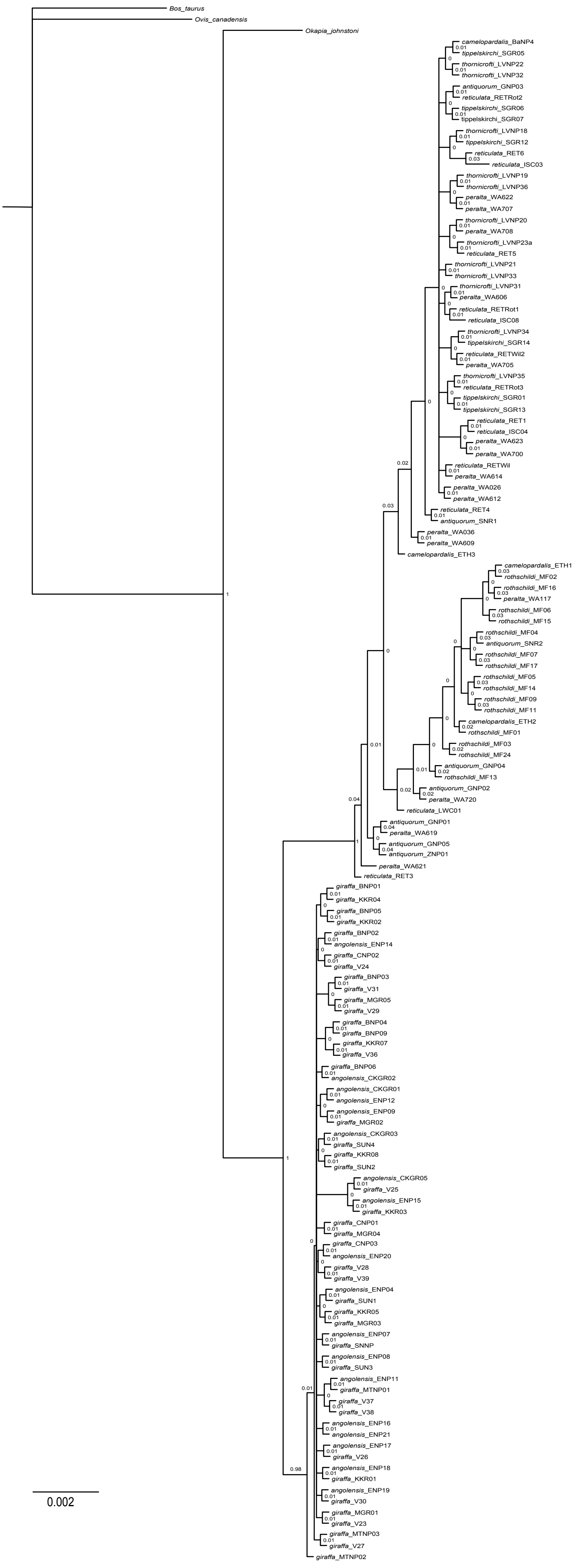

Fig K. Bayesian tree for the separate analysis of the 821 nucleotides (nt) long intron *DHX36* with the posterior probability values indicated for each node.

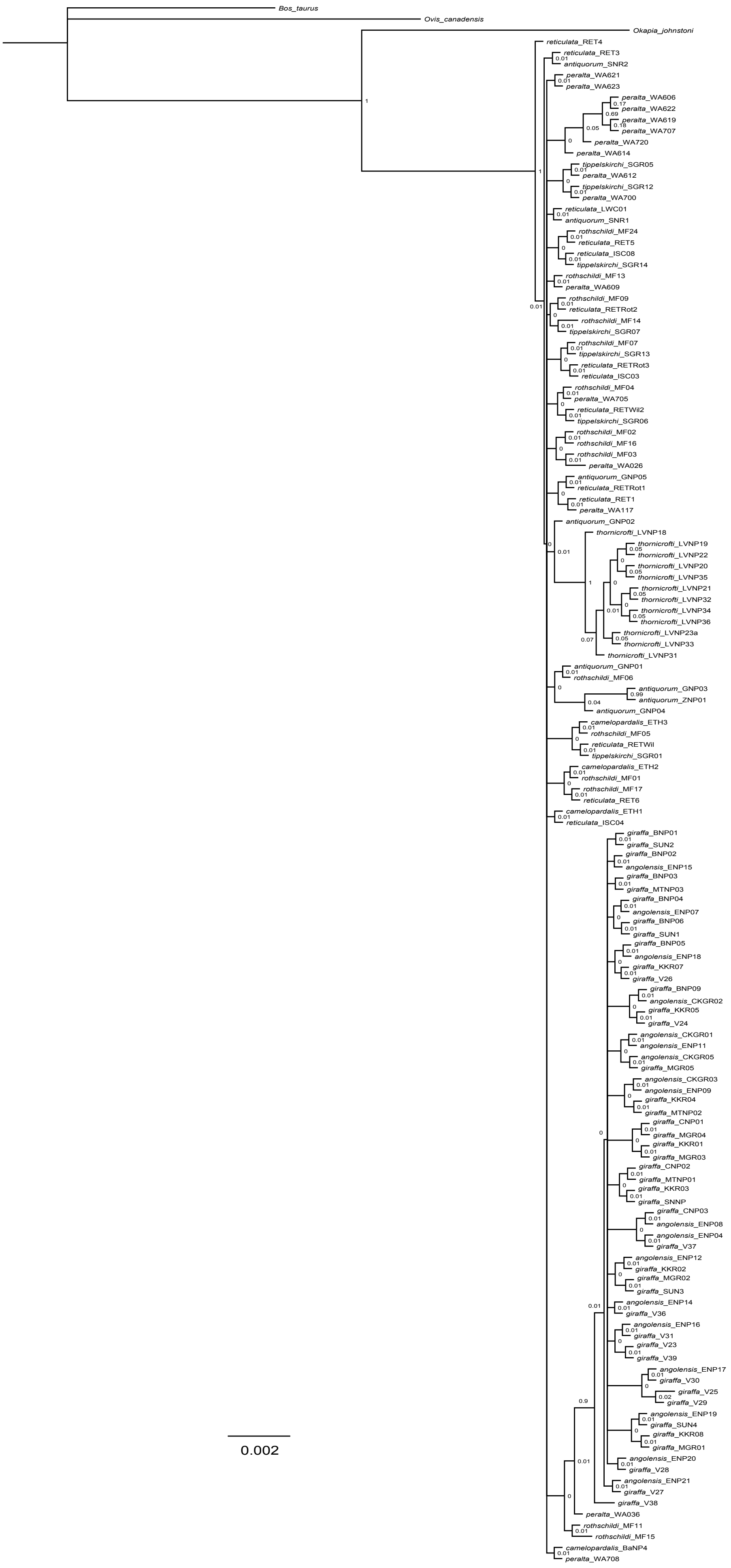

Fig L. Bayesian tree for the separate analysis of the 820 nucleotides (nt) long intron *IGF2B1* with the posterior probability values indicated for each node.

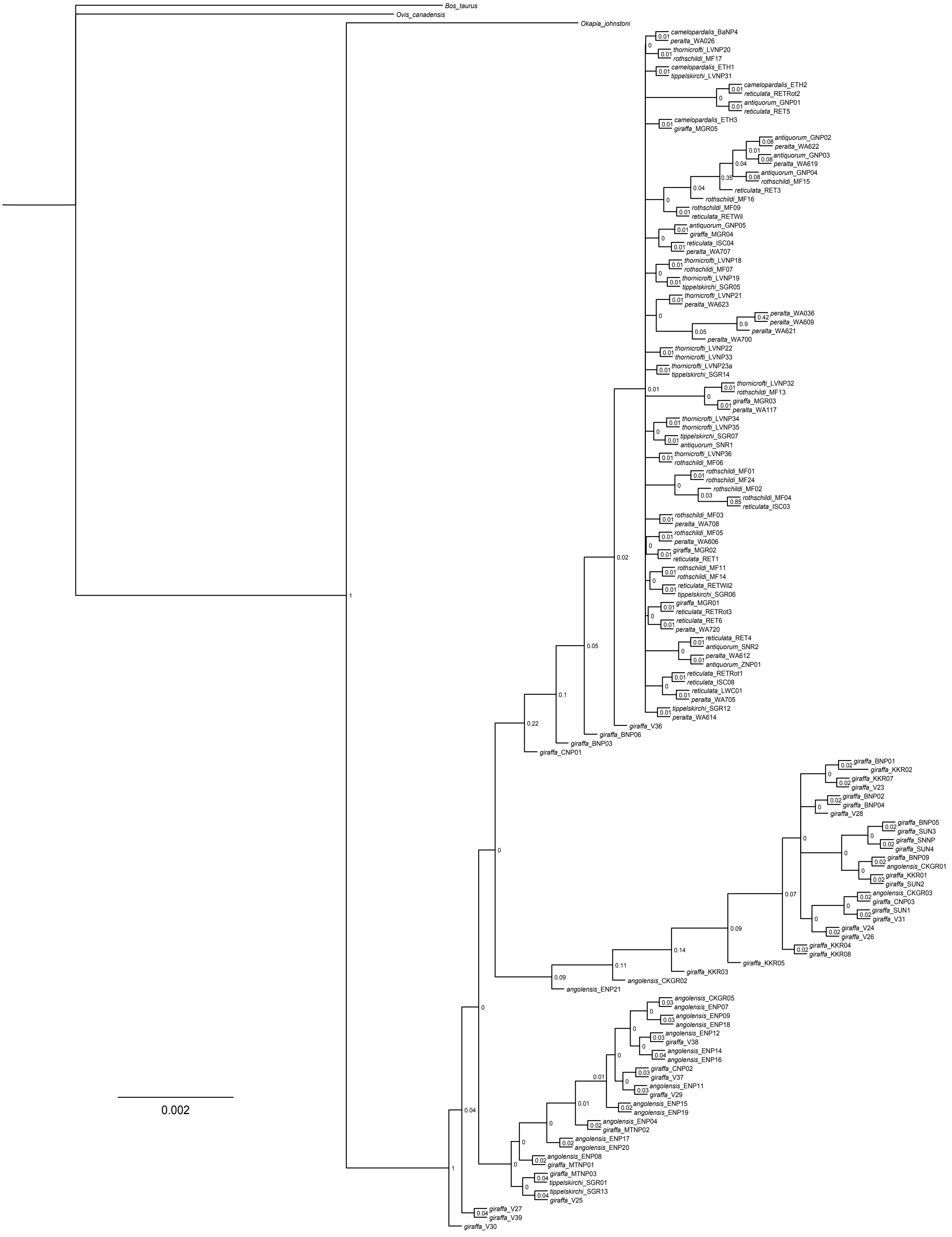

Fig M. Bayesian tree for the separate analysis of the 719 nucleotides (nt) long intron *MACF1* with the posterior probability values indicated for each node.

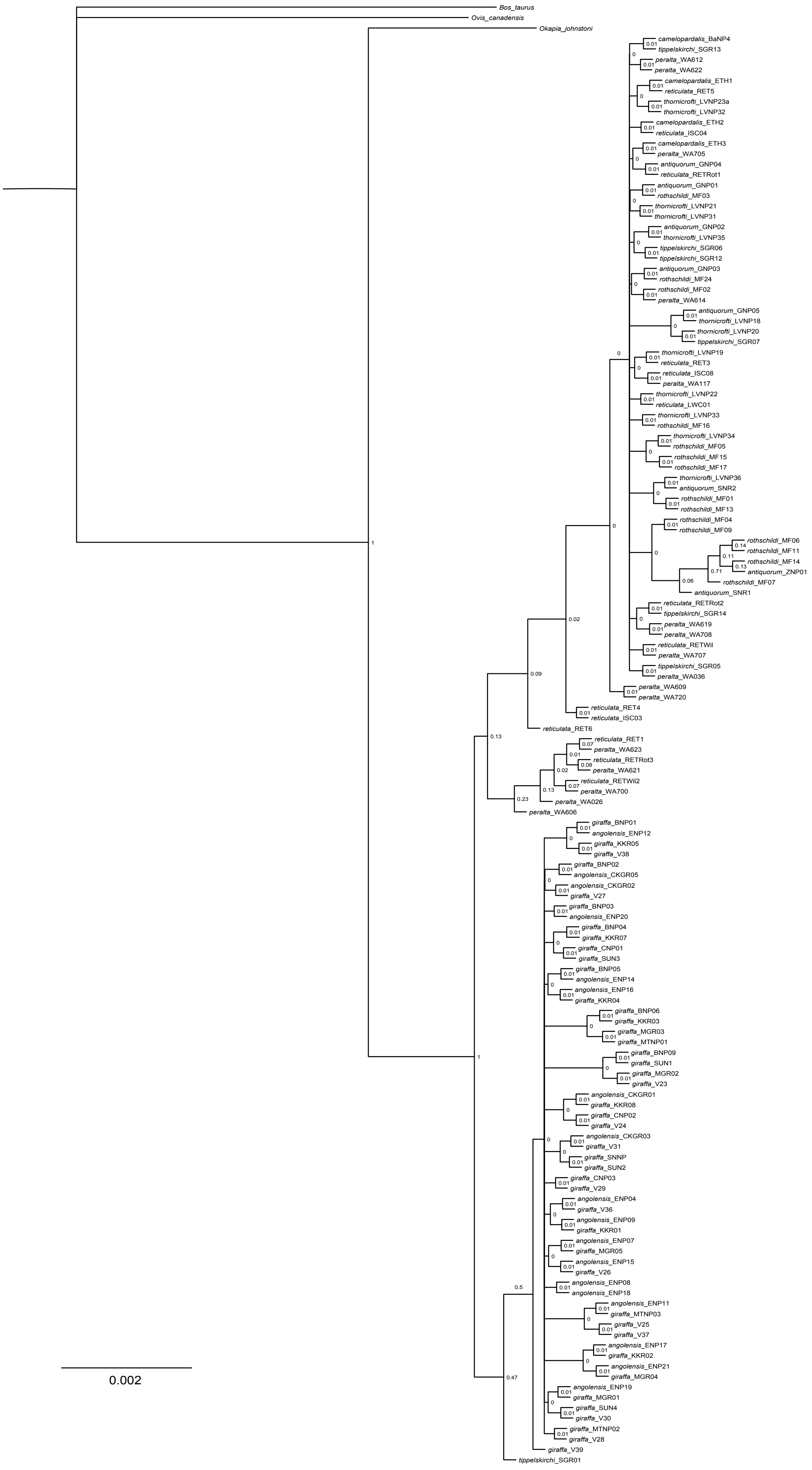

Fig N. Bayesian tree for the separate analysis of the 885 nucleotides (nt) long intron *NOTCH2* with the posterior probability values indicated for each node.

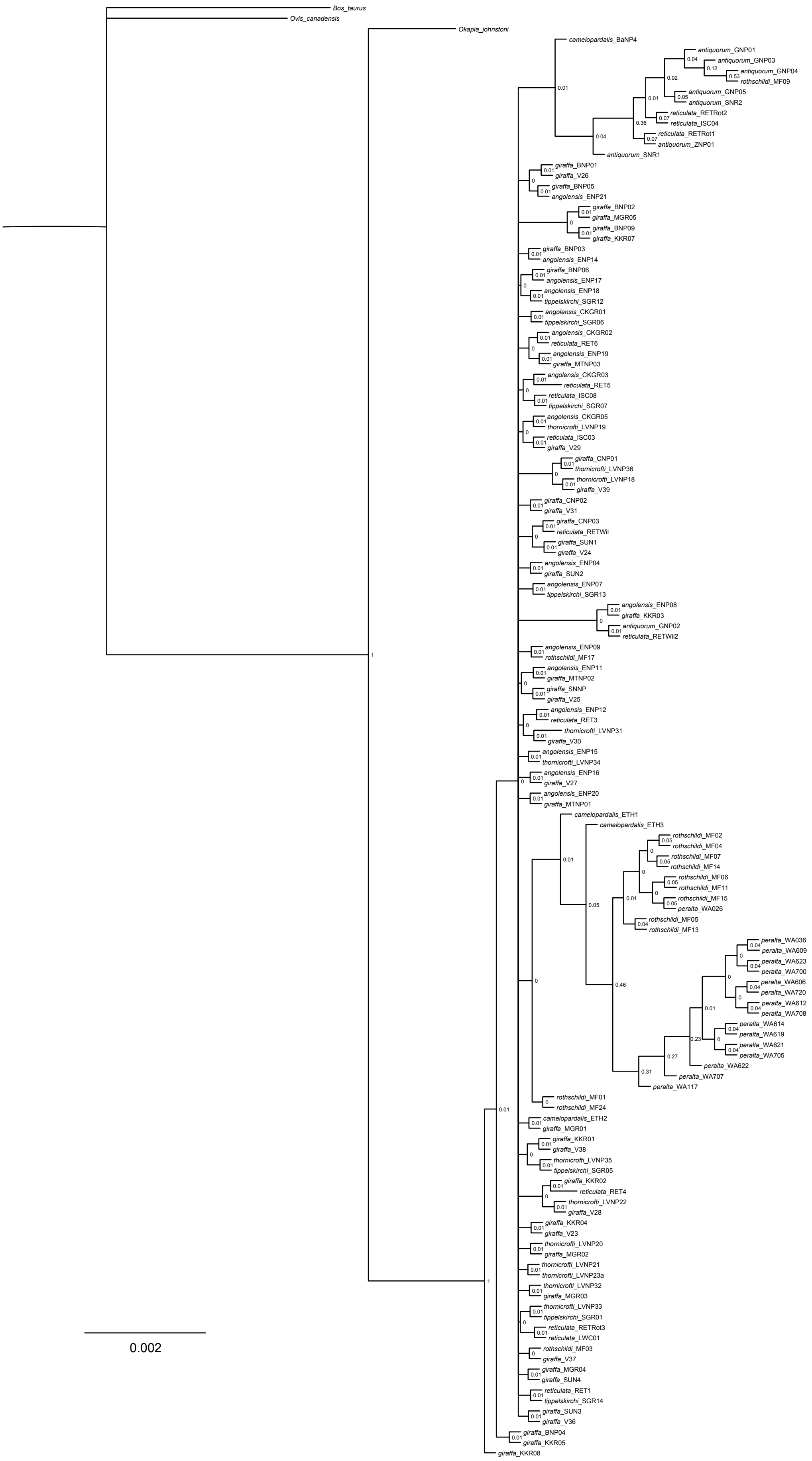

Fig O. Bayesian tree for the separate analysis of the 658 nucleotides (nt) long intron *NUP155* with the posterior probability values indicated for each node.

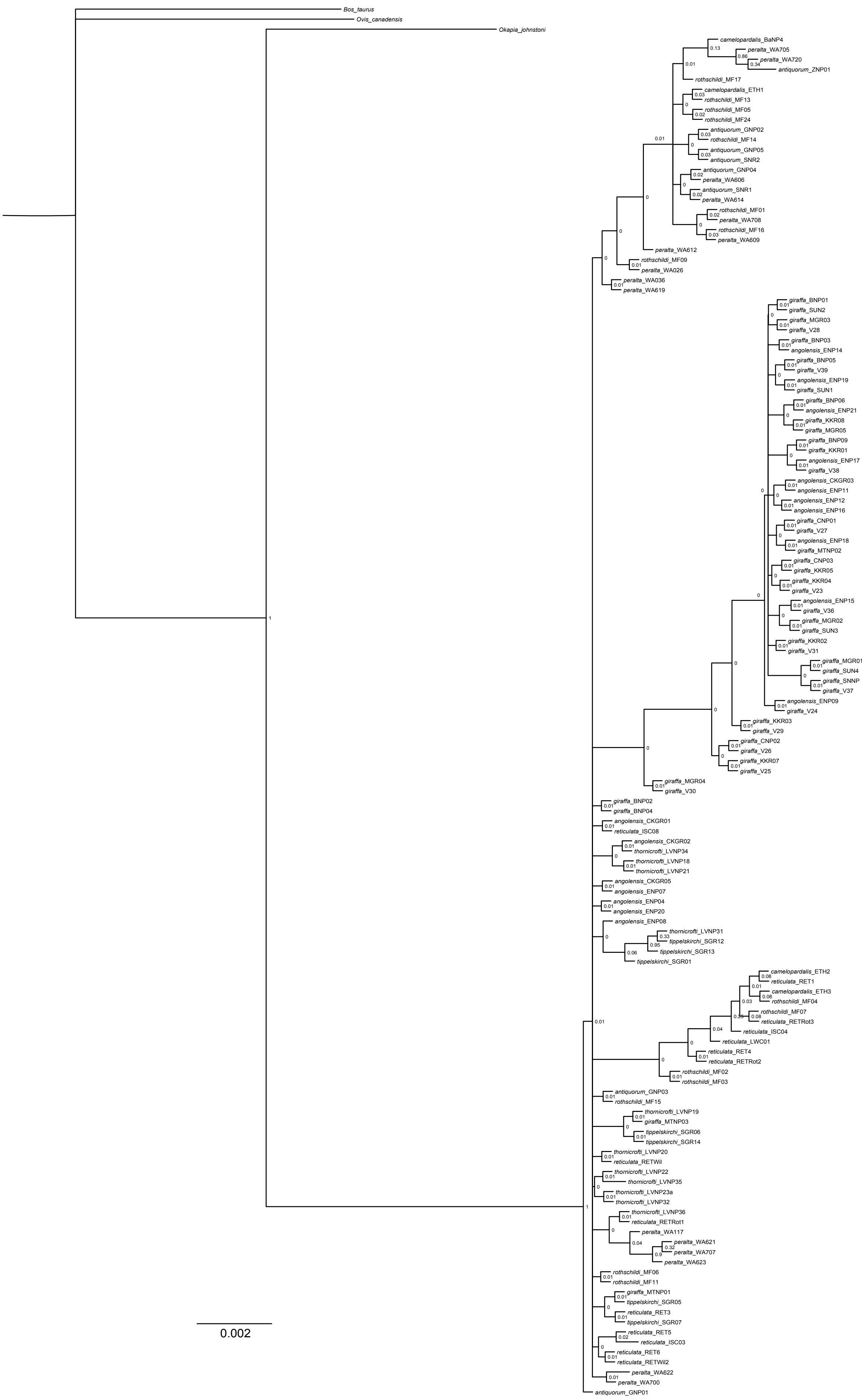

Fig P. Bayesian tree for the separate analysis of the 756 nucleotides (nt) long intron *OTOF* with the posterior probability values indicated for each node.

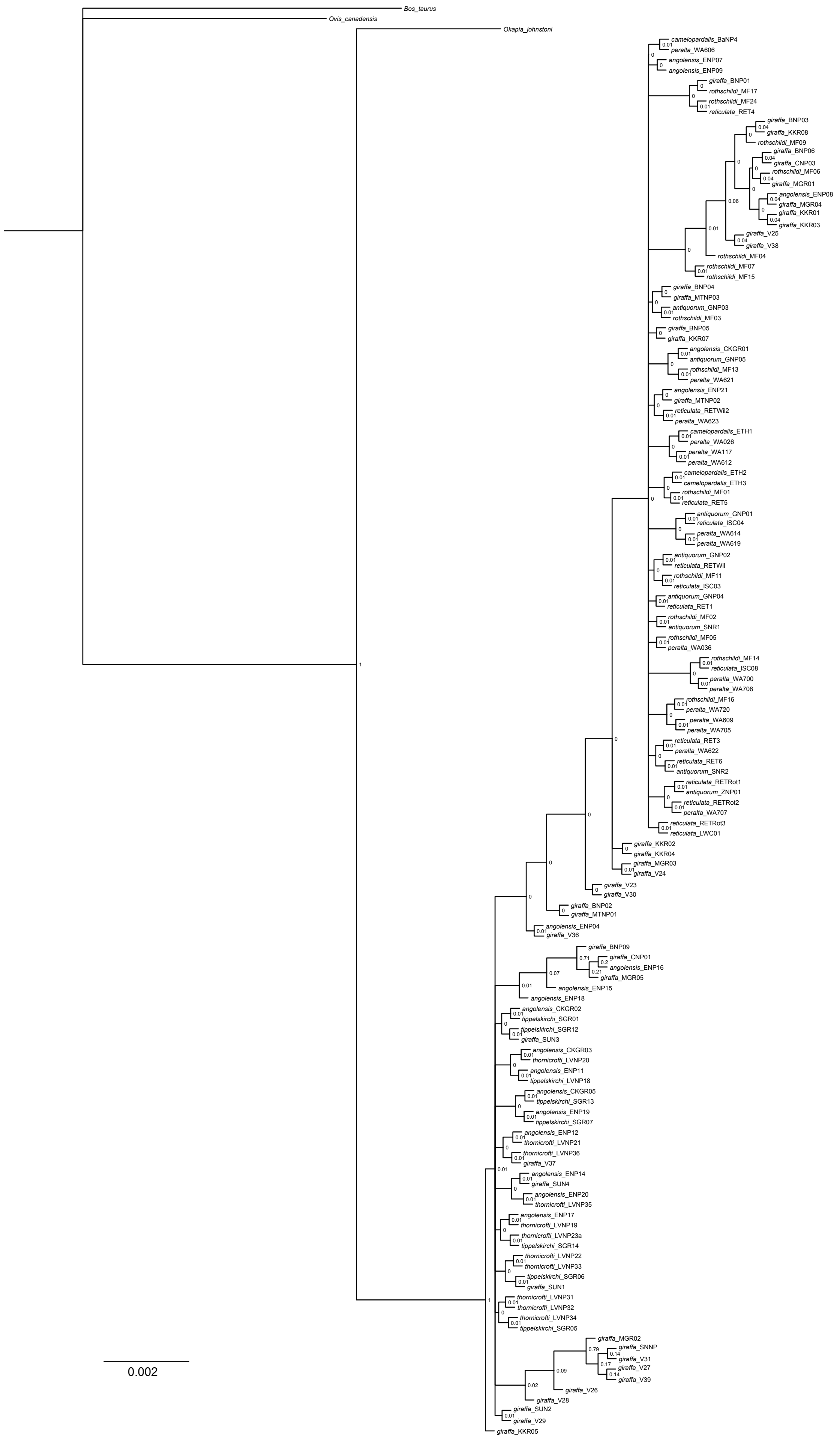

Fig Q. Bayesian tree for the separate analysis of the 897 nucleotides (nt) long intron *PLCE1* with the posterior probability values indicated for each node.

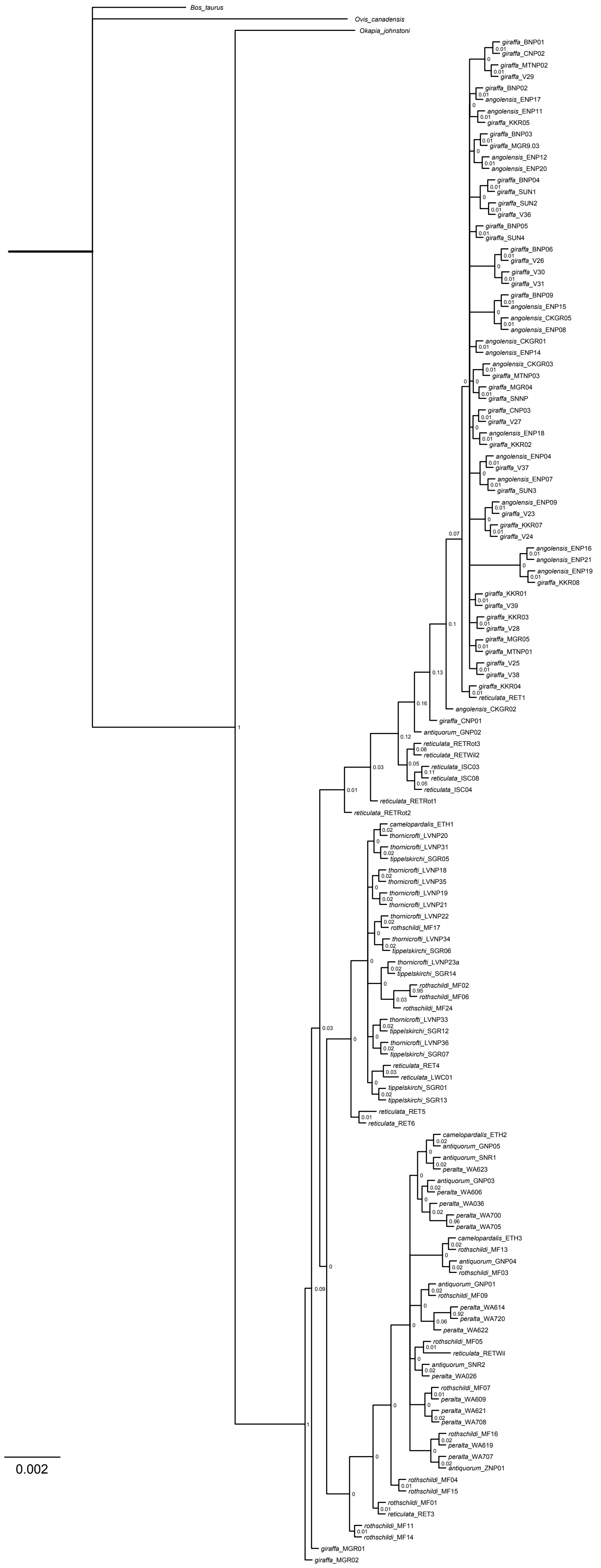

Fig R. Bayesian tree for the separate analysis of the 648 nucleotides (nt) long intron *RASSF4* with the posterior probability values indicated for each node.

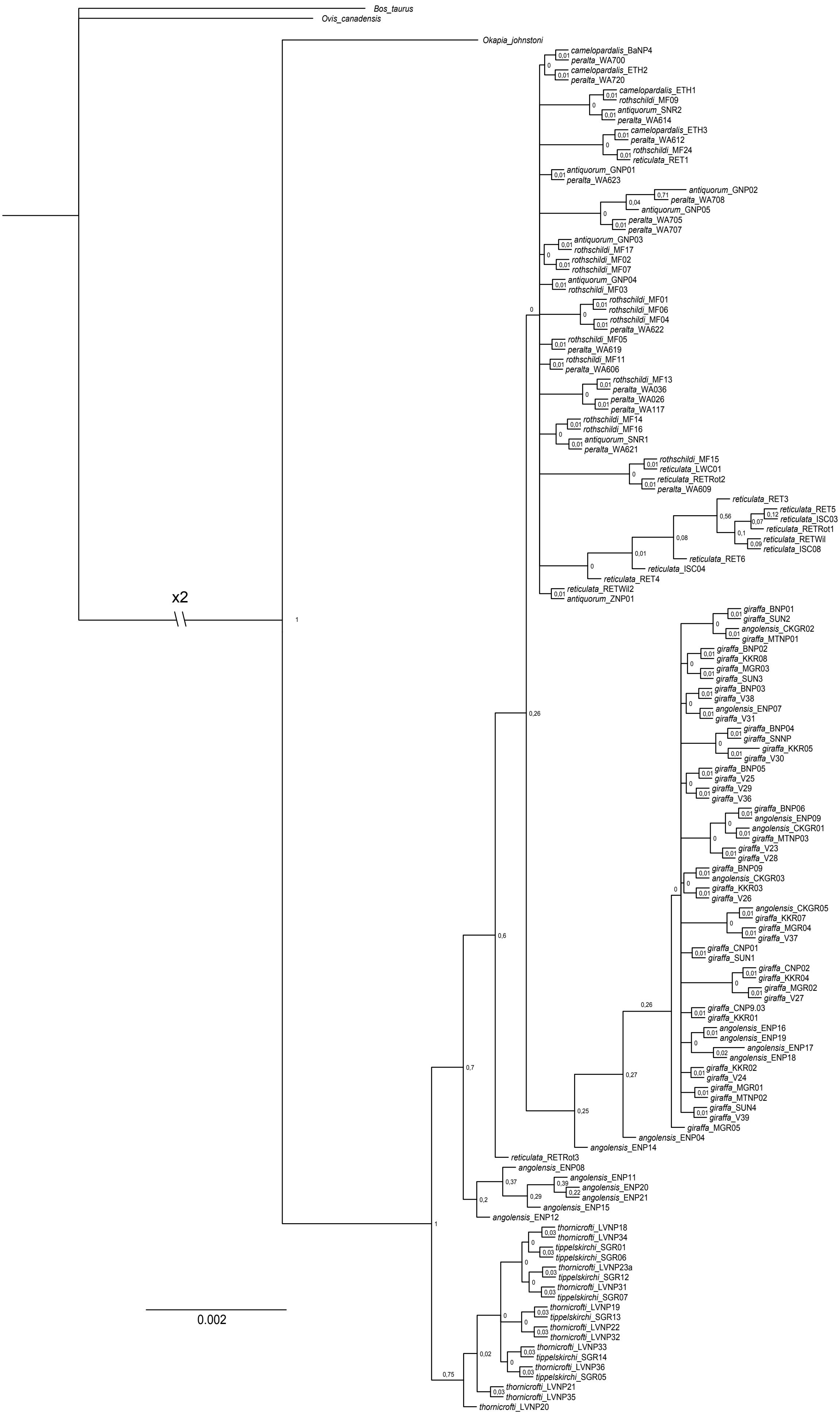

Fig S. Bayesian tree for the separate analysis of the 828 nucleotides (nt) long intron *RFC5* with the posterior probability values indicated for each node.

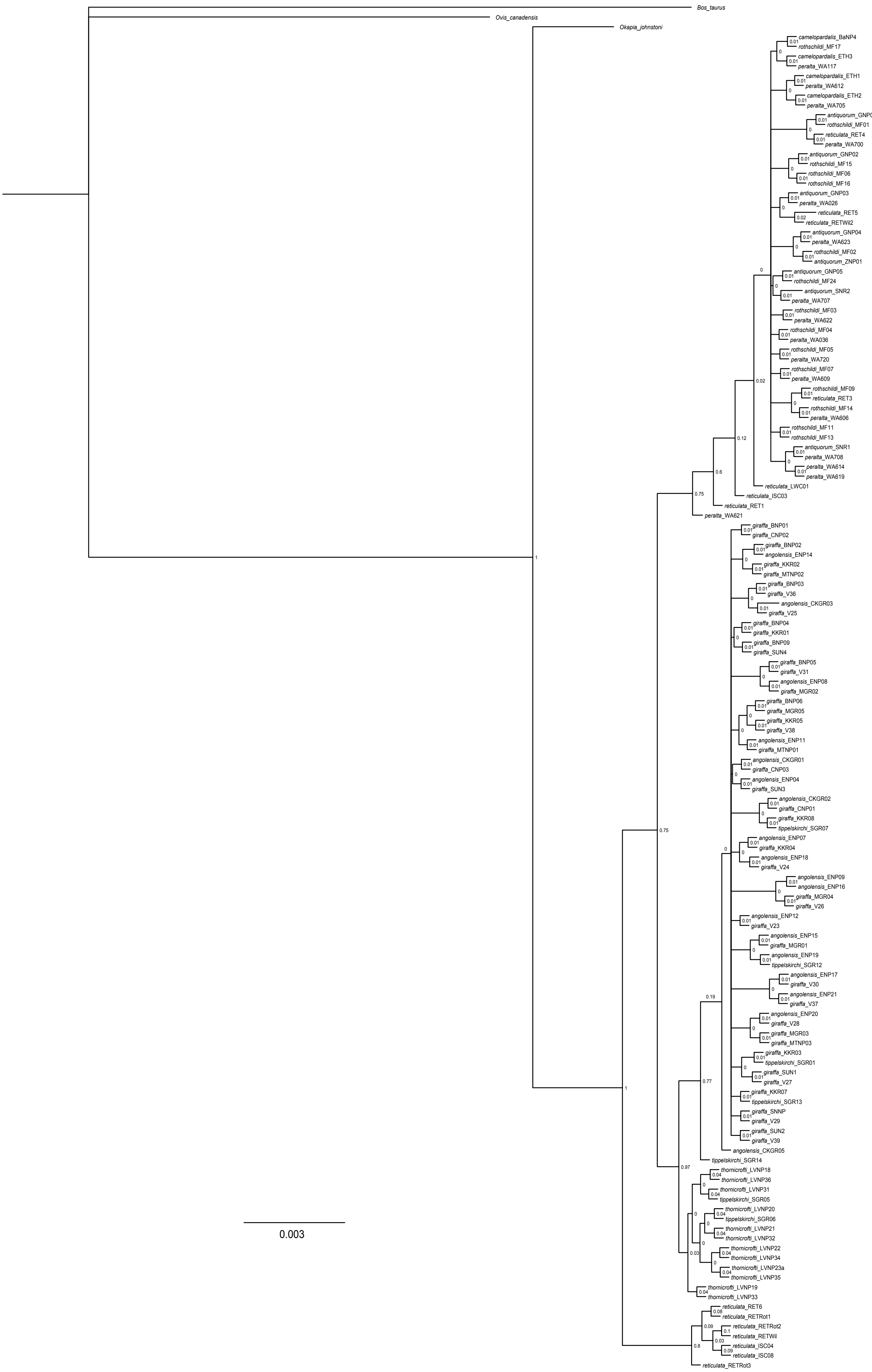

Fig T. Bayesian tree for the separate analysis of the 933 nucleotides (nt) long intron *SAP130* with the posterior probability values indicated for each node.

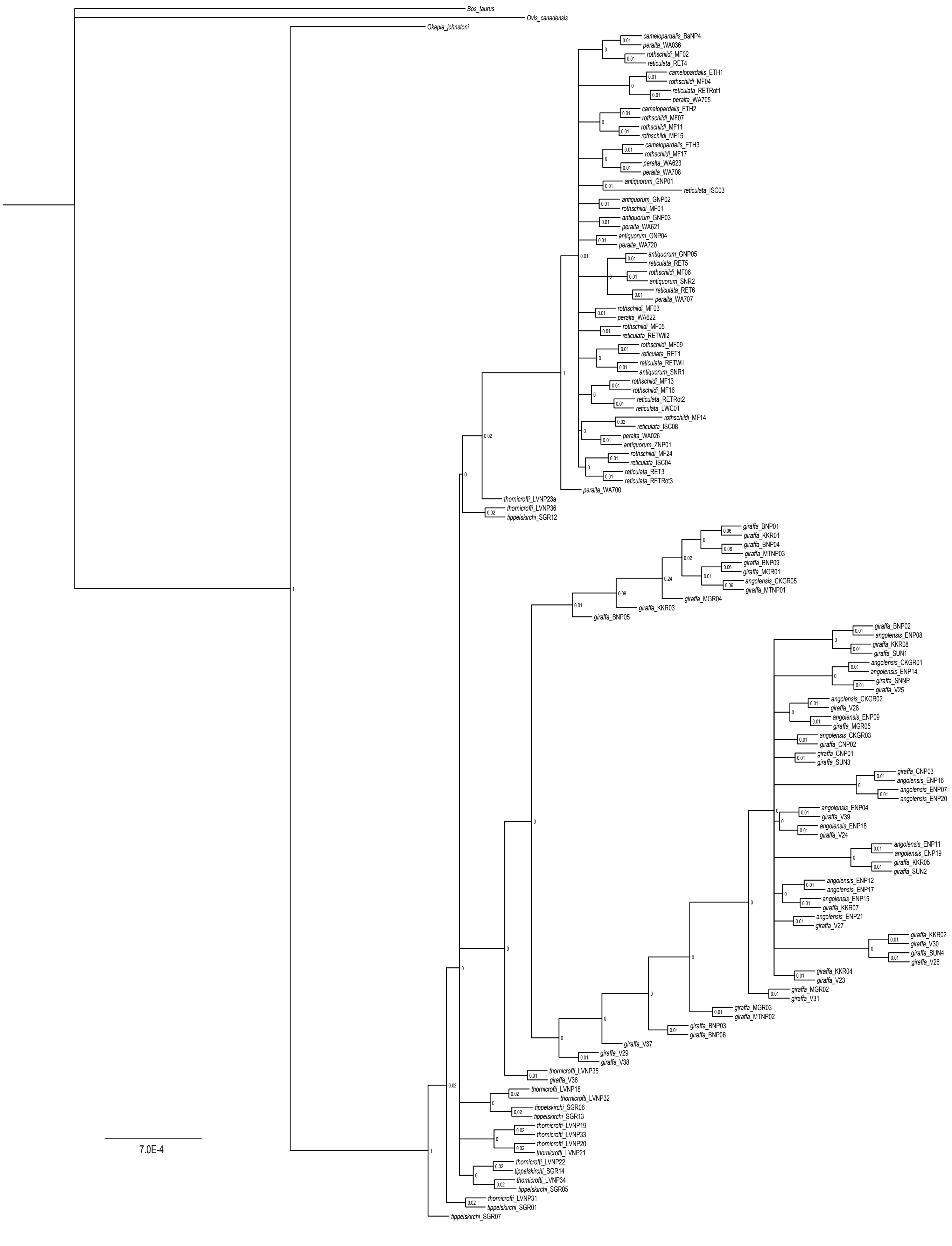

Fig U. Bayesian tree for the separate analysis of the 765 nucleotides (nt) long intron SOS1 with the posterior probability values indicated for each node.

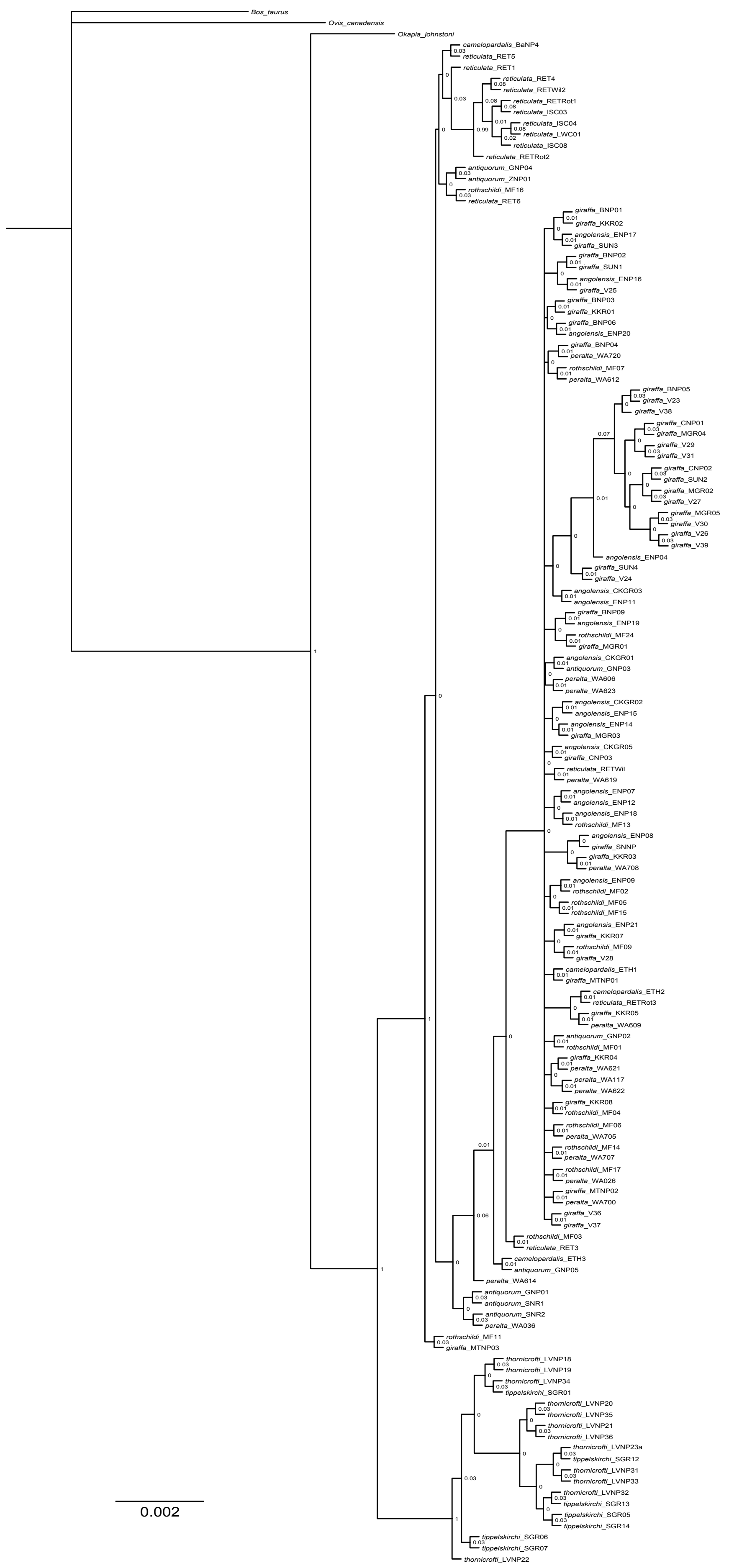

Fig V. Bayesian tree for the separate analysis of the 724 nucleotides (nt) long intron *UBN2* with the posterior probability values indicated for each node.

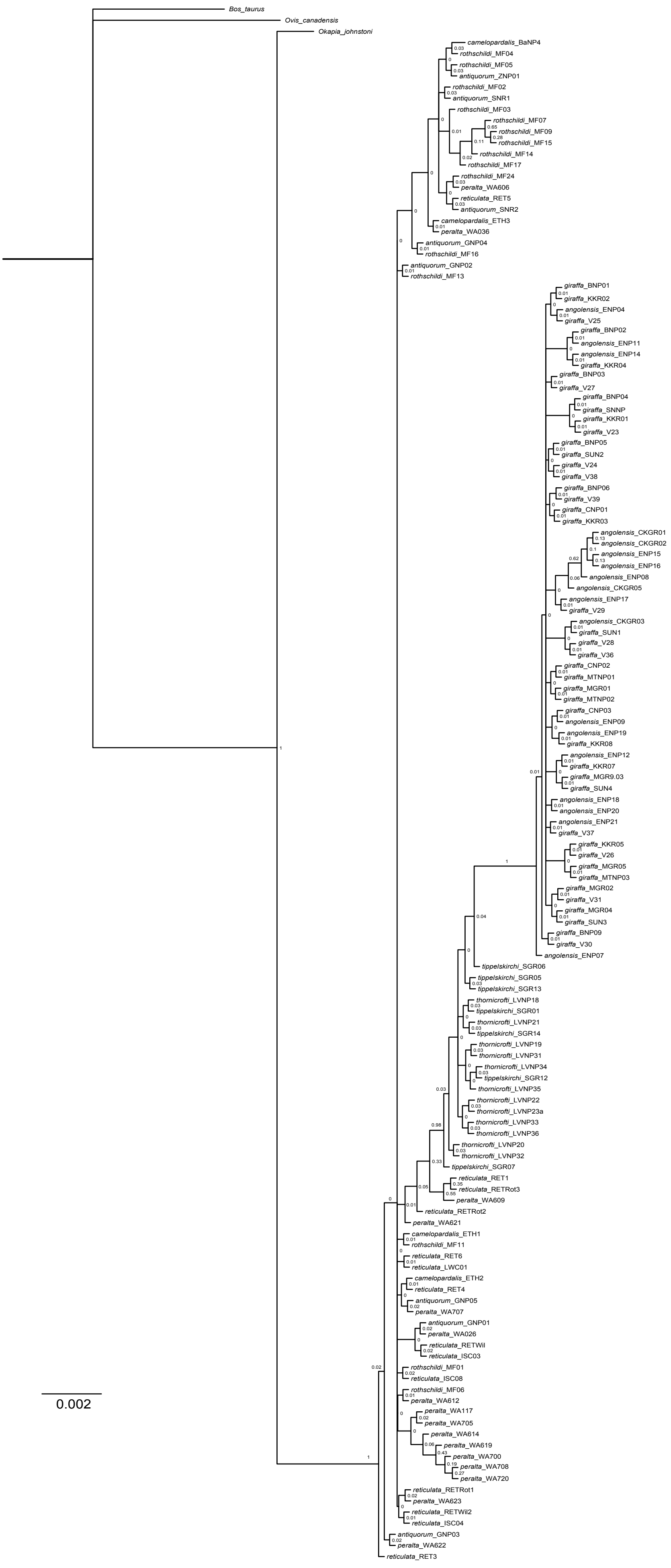

Fig W. Bayesian tree for the separate analysis of the 940 nucleotides (nt) long intron *USP33* with the posterior probability values indicated for each node.

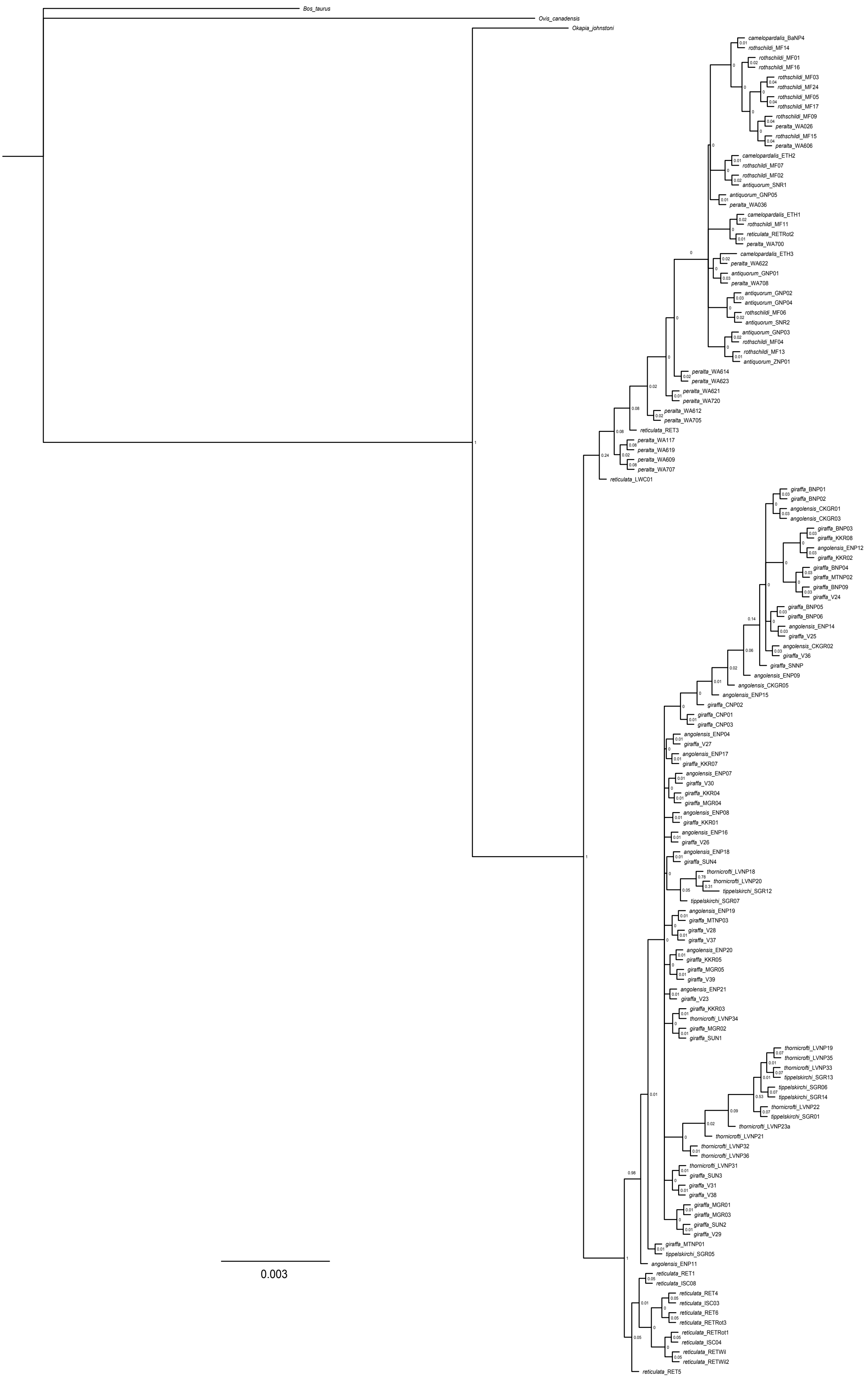

Fig X. Bayesian tree for the separate analysis of the 1332 nucleotides (nt) long intron *USP54* with the posterior probability values indicated for each node.

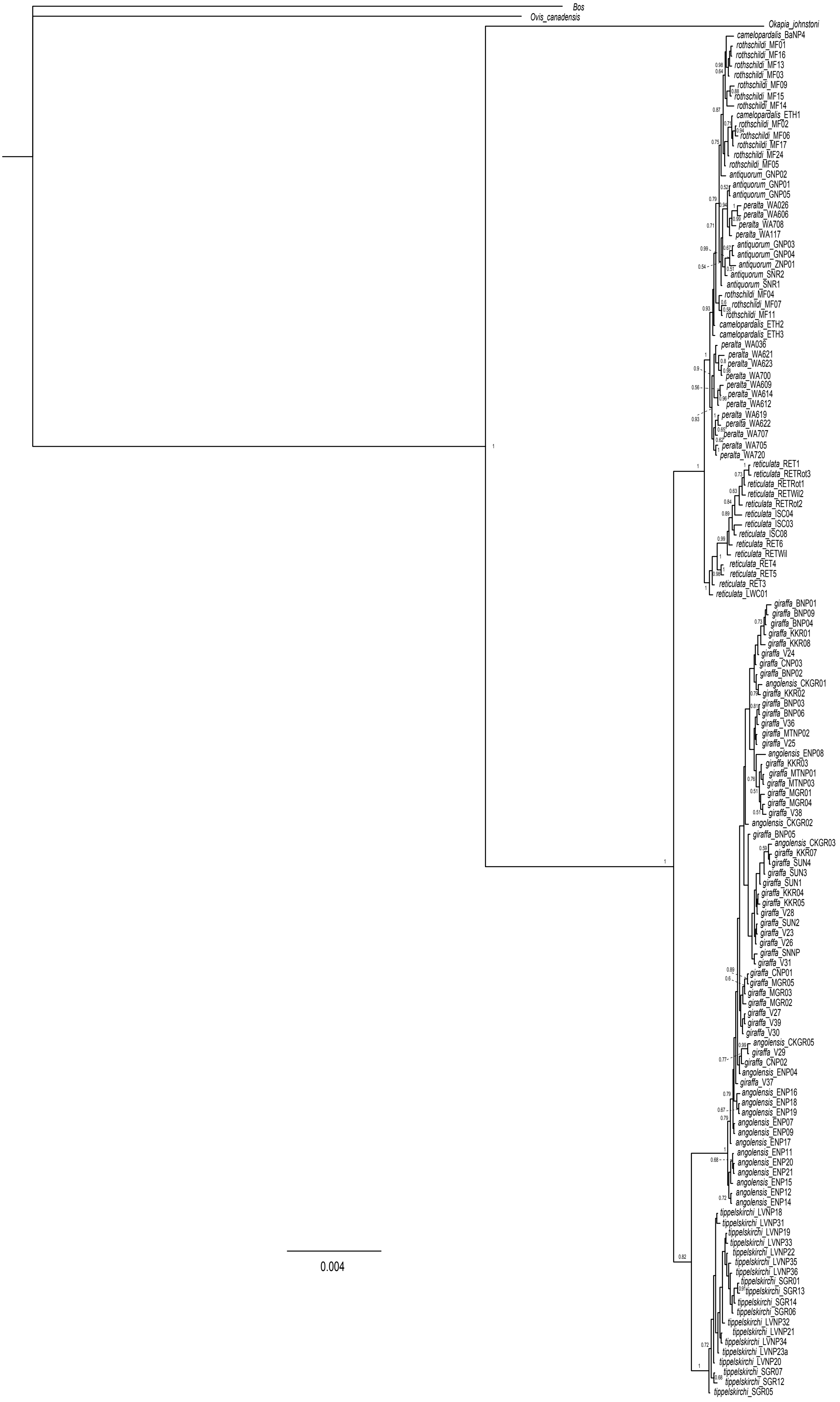

Fig Y. Bayesian tree (PP > 0.5) obtained from the analyses of nuDNA-137O3 dataset.

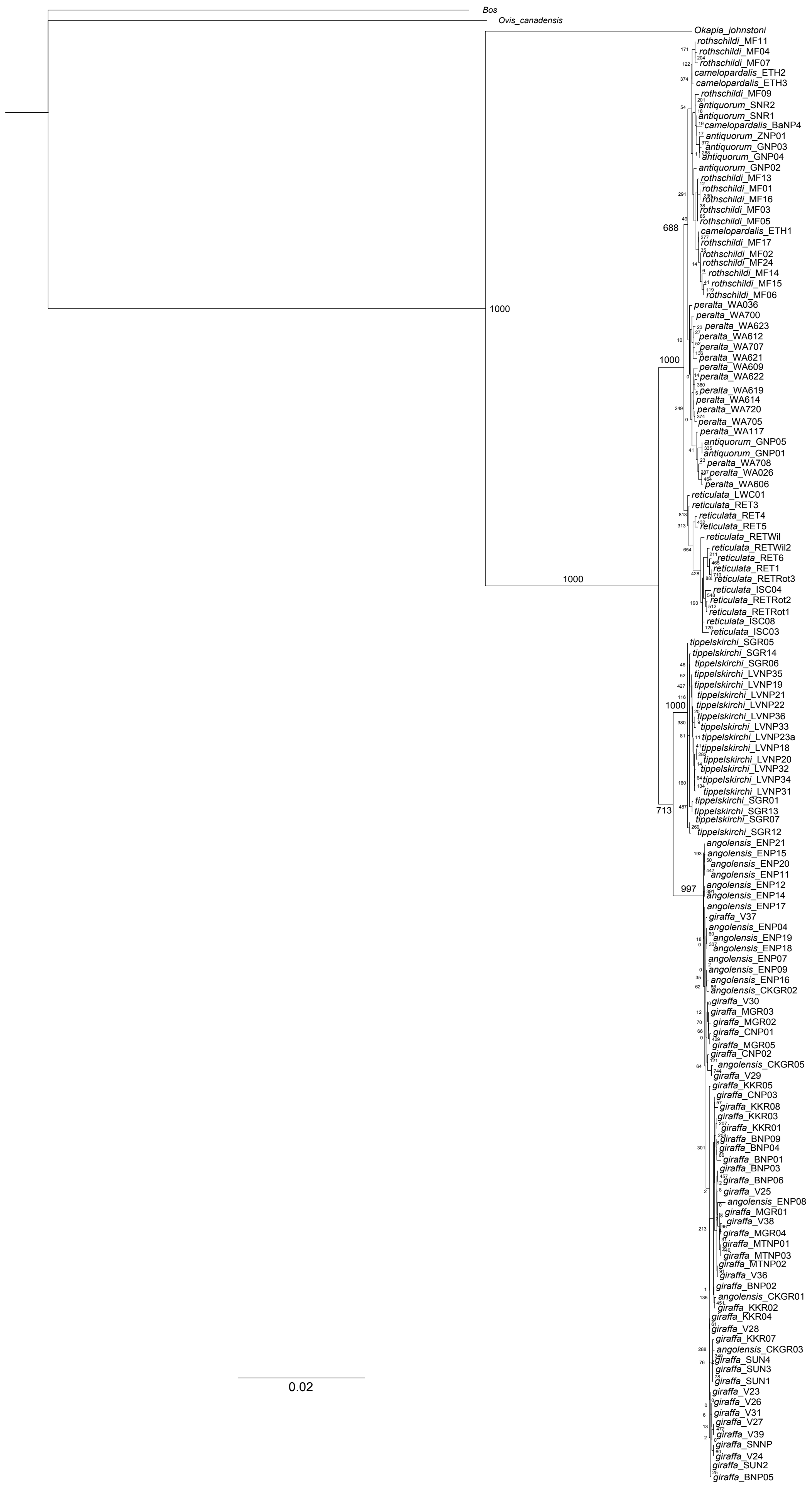

Fig Z. PhyML tree from the Maximum Likelihood analyses of the nuDNA-137O3 dataset.

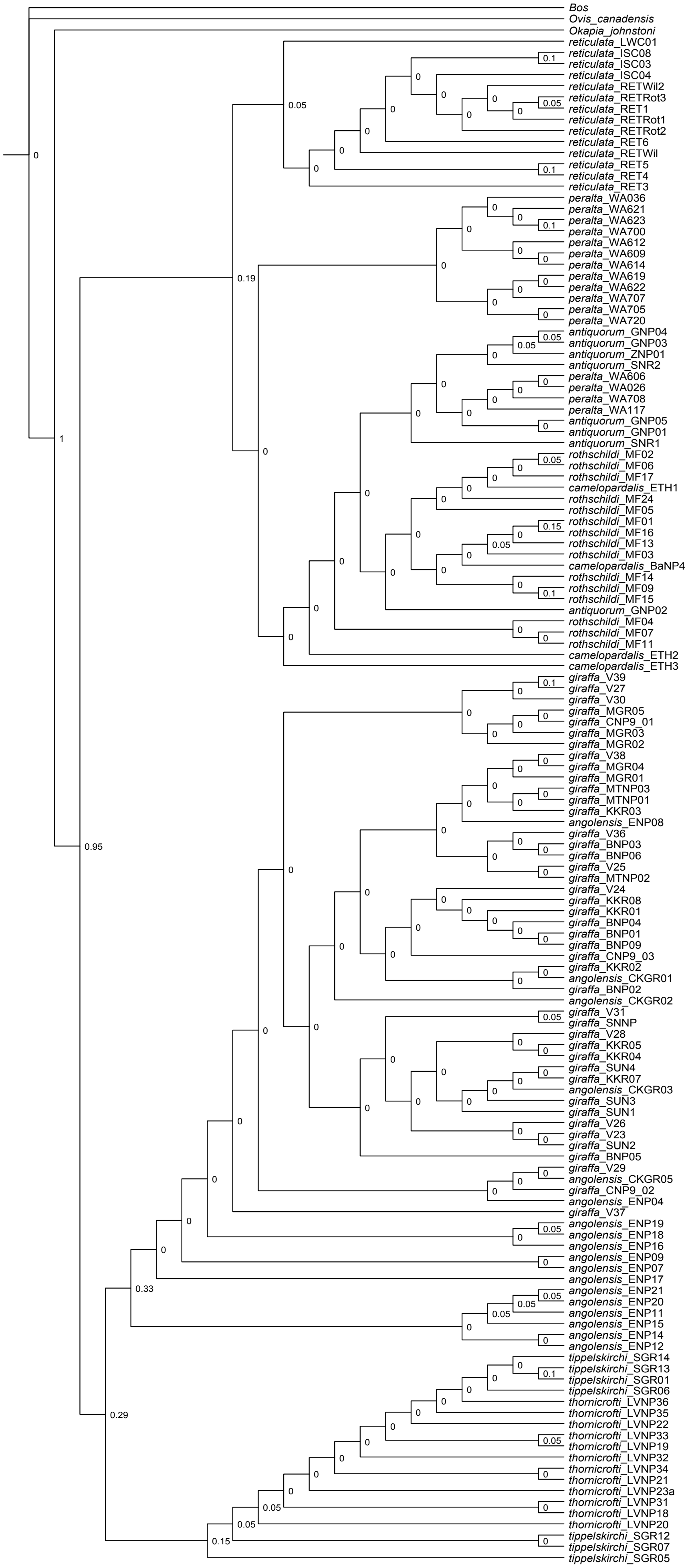

Fig Aa. Supertree showing the reproducibility indices (Rep) for the given topology, obtained from the analyses of the MRP matrix including 21 nuclear markers.

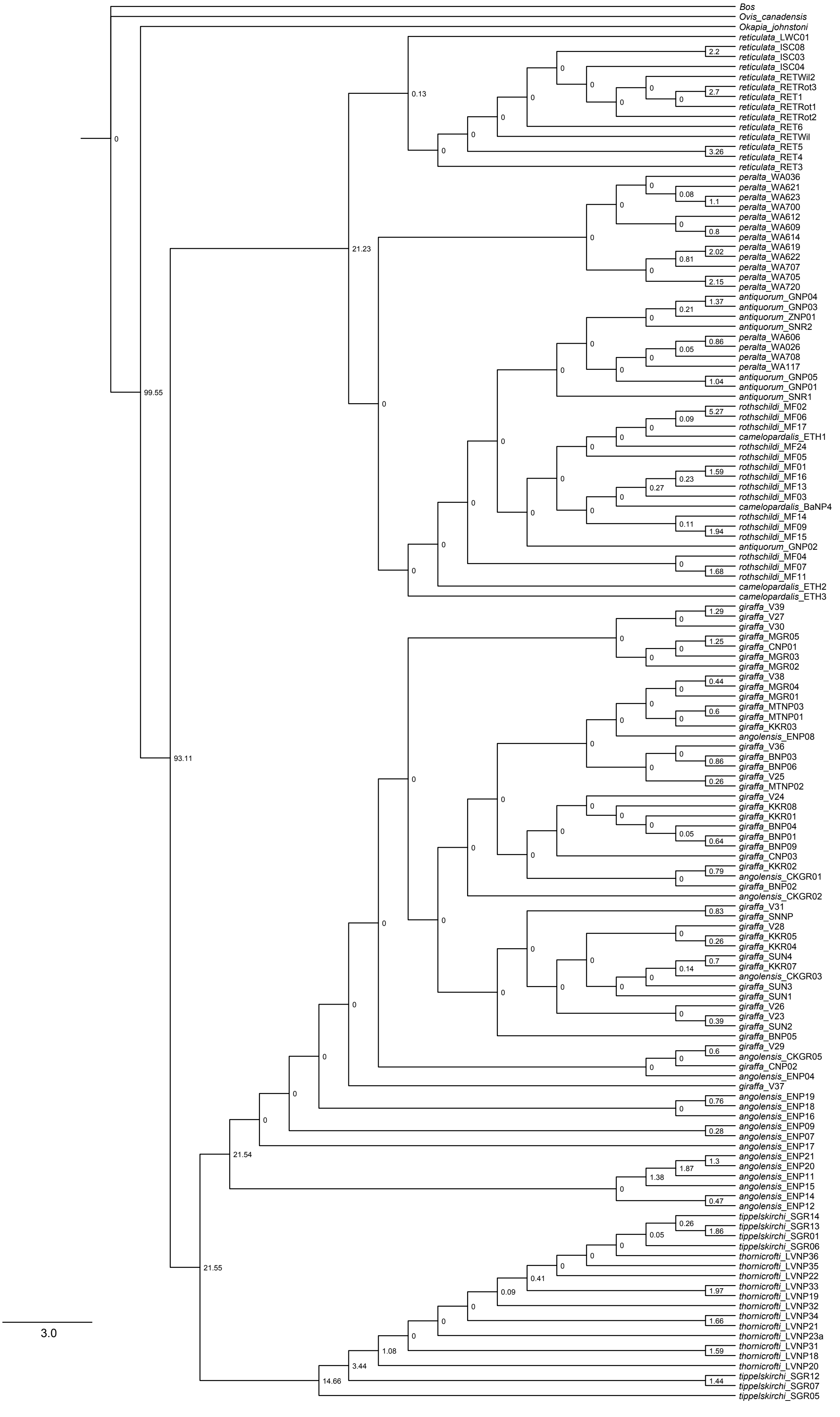

Fig Ab. Supertree showing the mean posterior probability values (MPP) for the given topology, obtained from the analyses of the MRP matrix including 21 nuclear markers.
