# Supplementary material for: A comparative approach for species delimitation based on multiple methods of multi-locus DNA sequence analysis: A case study of the genus Giraffa (Mammalia, Cetartiodactyla)
Source: PLoS One. 2020 Feb 13;15(2):e0217956. doi: 10.1371/journal.pone.0217956 (PMC7018015; doi:10.1371/journal.pone.0217956)
Supplement: S3 Appendix — (PDF) [file pone.0217956.s003.pdf]

S3 Appendix. Population Structure analyses

A comparative approach for species delimitation based on multiple methods of multi-locus DNA sequence analysis: a case study of the genus *Giraffa* (Mammalia, Cetartiodactyla)

Alice Petzold[1,2], Alexandre Hassanin[1,2]*

1 Institut de Systématique, Évolution, Biodiversité (ISYEB), Sorbonne Université, MNHN, CNRS, EPHE, Paris, France

2 Muséum national d'Histoire naturelle, CP51, 55 rue Buffon - 75005 Paris, France

*Correspondence: alexandre.hassanin@mnhn.fr

STRUCTURE HARVESTER results for K = 1-10 populations.

The log probabilities of the data (ln Pr (X|K) and ΔK were obtained from 24 STRUCTURE analyses based on 21 nuclear introns for 137 giraffe individuals (see Table 2) using different model parameters (see below for details, summarized in Table 3).

Fig A. Admixture model, LOCPRIOR, correlated allele frequency, λ = 1.0

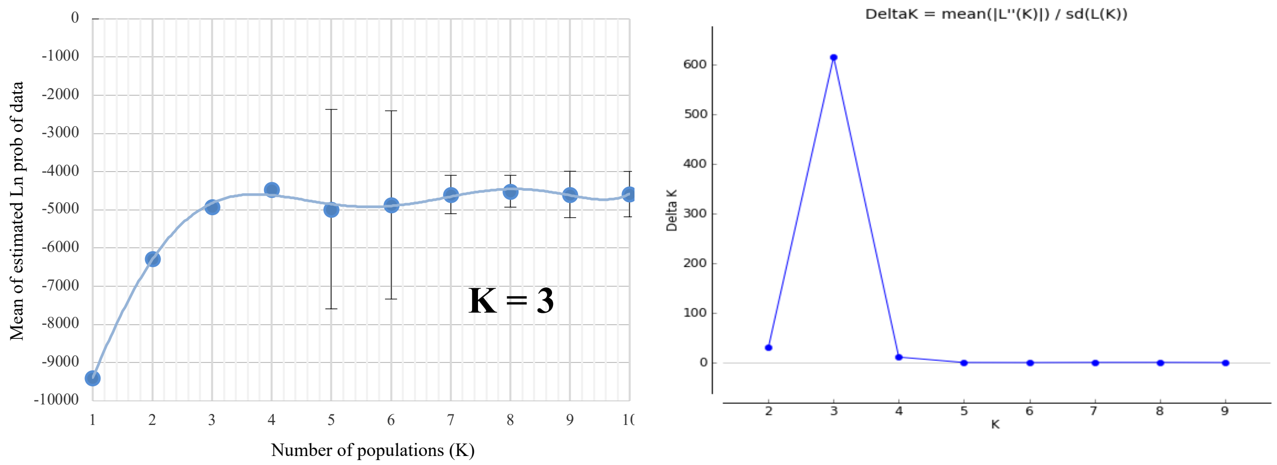

Fig B. Admixture model, LOCPRIOR, independent allele frequency, λ = 1.0

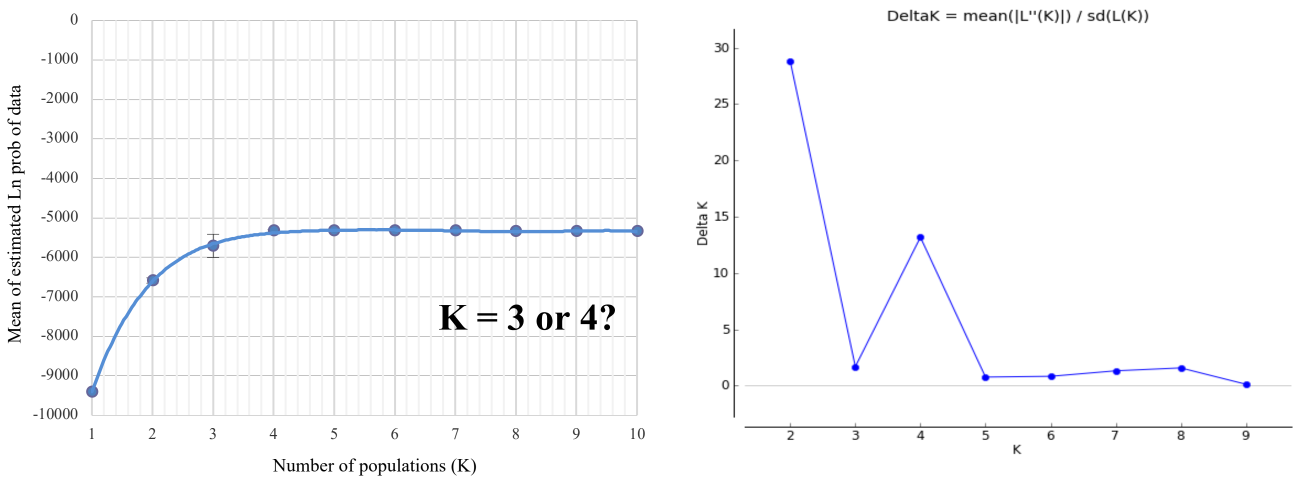

Fig C. Admixture model, POPID, correlated allele frequency, $\lambda = 1.0$

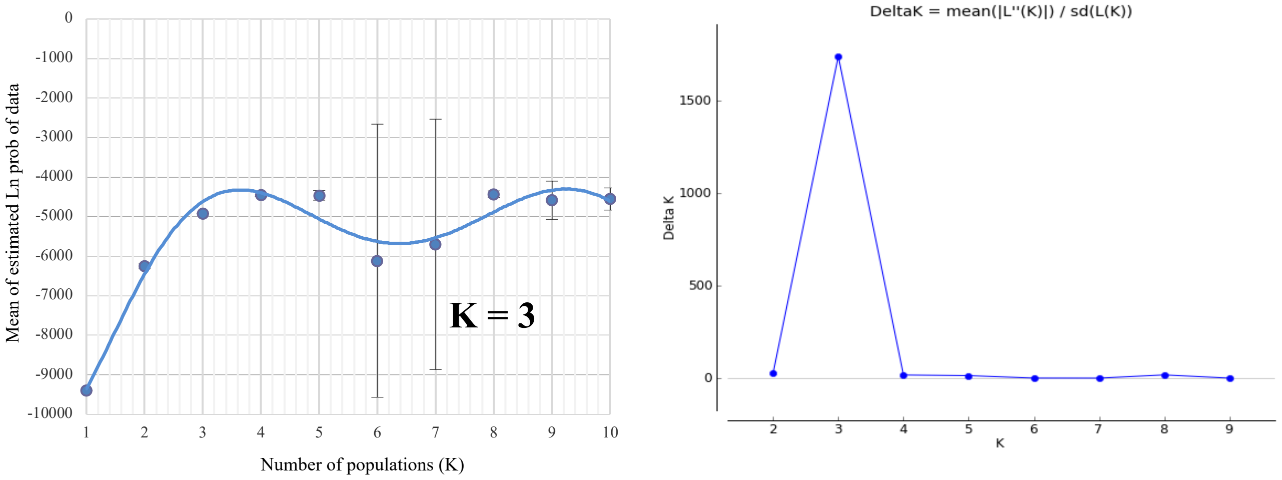

Fig D. Admixture model, POPID, independent allele frequency, $\lambda = 1.0$

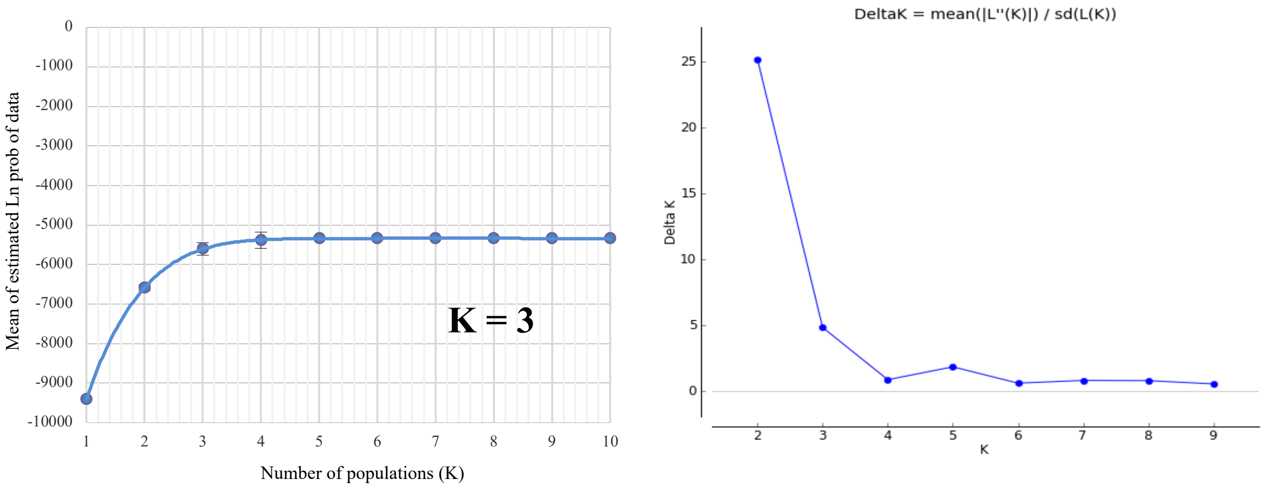

Fig E. Admixture model, no assignation, correlated allele frequency, $\lambda = 1.0$

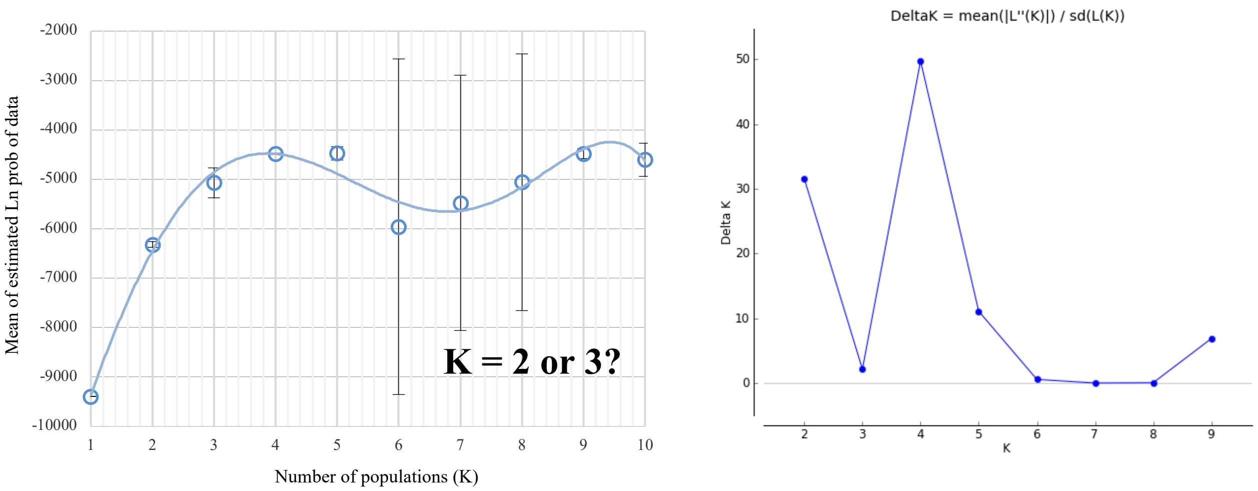

Fig F. Admixture model, no assignation, independent allele frequency, λ = 1.0

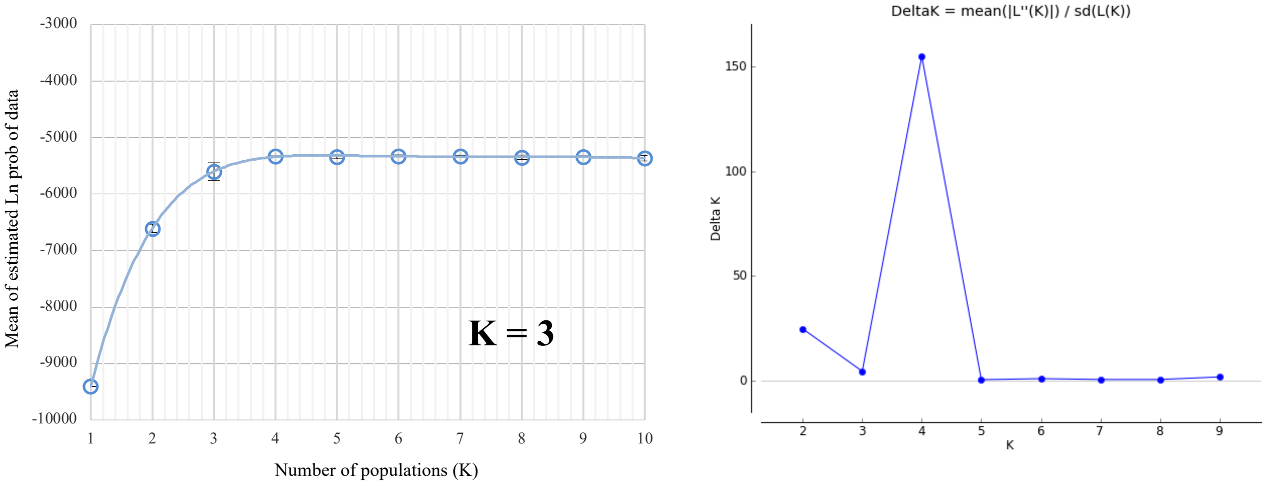

Fig G. No admixture model, LOCPRIOR, correlated allele frequency, λ = 1.0

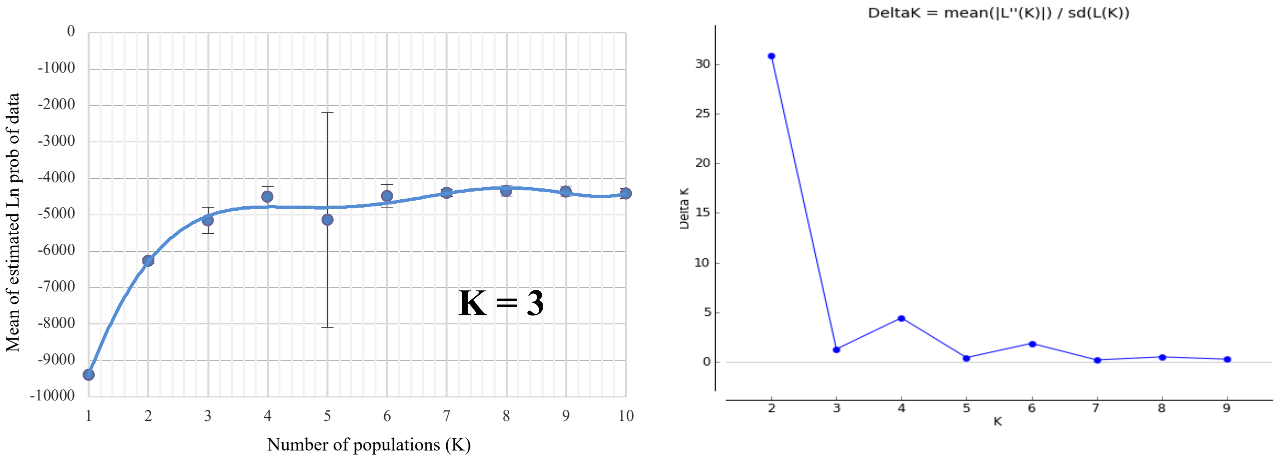

Fig H. No admixture model, LOCPRIOR, independent allele frequency, λ = 1.0

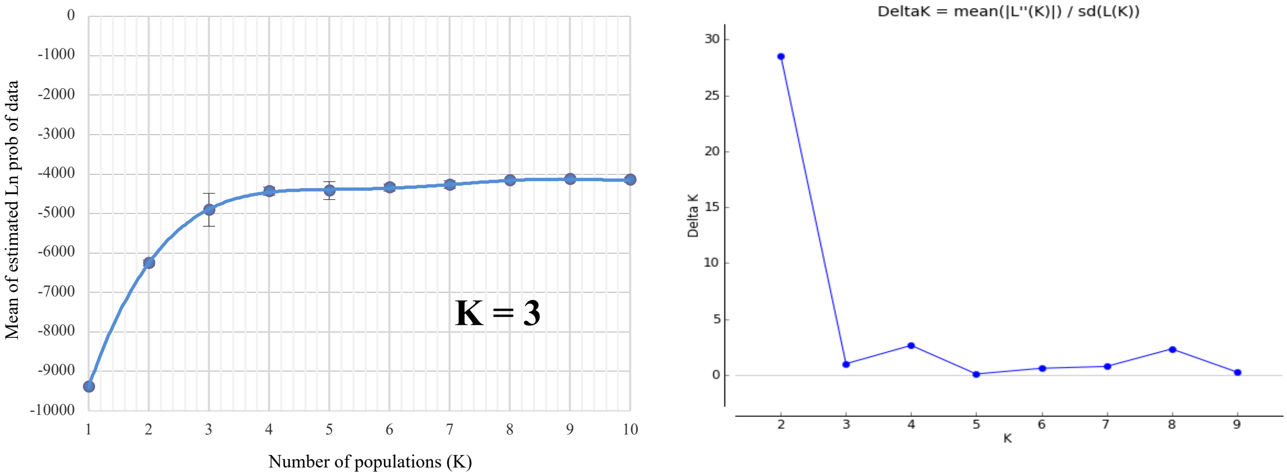

Fig I. No admixture model, POPID, correlated allele frequency, λ = 1.0

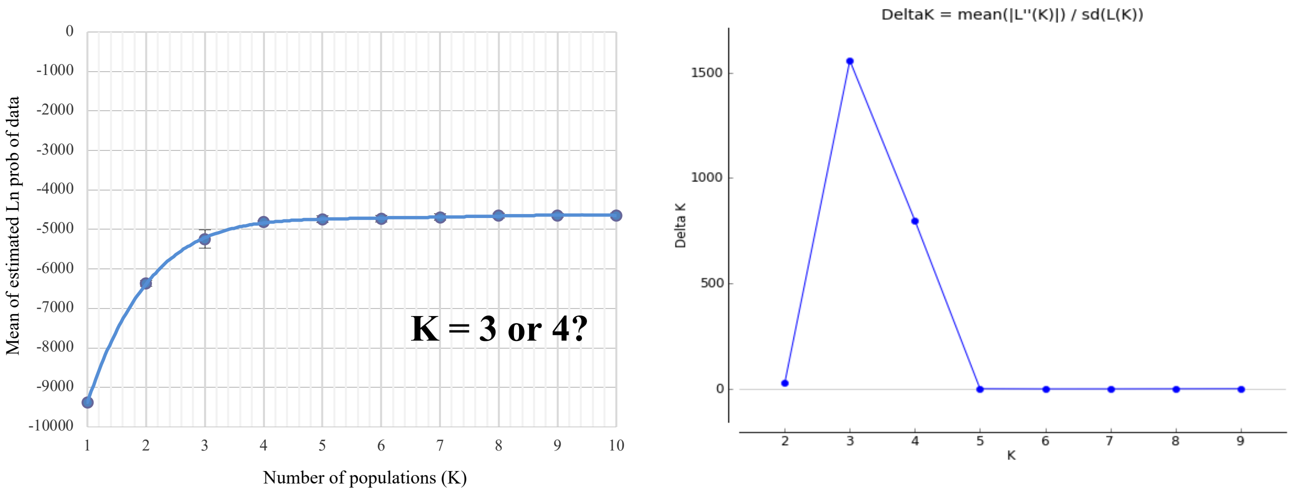

Fig J. No admixture model, POPID, independent allele frequency, λ = 1.0

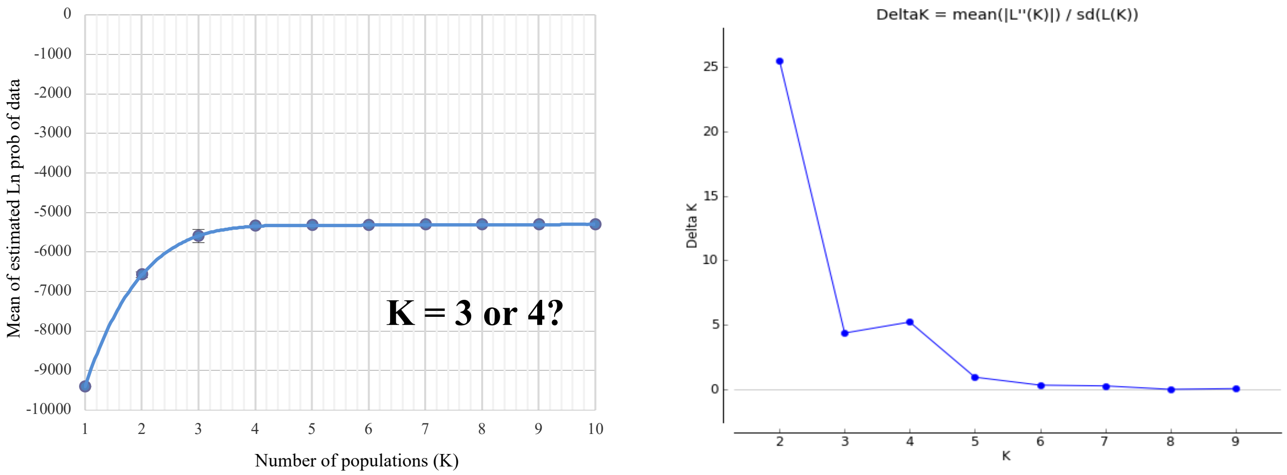

Fig K. No admixture model, no assignation, correlated allele frequency, λ = 1.0

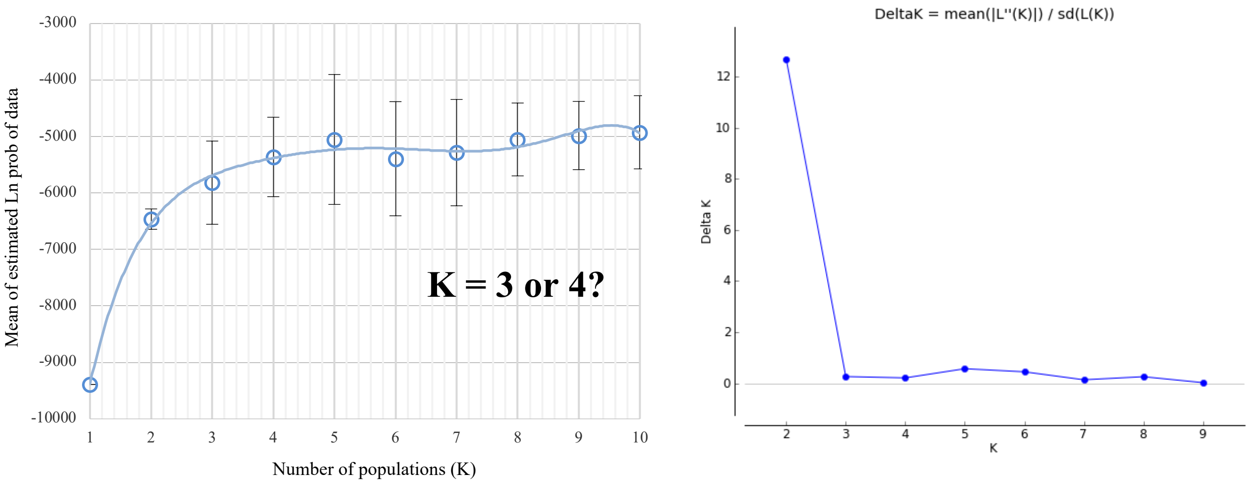

Fig L. No admixture model, no assignation, independent allele frequency, λ = 1.0

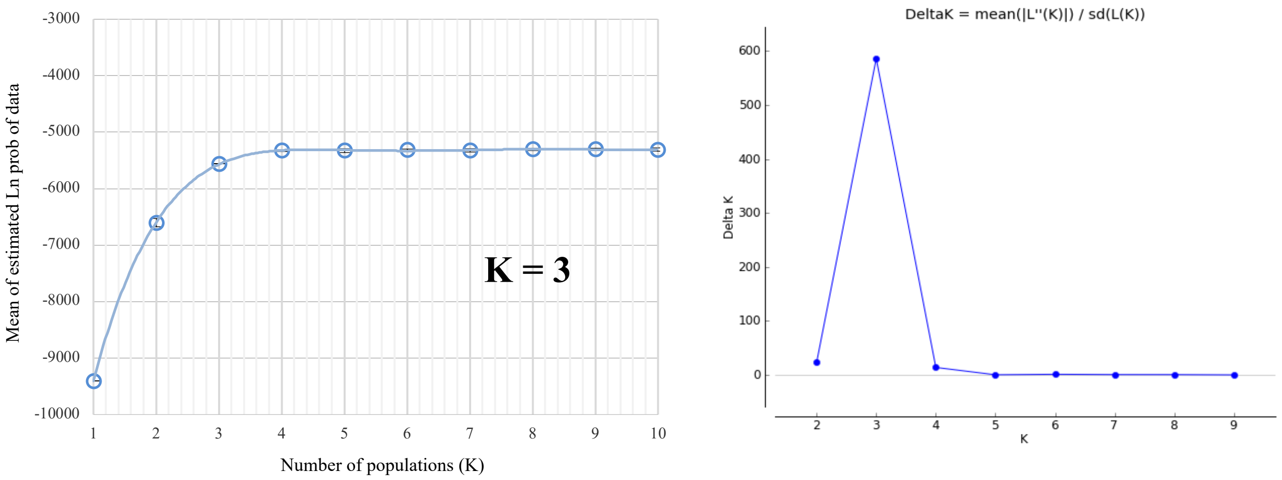

Fig M. Admixture model, LOCPRIOR, correlated allele frequency, λ = 0.45

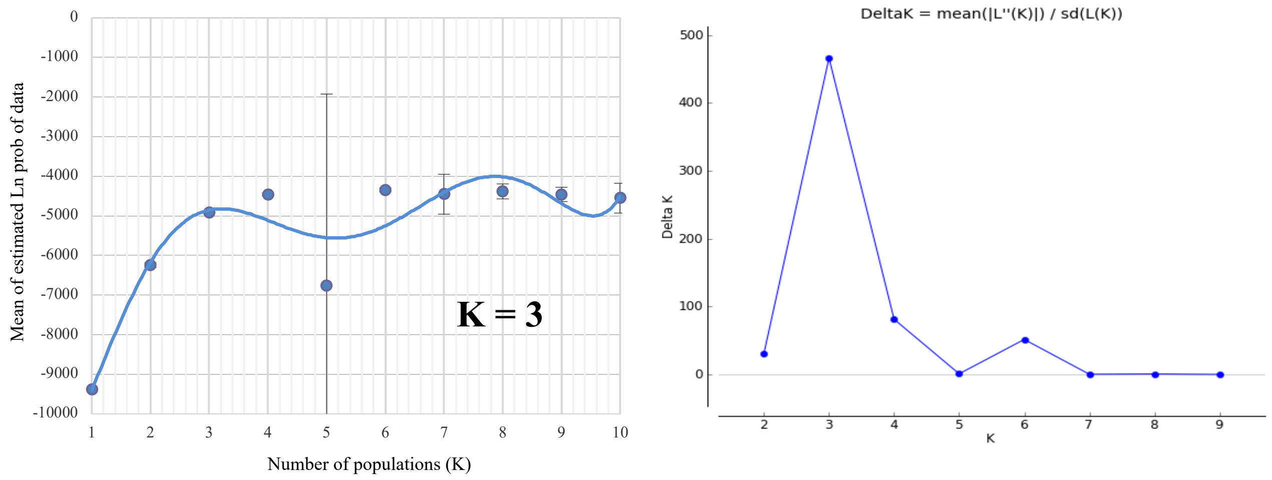

Fig N. Admixture model, LOCPRIOR, independent allele frequency, λ = 0.45

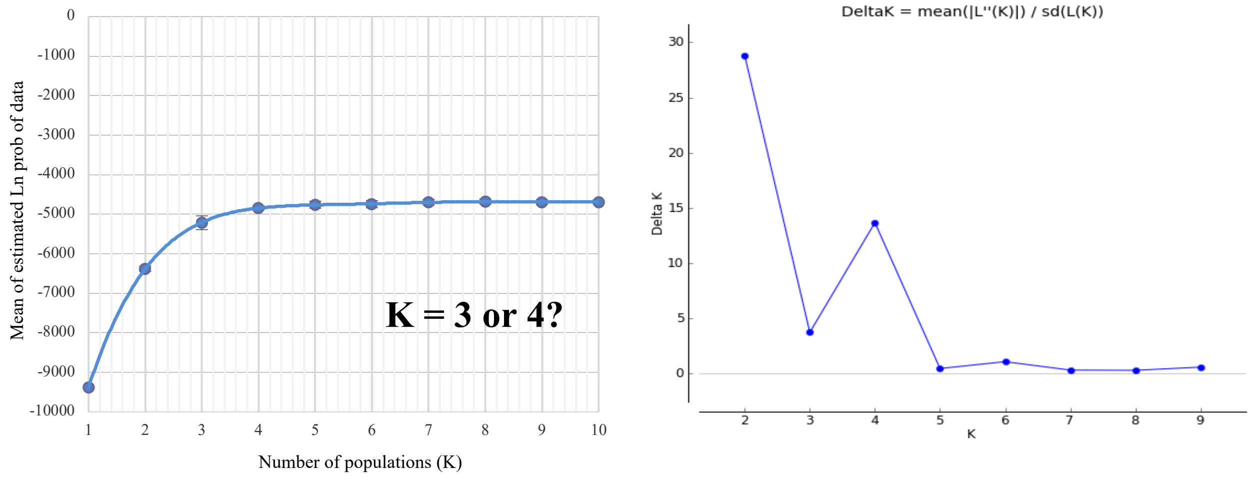

Fig O. Admixture model, POPID, correlated allele frequency, λ = 0.45

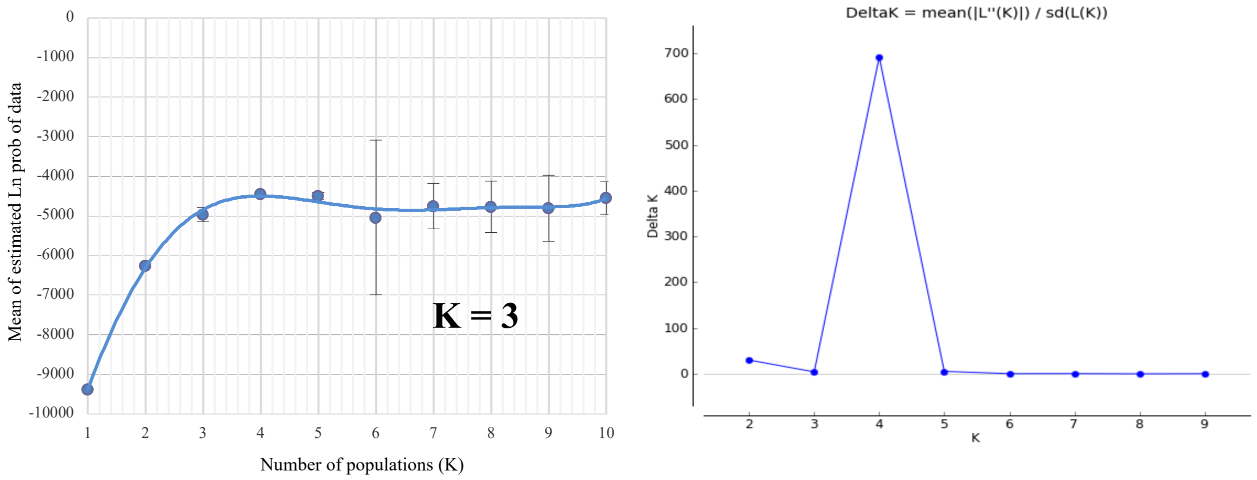

Fig P. Admixture model, POPID, independent allele frequency, λ = 0.45

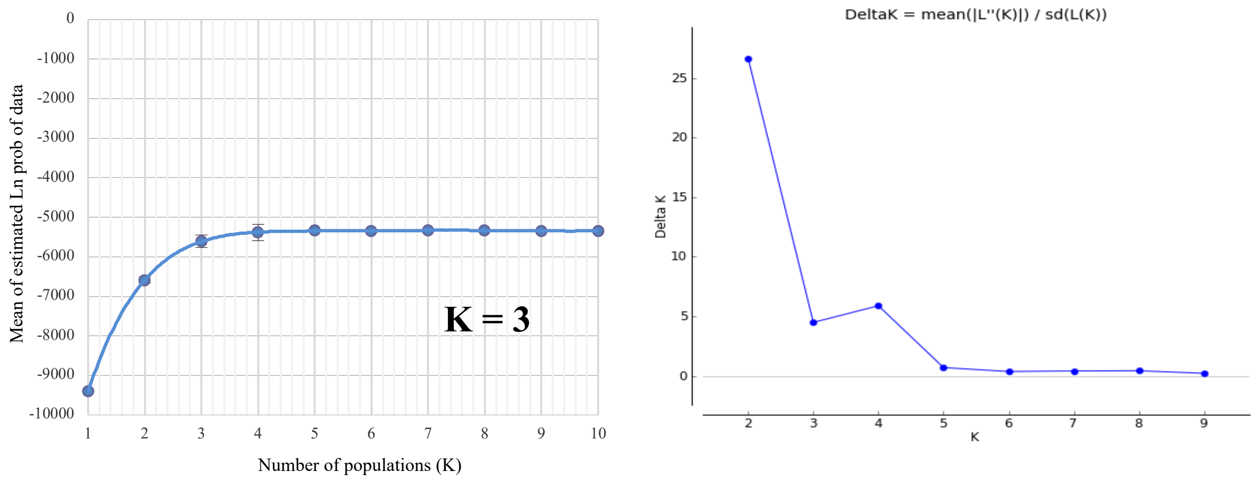

Fig Q. Admixture model, no assignation, correlated allele frequency, λ = 0.45

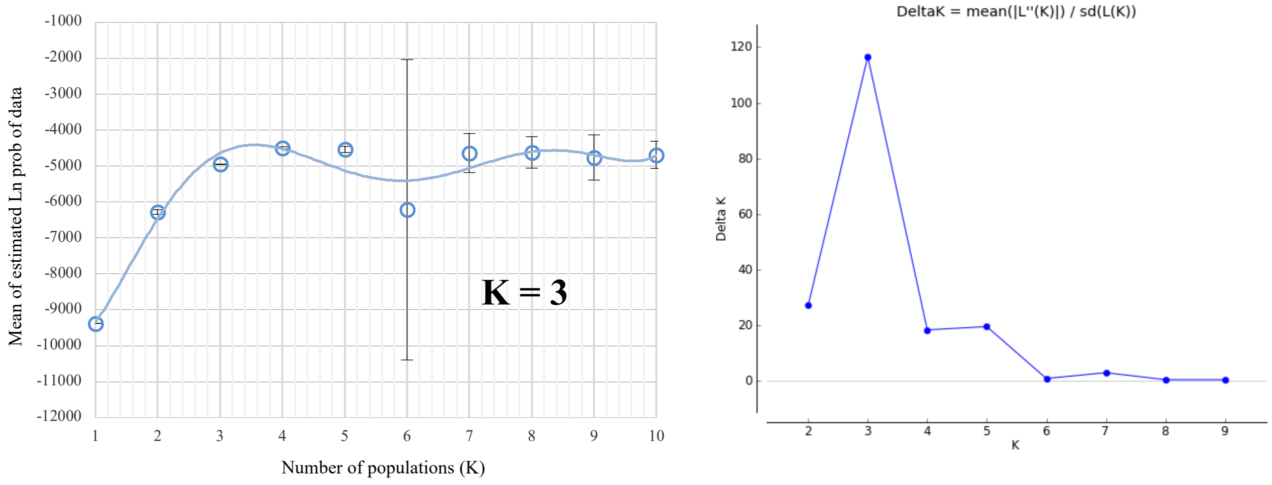

Fig R. Admixture model, no assignation, independent allele frequency, $\lambda = 0.45$

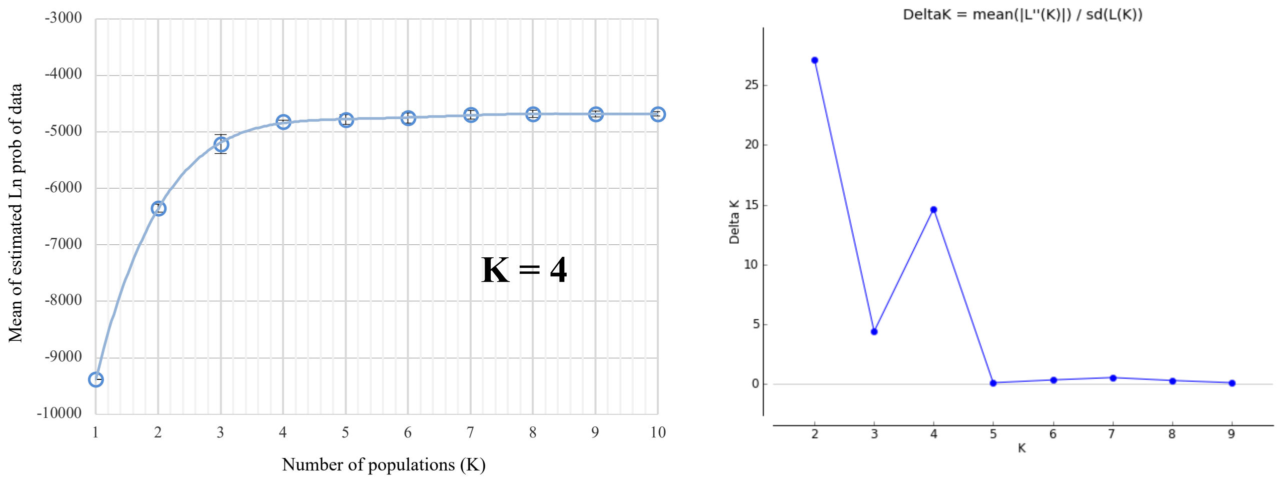

Fig S. No admixture model, LOCPRIOR, correlated allele frequency, $\lambda = 0.45$

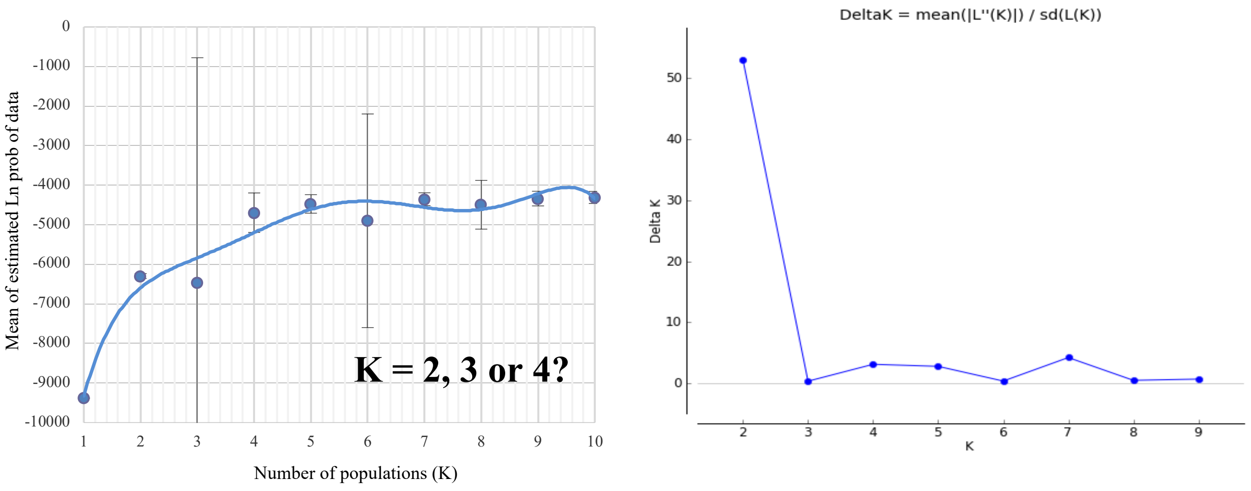

Fig T. No admixture model, LOCPRIOR, independent allele frequency, $\lambda = 0.45$

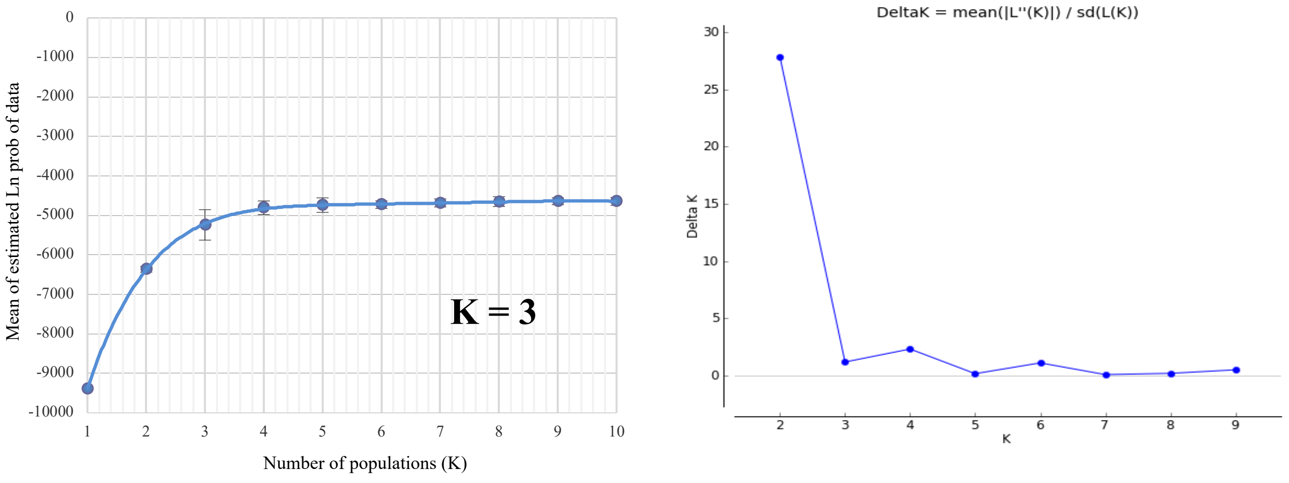

Fig U. No admixture model, POPID, correlated allele frequency, λ = 0.45

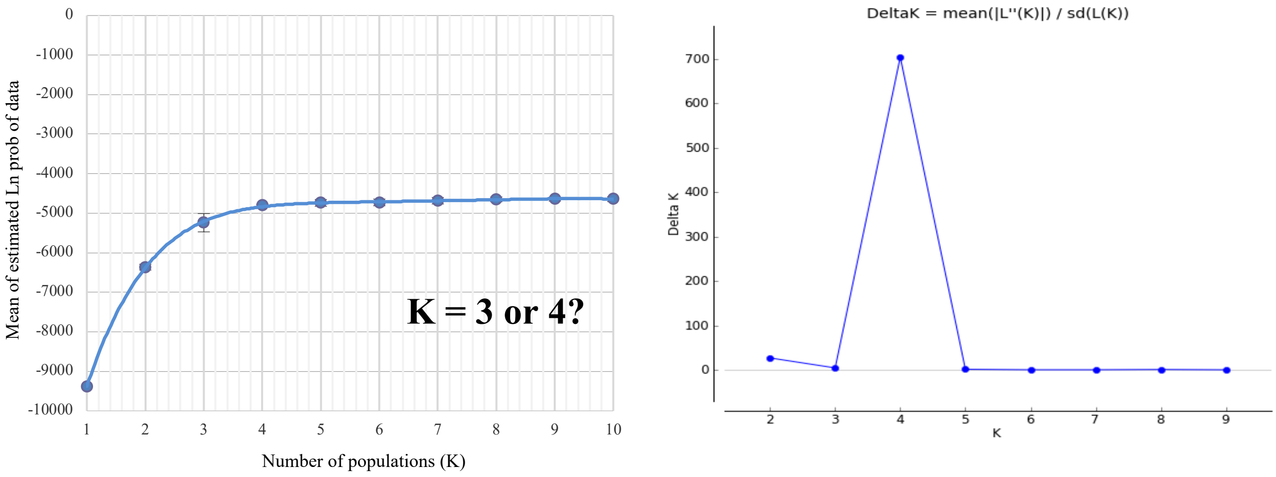

Fig V. No admixture model, POPID, independent allele frequency, λ = 0.45

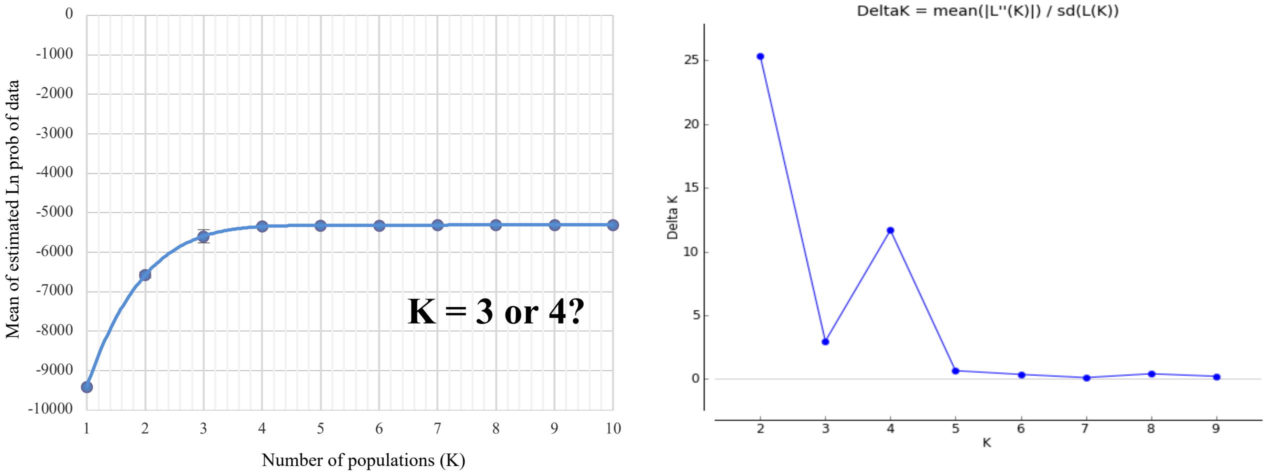

Fig W. No admixture model, no assignation, correlated allele frequency, λ = 0.45

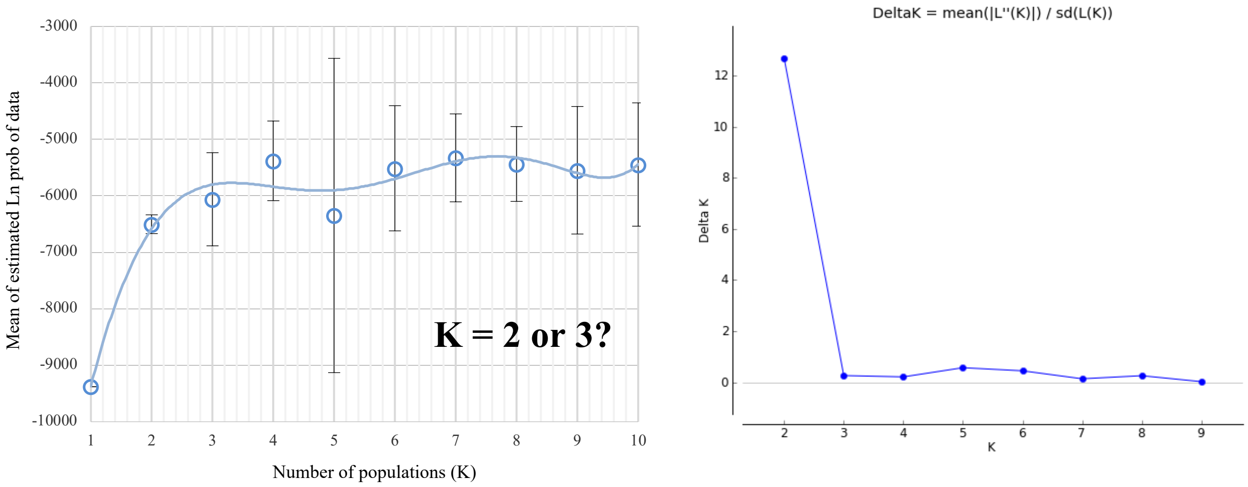

Fig X. No admixture model, no assignation, independent allele frequency, $\lambda = 0.45$

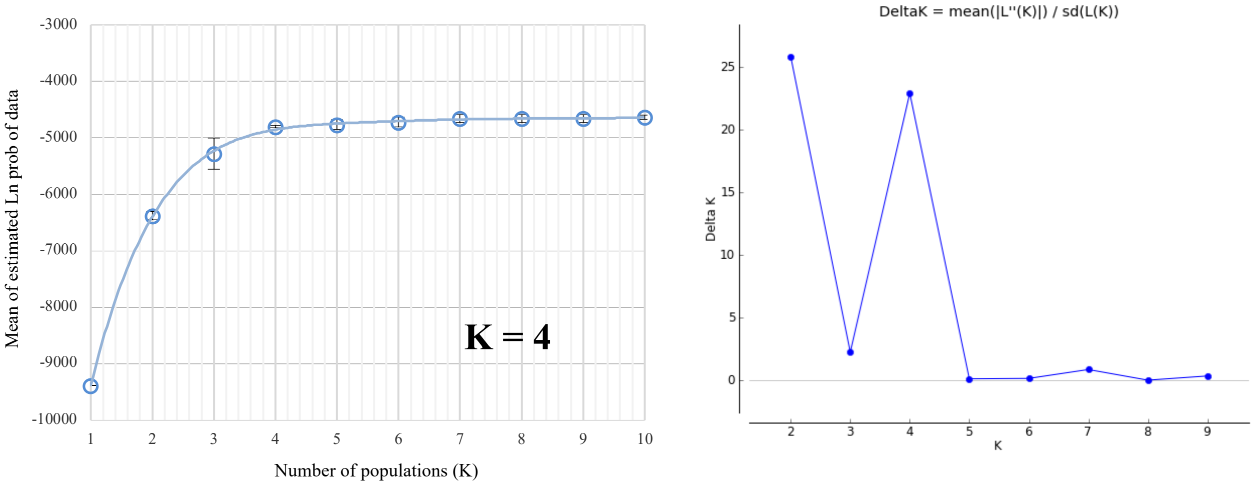

References:

Earl C, von Holdt CM. STRUCTURE HARVESTER: a website and program for visualizing STRUCTURE output and implementing the Evanno method. Conserv Genet Resour. 2011; 4: 359–361.

Pritchard JK, Stephens M, Donnelly P. Inference of population structure using multilocus genotype data. Genetics 2000; 155: 945-959.
