# Supplementary material for: A comparative approach for species delimitation based on multiple methods of multi-locus DNA sequence analysis: A case study of the genus Giraffa (Mammalia, Cetartiodactyla)
Source: PLoS One. 2020 Feb 13;15(2):e0217956. doi: 10.1371/journal.pone.0217956 (PMC7018015; doi:10.1371/journal.pone.0217956)
Supplement: S4 Appendix — (PDF) [file pone.0217956.s004.pdf]

**S4 Appendix.** Genetic distances

# A comparative approach for species delimitation based on multiple methods of multi-locus DNA sequence analysis: a case study of the genus *Giraffa* (Mammalia, Cetartiodactyla)


Alice Petzold[1,2], Alexandre Hassanin[1,2]*

1 Institut de Systématique, Évolution, Biodiversité (ISYEB), Sorbonne Université, MNHN, CNRS, EPHE, Paris, France

2 Muséum national d'Histoire naturelle, CP51, 55 rue Buffon - 75005 Paris, France

*Correspondence: alexandre.hassanin@mnhn.fr


**Table A.** Minimum and maximum pairwise distances (in %), as well as mean distance (between brackets), calculated using the mtDNA-GH82 dataset (1742 nucleotides, *Cytb* and partial control region) both within and between the ten main haplogroups (**I-X**).

| Taxa | I. | II. | III. | IV. | V. | VI. | VIIa. | VIIb. | VIII. | IX. | X. |
|---|---|---|---|---|---|---|---|---|---|---|---|
| **I. Northern haplogroup (N)** [a] | **2.19** | | | | | | | | | | |
| **II.** *G. camelopardalis* sensu stricto B | NA | **1.86** | | | | | | | | | |
| **III.** *G. reticulata* [a] | NA | 1.29-2.19 (1.67) | **1.16** | | | | | | | | |
| **IV.** *G. camelopardalis* sensu stricto C | NA | NA | 0.06-2.06 (1.59) | **1.73** | | | | | | | |
| **V.** *G. peralta* | NA | NA | 1.41-2.19 (1.73) | 1.2-1.86 (1.4) | **0.26** | | | | | | |
| **VI. Eastern haplogroup** [b] | 3.34-4.56 (3.96) | 3.34-4.56 (3.94) | 3.58-4.37 (3.98) | 1.65-4.56 (3.79) | 3.69-4.43 (4.0) | **1.47** | | | | | |
| **VII.a** Masai I | 3.58-4.56 (4.05) | 3.58-4.56 (4.05) | 3.7-4.37 (4.05) | 3.34-4.37 (3.85) | 3.69-4.12 (3.94) | NA | **0.47** | | | | |
| **VII.b** Masai II | 3.34-4.37 (3.89) | 3.34-4.37 (3.87) | 3.58-4.31 (3.93) | 3.58-4.56 (4.03) | 3.81-4.43 (4.11) | NA | 0.77-1.47 (1.09) | **0.1** | | | |
| **VIII. Southeastern haplogroup** | 3.96-4.69 (4.39) | 3.96-4.69 (4.4) | 4.0-4.57 (4.37) | 4.11-4.69 (4.49) | 3.69-4.43 (4.0) | 1.17-2.12 (1.52) | 1.18-2.12 (1.56) | 1.17-1.92 (1.49) | **0.58** | | |
| **IX. Haplogroup E (VI. + VIII.)** | 3.34-4.69 (4.16) | 3.34-4.69 (4.16) | 3.58-4.57 (4.16) | 3.34-4.69 (4.16) | 3.69-4.69 (4.23) | NA | NA | NA | NA | **2.11** | |
| **X. Southwestern haplogroup (S)** | 3.28-4.57 (3.87) | 3.28-4.57 (3.82) | 3.53-4.57 (3.97) | 3.28-4.57 (3.82) | 3.47-4.37 (3.83) | 2.51-3.66 (2.9) | 2.58-3.66 (2.97) | 2.51-3.47 (2.86) | 2.82-3.8 (3.27) | 2.51-3.8 (3.07) | **0.96** |

Roman numerals: respective taxon; NA: not applicable; **boldface**: maximal intrapopulational variation; "a" excluding the individuals RET8 and RET9; "b" excluding the individual TIP15.

**Table B.** Minimum and maximum pairwise distances (in %), as well as mean distance (between brackets) calculated from the nuDNA-G137 dataset (16966 nucleotides) both within and between the nine main taxa (**I-IX**).

| Taxa | I. | II. | III. | IV. | V. | VI. | VII. | VIII. | IX. |
|---|---|---|---|---|---|---|---|---|---|
| **I.** *G. camelopardalis* sensu stricto A | **0.27** | | | | | | | | |
| **II.** *G. camelopardalis* sensu stricto B | NA | **0.13** | | | | | | | |
| **III.** *G. reticulata* | NA | 0.02-0.27 (0.14) | **0.12** | | | | | | |
| **IV.** *G. camelopardalis* sensu stricto C | NA | NA | 0.02-0.2 (0.14) | **0.1** | | | | | |
| **V.** *G. peralta* | NA | NA | 0.04-0.27 (0.14) | 0.01-0.1 (0.07) | **0.1** | | | | |
| **VI.** *G. tippelskirchi* | 0.3-0.54 (0.43) | 0.37-0.54 (0.44) | 0.3-0.5 (0.4) | 0.37-0.54 (0.45) | 0.37-0.51 (0.44) | **0.07** | | | |
| **VII.** *G. camelopardalis* s.s. A + *G. tippelskirchi* | NA | NA | NA | NA | NA | NA | **0.54** | | |
| **VIII.** *G. giraffa* | 0.35-0.62 (0.51) | 0.41-0.62 (0.52) | 0.35-0.55 (0.47) | 0.45-0.62 (0.53) | 0.41-0.58 (0.51) | 0.27-0.44 (0.35) | 0.27-0.62 (0.47) | **0.14** | |
| **IX.** Southern giraffes | 0.33-0.62 (0.5) | 0.37-0.62 (0.5) | 0.33-0.55 (0.46) | 0.37-0.62 (0.51) | 0.37-0.58 (0.5) | NA | NA | NA | **0.44** |

Roman numerals: respective taxon; NA: not applicable; **boldface**: maximal intraspecific variation
