# Supplementary material for: A comparative approach for species delimitation based on multiple methods of multi-locus DNA sequence analysis: A case study of the genus Giraffa (Mammalia, Cetartiodactyla)
Source: PLoS One. 2020 Feb 13;15(2):e0217956. doi: 10.1371/journal.pone.0217956 (PMC7018015; doi:10.1371/journal.pone.0217956)
Supplement: S5 Appendix — (PDF) [file pone.0217956.s005.pdf]

S5 Appendix. Multispecies coalescent approach

A comparative approach for species delimitation based on multiple methods of multi-locus DNA sequence analysis: a case study of the genus *Giraffa* (Mammalia, Cetartiodactyla)

Alice Petzold[1,2], Alexandre Hassanin[1,2]*

1 Institut de Systématique, Évolution, Biodiversité (ISYEB), Sorbonne Université, MNHN, CNRS, EPHE, Paris, France

2 Muséum national d'Histoire naturelle, CP51, 55 rue Buffon - 75005 Paris, France

*Correspondence: alexandre.hassanin@mnhn.fr

Table A. Summary of BPP results obtained in the analyses of the dataset including 21 introns for 66 giraffes.

**Putative species**	**Bayesian Phylogenetics and Phylogeography** (PP)											
	**3S**			**4S**			**5S**			**6S**		
speciesmodelprior	1	2	3	1	2	3	1	2	3	1	2	3
**(C)** *camelopardalis*	-	-	-	-	-	-	-	-	-	-	-	-
**(R1)** *rothschildi*	-	-	-	-	-	-	-	-	-	-	-	-
**(A1)** *antiquorum*	-	-	-	-	-	-	-	-	-	-	-	-
**(P1)** *peralta*	-	-	-	-	-	-	1.0	1.0	1.0	1.0	1.0	1.0
**(A1 R1)** *antiquorum* & *rothschildi*	-	-	-	-	-	-	-	-	-	-	-	-
**(C R1)** *camelopardalis* & *rothschildi*	-	-	-	-	-	-	-	-	-	-	-	-
**(C A1)** *camelopardalis* & *antiquorum*	-	-	-	-	-	-	-	-	-	-	-	-
**(C R1 A1)** *camelopardalis, rothschildi* & *antiquorum*	-	-	-	-	-	-	1.0	1.0	1.0	1.0	1.0	1.0
**(C R1 A1 P1)** *Giraffa camelopardalis* sensu stricto B	-	-	-	1.0	1.0	1.0	-	-	-	-	-	-
**(R2)** *reticulata*	-	-	-	1.0	1.0	1.0	1.0	1.0	1.0	1.0	1.0	1.0
**(C R1 A1 P1 R2)** *Giraffa camelopardalis* sensu stricto A	1.0	1.0	1.0	-	-	-	-	-	-	-	-	-
**(T1)** *tippelskirchi*	-	-	-	-	-	-	-	-	-	0.26	0.4	0.34
**(T2)** *thornicrofti*	-	-	-	-	-	-	-	-	-	0.26	0.4	0.34
**(T1 T2)** *Giraffa tippelskirchi*	1.0	1.0	1.0	1.0	1.0	1.0	1.0	1.0	1.0	0.74	0.6	0.65
**(G)** *giraffa*	-	-	-	-	-	-	-	-	-	-	-	-
**(A2)** *angolensis*	-	-	-	-	-	-	-	-	-	-	-	-
**(G A2)** *Giraffa giraffa*	1.0	1.0	1.0	1.0	1.0	1.0	1.0	1.0	1.0	1.0	1.0	1.0

PP: average posterior probability values; “-“: not found.

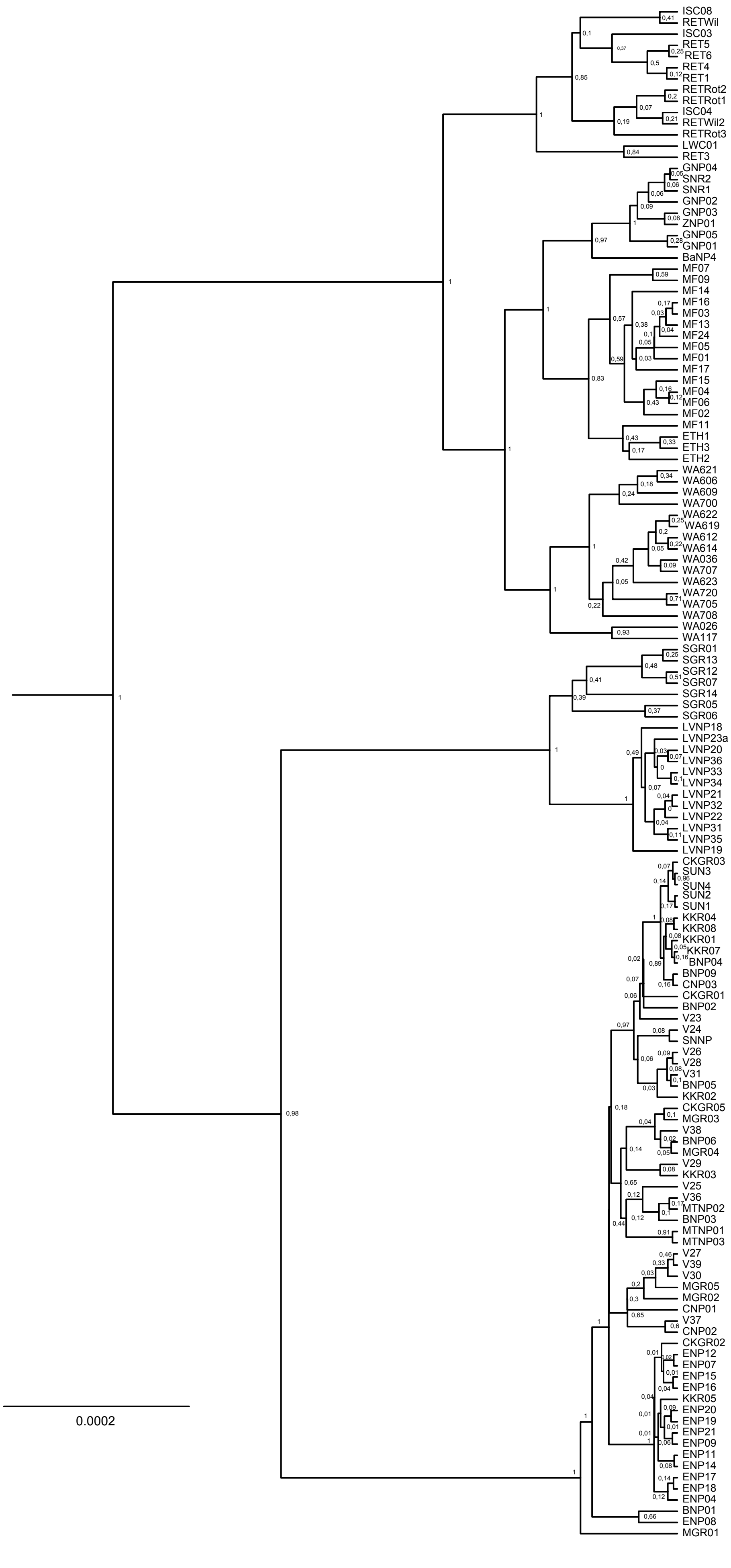

Fig A. MSC species-tree constructed from the nuDNA-G274O6 dataset using *BEAST with the posterior probability values indicated for each node (outgroup taxa *Bos*, *Ovis*, *Okapia* not shown).
