# Supplementary material for: A comparative approach for species delimitation based on multiple methods of multi-locus DNA sequence analysis: A case study of the genus Giraffa (Mammalia, Cetartiodactyla)
Source: PLoS One. 2020 Feb 13;15(2):e0217956. doi: 10.1371/journal.pone.0217956 (PMC7018015; doi:10.1371/journal.pone.0217956)
Supplement: S6 Appendix — (PDF) [file pone.0217956.s006.pdf]

S6 Appendix. Analyses of nuclear haplotypes

A comparative approach for species delimitation based on multiple methods of multi-locus DNA sequence analysis: a case study of the genus *Giraffa* (Mammalia, Cetartiodactyla)

Alice Petzold[1,2], Alexandre Hassanin[1,2]*

1 Institut de Systématique, Évolution, Biodiversité (ISYEB), Sorbonne Université, MNHN, CNRS, EPHE, Paris, France

2 Muséum national d'Histoire naturelle, CP51, 55 rue Buffon - 75005 Paris, France

*Correspondence: alexandre.hassanin@mnhn.fr

List of Locality Abbreviations

**BaNP** =	Badingilo National Park Sudan
**BNP** =	Bwabwata National Park Namibia
**CNP** =	Chobe National Park Botswana
**ETH** =	Gambella National Park Ethiopia
**ENP** =	Etosha National Park Namibia
**GNP** =	Garamba National Park Democratic Republic of Congo
**ISC** =	Ishqbini Conservancy, Kenya
**KKR** =	Khamab Kalahari Reserve South Africa
**LVNP** =	Luangwa Valley National Park Zambia
**LWC** =	Lewa Wildlife Conservancy Kenya
**MGR** =	Moremi Game Reserve Botswana
**MTNP** =	Mosi-oa Tunya National Park Zambia
**MF** =	Murchison Falls National Park Uganda
**RET** =	Nuernberg/ Stuttgart Zoo Germany
**RETRot** =	Rotterdam Zoo Netherlands
**RETWil** =	Wilhelma Zoo Stuttagart Germany
**SGR** =	Selous Game Reserve Tanzania
**SNR** =	Shambe National Park Sudan
**SNNP** =	Sioma Ngwezi National Park Zambia
**SUN** =	Sun Hotel Livingstone Zambia
**WA** =	Koure Niger
**V** =	Vumbura Concession Botswana
**ZNP** =	Zakouma National Park Chad

Tables A-U. Lists of alleles comprised by the haplotypes shown in the median-joining network constructed for each of the 21 nuclear introns (Fig 3).

Table A. Overview of the respective alleles comprised by the haplotypes shown in the network of *ACP5*.

n	Representative individual	∑	Included Alleles per Haplotypes
1	*rothschildi*_ MF03_1	2	MF03_1; MF05_1
2	*rothschildi*_MF11_1	1	MF11_1
3	*thornicrofti*_ LVNP36_1	3	LVNP36_1; MF11_2; RETWil2_2
4	*thornicrofti*_ LVNP22_1	9	LVNP22_1; LVNP23a_1; LVNP23a_2; LVNP31_1; LVNP31_2; LVNP32_1; LVNP32_2; LVNP34_1; LVNP34_2
5	*thornicrofti*_ LVNP33_1	2	LVNP33_1; LVNP33_2
6	*thornicrofti*_ LVNP20_1	19	LVNP20_1; LVNP20_2; LVNP22_2; LVNP35_1; LVNP35_2; LVNP36_2; SGR01_1; SGR01_2; SGR05_1; SGR05_2; SGR06_1; SGR06_2; SGR07_1; SGR12_1; SGR12_2; SGR13_1; SGR13_2; SGR14_1; SGR14_2
7	*thornicrofti*_ LVNP18_1	6	LVNP18_1; LVNP18_2; LVNP19_1; LVNP19_2; LVNP21_1; LVNP21_2
8	*tippelskichi*_SGR07_2	1	SGR07_2
9	*rothschildi*_ MF14_1	2	MF14_1; WA705_1
10	*reticulata*_ RETWil_1	2	RETWil_1; RETWil_2
11	*reticulata*_ RET5_2	1	RET5_2
12	*peralta*_WA708_1	1	WA708_1
13	*antiquorum*_GNP03_2		GNP03_2
14	*camelopardalis*_ ETH1_1	63	ETH1_1; ETH1_2; ETH2_1; ETH2_2; GNP01_1; GNP01_2; GNP02_1; GNP02_2; GNP03_1; GNP04_1; GNP05_1; GNP05_2; MF01_1; MF01_2; MF02_1; MF02_2; MF03_2; MF04_1; MF04_2; MF05_2; MF06_1; MF06_2; MF07_1; MF07_2; MF09_1; MF09_2; MF13_1; MF13_2; MF14_2; MF15_1; MF15_2; MF16_1; MF16_2; MF17_1; MF17_2; MF24_1; MF24_2; RETRot3_1; SNR1_1; SNR2_1; SNR2_2; WA026_1; WA036_1; WA036_2; WA117_1; WA606_1; WA606_2; WA614_1; WA619_1; WA621_1; WA621_2; WA622_1; WA623_1; WA623_2; WA700_1; WA700_2; WA705_2; WA707_1; WA707_2; WA708_2; WA720_1; WA720_2; ZNP01_1
15	*reticulata*_RET1_1	20	RET1_1; RET1_2 ; RET3_1 ; RET3_2 ; RET4_1 ; RET4_2 ; RET5_1 ; RET6_1 ; RET6_2 ; RETRot1_1 ; RETRot1_2 ; RETRot2_1 ; RETRot2_2 ; RETRot3_2 ; ISC03_1 ; ISC03_2; ISC04_1; ISC04_2; LWC01_1; LWC01_2
16	*reticulata*_RETWil2_1	1	RETWil2_1
17	*reticulata*_ISC08_1	1	ISC08_1
18	*reticulata*_ISC08_2	1	ISC08_2
19	*camelopardalis*_BaNP4_1	16	BaNP4_1; BaNP4_2; ETH3_1; ETH3_2; GNP04_2; SNR1_2; WA026_2; WA117_2; WA609_1; WA609_2; WA612_1; WA612_2; WA614_2; WA619_2; WA622_2; ZNP01_2
20	*giraffa*_V24_2	1	V24_2
21	*giraffa*_V25_2	1	V25_2
22	*giraffa*_MGR05_2	5	MGR05_2; MGR05_2; MTNP01_2; MTNP03_2; SNNP_2
23	*giraffa*_CNP03_2	9	CNP03_2; MGR01_1; MGR01_2; MGR02_2; V27_2; V37_1; V37_2; V38_2; V39_2
24	*angolensis*_ENP07_2	4	ENP07_2; ENP11_2; ENP14_2; ENP17_2

25	*giraffa_* CNP01_1	4	CNP01_1; CNP01_2; V23_2; V36_2
26	*giraffa_*BNP09_2	1	BNP09_2
27	*giraffa_*BNP01_1	98	BNP01_1; BNP01_2; BNP02_1; BNP02_2; BNP03_1; BNP03_2; BNP04_1; BNP04_2; BNP05_1; BNP05_2; BNP06_1; BNP06_2; BNP09_1; CKGR01_1; CKGR01_2; CKGR02_1; CKGR02_2; CKGR03_1; CKGR03_2; CKGR05_1; CKGR05_2; CNP02_1; CNP02_2; CNP03_1; ENP04_1; ENP04_2; ENP07_1; ENP08_1; ENP08_2; ENP09_1; ENP09_2; ENP11_1; ENP12_1; ENP12_2; ENP14_1; ENP15_1; ENP15_2; ENP16_1; ENP16_2; ENP17_1; ENP18_1; ENP18_2; ENP19_1; ENP19_2; ENP20_1; ENP20_2; ENP21_1; ENP21_2; KKR01_1; KKR01_2; KKR02_1; KKR02_2; KKR03_1; KKR03_2; KKR04_1; KKR04_2; KKR05_1; KKR05_2; KKR07_1; KKR07_2; KKR08_1; KKR08_2; MGR02_1; MGR03_1; MGR03_2; MGR04_1; MGR04_2; MGR05_1; MTNP01_1; MTNP02_1; MTNP02_2; MTNP03_1; SNNP_1; SUN1_1; SUN1_2; SUN2_1; SUN2_2; SUN3_1; SUN3_2; SUN4_1; SUN4_2; V23_1; V24_1; V25_1; V26_1; V26_2; V27_1; V28_1; V28_2; V29_1; V29_2; V30_1; V30_2; V31_1; V31_2; V36_1; V38_1; V39_1

Table B. Overview of the respective alleles comprised by the haplotypes shown in the network of *C1orf74*.

n	Representative individual	∑	Included Alleles per Haplotypes
1	*reticulata_*RET1_2	5	RET1_2; RET4_2; RET5_2; RET6_1; RET6_2
2	*reticulata_*ISC03_2	1	ISC03_2
3	*rothschildi_*MF01_1	12	MF01_1; MF01_2; MF02_2; MF03_1; MF03_2; MF05_2; MF13_1; MF13_2; MF16_1; MF16_2; MF17_2; RETWil2_2
4	*rothschildi_*MF05_1	8	MF05_1; MF06_1; MF06_2; MF15_1; MF15_2; RET3_2; RETRot3_2; LWC01_2
5	*rothschildi_*MF17_1	1	MF17_1
6	*reticulata_*RETRot1_2	5	RETRot1_2; RETWil_2; RETWil2_1; ISC04_2; ISC08_2
7	*thornicrofti_*LVNP18_1	36	LVNP18_1; LVNP18_2; LVNP19_1; LVNP19_2; LVNP20_1; LVNP20_2; LVNP21_1; LVNP21_2; LVNP22_1; LVNP22_2; LVNP23a_1; LVNP23a_2; LVNP31_1; LVNP31_2; LVNP32_1; LVNP32_2; LVNP33_1; LVNP33_2; LVNP34_1; LVNP34_2; LVNP35_1; LVNP35_2; LVNP36_1; LVNP36_2; SGR01_1; SGR01_2; SGR05_1; SGR05_2; SGR06_1; SGR07_1; SGR07_2; SGR12_1; SGR12_2; SGR13_1; SGR13_2; SGR14_2
8	*camelopardalis_*BaNP4_1	4	BaNP4_1; BaNP4_2; MF07_1; MF07_2
9	*camelopardalis_*ETH1_1	78	ETH1_1; ETH1_2; ETH2_1; ETH2_2; ETH3_1; ETH3_2; GNP01_1; GNP01_2; GNP02_1; GNP02_2; GNP03_1; GNP03_2; GNP04_1; GNP04_2; GNP05_1; GNP05_2; MF02_1; MF04_1; MF04_2; MF09_1; MF09_2; MF11_1; MF11_2; MF14_1; MF14_2; MF24_1; MF24_2; RET1_1; RET3_1; RET4_1; RET5_1; RETRot1_1; RETRot2_1; RETRot2_2; RETRot3_1; RETWil_1; ISC03_1; ISC04_1; ISC08_1; LWC01_1; SNR1_1; SNR1_2; SNR2_1; SNR2_2; WA026_1; WA026_2; WA036_1; WA036_2; WA117_1; WA117_2; WA606_1; WA606_2; WA609_1; WA609_2; WA612_1; WA612_2; WA614_1; WA614_2; WA619_1; WA619_2; WA621_1; WA621_2; WA622_1; WA622_2; WA623_1; WA623_2; WA700_1; WA700_2; WA705_1; WA705_2; WA707_1; WA707_2; WA708_1; WA708_2; WA720_1; WA720_2; ZNP01_1; ZNP01_2
10	*tippelskichi_*SGR06_2	1	SGR06_2

11	*tippelskichi*_SGR14_1	1	SGR14_1
12	*giraffa*_BNP01_1	122	BNP01_1; BNP01_2; BNP02_1; BNP02_2; BNP03_1; BNP03_2; BNP04_1; BNP04_2; BNP05_1; BNP05_2; BNP06_1; BNP06_2; BNP09_1; BNP09_2; CKGR01_1; CKGR01_2; CKGR02_1; CKGR02_2; CKGR03_1; CKGR03_2; CKGR05_1; CKGR05_2; CNP01_1; CNP01_2; CNP02_1; CNP02_2; CNP03_1; CNP03_2; ENP04_1; ENP04_2; ENP07_1; ENP07_2; ENP08_1; ENP08_2; ENP09_1; ENP09_2; ENP11_1; ENP11_2; ENP12_1; ENP12_2; ENP14_1; ENP14_2; ENP15_1; ENP15_2; ENP16_1; ENP16_2; ENP17_1; ENP17_2; ENP18_1; ENP18_2; ENP19_1; ENP19_2; ENP20_1; ENP20_2; ENP21_1; ENP21_2; KKR01_1; KKR01_2; KKR02_1; KKR02_2; KKR03_1; KKR03_2; KKR04_1; KKR04_2; KKR05_1; KKR05_2; KKR07_1; KKR07_2; KKR08_1; KKR08_2; MGR01_1; MGR01_2; MGR02_1; MGR02_2; MGR03_1; MGR03_2; MGR04_1; MGR04_2; MGR05_1; MGR05_2; MTNP01_1; MTNP01_2; MTNP02_1; MTNP02_2; MTNP03_1; MTNP03_2; SNNP_1; SNNP_2; SUN1_1; SUN1_2; SUN2_1; SUN2_2; SUN3_1; SUN3_2; SUN4_1; SUN4_2; V23_1; V23_2; V24_1; V24_2; V25_1; V25_2; V26_1; V26_2; V27_1; V27_2; V28_1; V28_2; V29_1; V29_2; V30_1; V30_2; V31_1; V31_2; V36_1; V36_2; V37_1; V37_2; V38_1; V38_2; V39_1; V39_2

Table C. Overview of the respective alleles comprised by the haplotypes shown in the network of *CCT2*.

n	Representative individual	∑	Included Alleles per Haplotypes
1	*reticulata*_RETRot1_1	5	RETRot1_1; RETRot1_2; RETRot2_1; RETRot2_2; RETWil2_2
2	*reticulata*_RET4_2	4	RET4_2; RETRot3_2; RETWil2_1; ISC04_2
3	*antiquorum*_SNR_2	1	SNR_2
4	*peralta*_WA026_2	1	WA026_2
5	*camelopardalis*_ BaNP4_1	177	BaNP4_1; BNP02_1; BNP02_2; BNP03_1; BNP03_2; BNP04_1; BNP04_2; BNP05_1; BNP05_2; BNP06_1; BNP06_2; BNP09_1; BNP09_2; CKGR01_1; CKGR01_2; CKGR02_1; CKGR02_2; CKGR03_1; CKGR03_2; CKGR05_1; CKGR05_2; CNP01_1; CNP01_2; CNP02_1; CNP02_2; CNP03_1; CNP03_2; ENP04_1; ENP04_2; ENP07_1; ENP07_2; ENP09_1; ENP09_2; ENP11_1; ENP11_2; ENP12_1; ENP12_2; ENP14_1; ENP14_2; ENP15_1; ENP15_2; ENP16_1; ENP16_2; ENP17_1; ENP17_2; ENP18_1; ENP18_2; ENP19_1; ENP19_2; ENP20_1; ENP20_2; ENP21_1; ENP21_2; GNP01_1; GNP05_1; GNP05_2; KKR01_1; KKR01_2; KKR02_1; KKR02_2; KKR03_1; KKR03_2; KKR04_1; KKR04_2; KKR05_1; KKR05_2; KKR07_1; KKR07_2; KKR08_1; KKR08_2; LVNP18_1; LVNP18_2; LVNP19_1; LVNP19_2; LVNP20_1; LVNP20_2; LVNP21_1; LVNP21_2; LVNP22_1; LVNP22_2; LVNP23a_1; LVNP23a_2; LVNP31_1; LVNP31_2; LVNP32_1; LVNP32_2; LVNP33_1; LVNP33_2; LVNP34_1; LVNP34_2; LVNP35_1; LVNP35_2; LVNP36_1; LVNP36_2; MF01_1; MF03_1; MF06_1; MGR01_1; MGR01_2; MGR02_1; MGR02_2; MGR03_1; MGR03_2; MGR04_1; MGR04_2; MGR05_1; MGR05_2; MTNP01_1; MTNP01_2; MTNP02_1; MTNP02_2; MTNP03_1; MTNP03_2; RET1_1; RET5_1; RET6_1; RETWil_1; SGR01_1; SGR01_2; SGR05_1; SGR05_2; SGR06_1; SGR06_2; SGR07_1; SGR07_2; SGR12_1; SGR12_2; SGR13_1; SGR13_2; SGR14_1; SGR14_2; SNNP_1; SNNP_2; SNR1_1; SUN1_1; SUN1_2; SUN2_1; SUN2_2; SUN3_1; SUN3_2; SUN4_1; SUN4_2; V23_1; V23_2; V24_1; V24_2; V25_1; V25_2; V26_1; V26_2; V27_1;

			V27_2; V28_1; V28_2; V29_1; V29_2; V30_1; V30_2; V31_1; V31_2; V36_1; V36_2; V37_1; V37_2; V38_1; V38_2; V39_1; V39_2; WA026_1; WA036_1; WA117_1; WA117_2; WA606_1; WA707_1; WA708_1; WA708_2; ZNP01_1
**6**	***camelopardalis*_ BaNP4_2**	**86**	BaNP4_2; BNP01_1; BNP01_2; ENP08_1; ENP08_2; ETH1_1; ETH1_2; ETH2_1; ETH2_2; ETH3_1; ETH3_2; GNP01_2; GNP02_1; GNP02_2; GNP03_1; GNP03_2; GNP04_1; GNP04_2; MF01_2; MF02_1; MF02_2; MF03_2; MF04_1; MF04_2; MF05_1; MF05_2; MF06_2; MF07_1; MF07_2; MF09_1; MF09_2; MF11_1; MF11_2; MF13_1; MF13_2; MF14_1; MF14_2; MF15_1; MF15_2; MF16_1; MF16_2; MF17_1; MF17_2; MF24_1; MF24_2; RET1_2; RET3_1; RET3_2; RET4_1; RET5_2; RET6_2; RETRot3_1; RETWil_2; ISC03_1; ISC03_2; ISC04_1; ISC08_1; ISC08_2; LWC01_1; LWC01_2; SNR1_2; SNR2_1; WA036_2; WA606_2; WA609_1; WA609_2; WA612_1; WA612_2; WA614_1; WA614_2; WA619_1; WA619_2; WA621_1; WA621_2; WA622_1; WA622_2; WA623_1; WA623_2; WA700_1; WA700_2; WA705_1; WA705_2; WA707_2; WA720_1; WA720_2; ZNP01_2

Table D. Overview of the respective alleles comprised by the haplotypes shown in the network of *COL5A2*.

n	Representative individual	∑	Included Alleles per Haplotypes
**1**	***reticulata*_RETRot3_2**	**2**	RETRot3_2; RETWil_1
**2**	***reticulata*_RETRot3_1**	**1**	RETRot3_1
**3**	***reticulata*_ISC08_2**	**1**	ISC08_2
**4**	***reticulata*_RETWil_2**	**1**	RETWil_2
**5**	***reticulata*_RET1_2**	**3**	RET1_2; RET4_2; ISC03_2
**6**	***reticulata*_RET3_1**	**8**	RET3_1; RET4_1; RET5_1; RET5_2; RET6_1; ISC03_1; ISC04_1; LWC01_1
**7**	***peralta*_WA707_1**	**2**	WA707_1; WA707_2
**8**	***peralta*_WA117_1**	**1**	WA117_1
**9**	***peralta*_WA117_2**	**1**	WA117_2
**10**	***peralta*_WA623_2**	**1**	WA623_2
**11**	***peralta*_WA026_1**	**11**	WA026_1; WA026_2; WA036_1; WA036_2; WA612_2; WA619_2; WA622_1; WA622_2; WA623_1; WA700_2; WA720_2
**12**	***camelopardalis*_ BaNP4_1**	**17**	BaNP4_1; BaNP4_2; MF01_1; MF03_1; MF04_1; MF04_2; MF06_1; MF09_1; MF13_1; MF14_1; MF14_2; MF15_1; MF15_2; MF16_1; MF16_2; MF17_1; MF24_1
**13**	***camelopardalis*_ETH1_1**	**51**	ETH1_1; ETH1_2; ETH2_1; ETH2_2; ETH3_1; ETH3_2; GNP01_1; GNP01_2; GNP02_1; GNP02_2; GNP03_1; GNP03_2; GNP04_1; GNP04_2; GNP05_1; GNP05_2; MF01_2; MF03_2; MF05_1; MF05_2; MF06_2; MF07_1; MF07_2; MF11_1; MF11_2; MF17_2; MF24_2; RETRot1_1; RETRot2_1; RETRot2_2; SNR1_1; SNR1_2; SNR2_1; SNR2_2; WA606_1; WA606_2; WA609_1; WA612_1; WA614_1; WA614_2; WA619_1; WA621_1; WA621_2; WA700_1; WA705_1; WA705_2; WA708_1; WA708_2; WA720_1; ZNP01_1; ZNP01_2
**14**	***rothschildi*_MF02_1**	**2**	MF02_1; MF13_2
**15**	***rothschildi*_MF02_2**	**7**	MF02_2; MF09_2; RET3_2; RETWil2_1; RETWil2_2; LWC01_2; WA609_2

**16**	***thornicrofti*_LVNP18_1**	**33**	LVNP18_1; LVNP18_2; LVNP19_1; LVNP19_2; LVNP20_1; LVNP20_2; LVNP21_1; LVNP21_2; LVNP22_1; LVNP22_2; LVNP23a_1; LVNP23a_2; LVNP31_1; LVNP32_1; LVNP32_2; LVNP33_1; LVNP34_1; LVNP35_1; LVNP35_2; LVNP36_1; LVNP36_2; SGR01_1; SGR01_2; SGR05_1; SGR05_2; SGR06_1; SGR06_2; SGR07_2; SGR12_1; SGR12_2; SGR13_1; SGR14_1; SGR14_2
**17**	***thornicrofti*_LVNP33_2**	**2**	LVNP33_2; LVNP34_2
**18**	***thornicrofti*_LVNP31_2**	**1**	LVNP31_2
**19**	***tippelskirchi*_SGR07_1**	**1**	SGR07_1
**20**	***tippelskirchi*_SGR13_2**	**1**	SGR13_2
**21**	***giraffa*_V26_2**	**1**	V26_2
**22**	***giraffa*_SUN4_2**	**1**	SUN4_2
**23**	***giraffa*_BNP01_1**	**119**	BNP01_1; BNP01_2; BNP02_1; BNP02_2; BNP03_1; BNP03_2; BNP04_1; BNP04_2; BNP05_1; BNP05_2; BNP06_1; BNP09_1; BNP09_2; CKGR01_1; CKGR01_2; CKGR02_1; CKGR02_2; CKGR03_1; CKGR03_2; CKGR05_1; CKGR05_2; CNP01_1; CNP01_2; CNP02_1; CNP02_2; CNP03_1; CNP03_2; ENP04_1; ENP04_2; ENP07_1; ENP07_2; ENP08_1; ENP08_2; ENP09_1; ENP09_2; ENP11_1; ENP11_2; ENP12_1; ENP12_2; ENP14_1; ENP14_2; ENP15_1; ENP15_2; ENP16_1; ENP16_2; ENP17_1; ENP18_1; ENP18_2; ENP19_1; ENP19_2; ENP20_1; ENP20_2; ENP21_1; ENP21_2; KKR01_1; KKR01_2; KKR02_1; KKR02_2; KKR03_1; KKR03_2; KKR04_1; KKR04_2; KKR05_1; KKR05_2; KKR07_1; KKR07_2; KKR08_1; KKR08_2; MGR01_1; MGR02_1; MGR02_2; MGR03_1; MGR03_2; MGR04_1; MGR05_1; MGR05_2; MTNP01_1; MTNP01_2; MTNP02_1; MTNP02_2; MTNP03_1; MTNP03_2; RET1_1; RET6_2; RETRot1_2; ISC04_2; ISC08_1; SNNP_1; SNNP_2; SUN1_1; SUN1_2; SUN2_1; SUN2_2; SUN3_1; SUN3_2; SUN4_1; V23_1; V24_1; V24_2; V25_1; V25_2; V26_1; V26_2; V27_1; V27_2; V28_1; V28_2; V29_1; V30_1; V30_2; V31_1; V31_2; V36_1; V37_1; V37_2; V38_1; V38_2; V39_1; V39_2
**24**	**giraffa_BNP06_2**	**6**	BNP06_2; ENP17_2; MGR01_2; MGR04_2; V23_2; V29_2

Table E. Overview of the respective alleles comprised by the haplotypes shown in the network of *CTAGE5*.

n	Representative individual	∑	Included Alleles per Haplotypes
**1**	***camelopardalis*_ETH1_1**	**66**	ETH1_1; ETH1_2; ETH2_2; ETH3_2; GNP01_1; GNP01_2; GNP02_1; GNP02_2; GNP03_1; GNP03_2; GNP04_1; GNP05_1; GNP05_2; MF01_2; MF03_2; MF04_2; MF05_2; MF06_2; MF07_2; MF09_2; MF11_2; MF13_2; MF14_2; MF15_2; MF16_1; MF16_2; MF17_1; MF17_2; RET3_1; RET3_2; RETRot2_2; RETRot3_2; RETWil_2; ISC03_2; ISC08_2; LWC01_1; LWC01_2; SNR1_1; SNR1_2; SNR2_1; SNR2_2; WA026_1; WA036_2; WA117_1; WA117_2; WA606_1; WA606_2; WA609_1; WA609_2; WA612_1; WA612_2; WA614_1; WA614_2; WA621_1; WA621_2; WA622_1; WA622_2; WA700_1; WA700_2; WA705_2; WA707_1; WA707_2; WA708_2; WA720_1; ZNP01_1; ZNP01_2
**2**	***peralta*_WA026_2**	**2**	WA026_2; WA720_2

**3**	***peralta*_WA619_2**	**4**	WA619_2; WA623_2; WA705_1; WA708_1
**4**	***camelopardalis*_BaNP4_1**	**36**	BaNP4_1; BaNP4_2; ETH3_1; MF01_1; MF02_1; MF02_2; MF03_1; MF04_1; MF05_1; MF06_1; MF07_1; MF09_1; MF11_1; MF13_1; MF14_1; MF15_1; MF24_1; MF24_2; RET1_1; RET1_2; RET4_1; RET4_2; RET5_1; RET5_2; RET6_1; RET6_2; RETRot1_1; RETRot1_2; RETRot2_1; RETRot3_1; RETWil_1; ISC03_1; ISC08_1; WA036_1; WA619_1; WA623_1
**5**	***reticulata*_RETWil2_2**	**2**	RETWil2_2; ISC04_1
**6**	***reticulata*_RETWil2_1**	**1**	RETWil2_1
**7**	***camelopardalis*_ETH2_1**	**1**	ETH2_1
**8**	***reticulata*_ISC04_2**	**1**	ISC04_2
**9**	***antiquorum*_GNP04_2**	**1**	GNP04_2
**10**	***giraffa*_CNP02_1**	**2**	CNP02_1; CNP02_2
**11**	***thornicrofti*_ LVNP18_1**	**30**	LVNP18_1; LVNP18_2; LVNP19_1; LVNP19_2; LVNP20_1; LVNP20_2; LVNP21_1; LVNP21_2; LVNP22_1; LVNP22_2; LVNP23a_1; LVNP23a_2; LVNP31_1; LVNP31_2; LVNP32_1; LVNP32_2; LVNP33_1; LVNP33_2; LVNP34_1; LVNP34_2; LVNP35_1; LVNP35_2; LVNP36_1; LVNP36_2; SGR01_2; SGR06_1; SGR06_2; SGR12_2; SGR14_1; SGR14_2
**12**	***giraffa*_BNP01_1**	**90**	BNP01_1; BNP02_1; BNP02_2; BNP03_1; BNP03_2; BNP04_1; BNP05_1; BNP05_2; BNP06_1; BNP06_2; BNP09_1; CKGR01_1; CKGR01_2; CKGR02_1; CNP01_1; CNP01_2; CNP03_1; CNP03_2; ENP04_1; ENP07_1; ENP07_2; ENP08_1; ENP08_2; ENP09_1; ENP09_2; ENP11_1; ENP11_2; ENP12_1; ENP12_2; ENP14_1; ENP14_2; ENP15_1; ENP16_1; ENP16_2; ENP17_1; ENP18_1; ENP19_1; ENP19_2; ENP20_1; ENP20_2; ENP21_1; ENP21_2; KKR01_1; KKR02_1; KKR02_2; KKR03_1; KKR04_1; KKR04_2; KKR05_1; KKR05_2; MGR01_1; MGR01_2; MGR02_1; MGR02_2; MGR03_1; MGR03_2; MGR04_1; MGR04_2; MGR05_1; MGR05_2; MTNP01_1; MTNP02_1; MTNP02_2; MTNP03_1; SNNP_1; SNNP_2; SUN1_1; SUN2_1; SUN3_1; V23_1; V23_2; V24_1; V25_1; V25_2; V26_1; V26_2; V27_1; V27_2; V28_1; V28_2; V30_1; V30_2; V31_1; V31_2; V36_1; V36_2; V37_1; V37_2; V39_1; V39_2
**13**	***giraffa*_ MTNP01_2**	**3**	MTNP01_2; MTNP03_2; V38_1
**14**	***giraffa*_ BNP04_2**	**5**	BNP04_2; KKR07_1; KKR07_2; V29_2; V38_2
**15**	***giraffa*_ SUN1_2**	**1**	SUN1_2
**16**	***tippelskirchi*_SGR01_1**	**1**	SGR01_1
**17**	***tippelskirchi*_SGR12_1**	**1**	SGR12_1
**18**	***giraffa*_ BNP01_2**	**19**	BNP01_2; BNP09_2; CKGR03_1; CKGR03_2; CKGR05_1; CKGR05_2; ENP04_2; ENP17_2; ENP18_2; KKR01_2; KKR03_2; KKR08_1; KKR08_2; SUN2_2; SUN3_2; SUN4_1; SUN4_2; V24_2; V29_1
**19**	***angolensis*_ENP15_2**	**1**	ENP15_2
**20**	***angolensis*_CKGR02_2**	**1**	CKGR02_2

Table F. Overview of the respective alleles comprised by the haplotypes shown in the network of *CWF19L1*.

n	Representative individual	∑	Included Alleles per Haplotypes
1	***camelopardalis*_BaNP4_1**	79	BaNP4_1; ETH1_1; ETH2_1; ETH2_2; ETH3_1; ETH3_2; GNP01_1; GNP03_1; GNP04_1; GNP04_2; GNP05_1; MF02_1; MF03_1; MF04_1; MF06_1; MF09_2; MF15_2; MF17_2; MF24_2; RET1_1; RET1_2; RET3_1; RET3_2; RET4_1; RET4_2; RET5_1; RET5_2; RET6_1; RET6_2; RETRot1_1; RETRot1_2; RETRot2_1; RETRot2_2; RETRot3_1; RETRot3_2; RETWil_1; RETWil2_1; RETWil2_2; ISC03_1; ISC04_1; ISC04_2; ISC08_1; ISC08_2; LWC01_1; LWC01_2; SNR1_1; SNR1_2; SNR2_1; WA026_1; WA026_2; WA036_1; WA036_2; WA117_1; WA117_2; WA606_1; WA606_2; WA609_1; WA609_2; WA612_1; WA612_2; WA614_1; WA614_2; WA619_1; WA619_2; WA621_1; WA621_2; WA622_1; WA622_2; WA623_1; WA623_2; WA700_1; WA700_2; WA705_1; WA705_2; WA707_1; WA707_2; WA720_1; WA720_2; ZNP01_1
2	***camelopardalis*_BaNP4_2**	28	BaNP4_2; ETH1_2; GNP01_2; GNP02_1; GNP02_2; GNP03_2; GNP05_2; MF01_1; MF01_2; MF02_2; MF03_2; MF04_2; MF05_1; MF05_2; MF06_2; MF07_1; MF07_2; MF09_1; MF11_1; MF11_2; MF15_1; MF16_1; MF16_2; MF17_1; MF24_1; RETWil_2; ISC03_2; SNR2_2
3	***peralta*_WA708_1**	2	WA708_1; WA708_2
4	***rothschildi*_MF13_1**	3	MF13_1; MF14_1; MF14_2
5	***rothschildi*_MF13_2**	1	MF13_2
6	***antiquorum*_ZNP01_2**	1	ZNP01_2
7	***giraffa*_BNP02_2**	27	BNP02_2; BNP03_1; BNP03_2; BNP06_1; BNP06_2; CKGR01_1; CKGR01_2; CNP02_2; ENP04_2; ENP08_2; ENP15_2; ENP16_2; ENP18_1; ENP18_2; ENP19_1; ENP19_2; ENP20_2; ENP21_2; KKR02_1; KKR02_2; KKR04_2; MGR02_2; SGR07_2; SGR12_2; V28_2; V36_2; V38_2
8	***giraffa*_BNP02_1**	130	BNP01_1; BNP01_2; BNP02_1; BNP04_1; BNP04_2; BNP05_1; BNP05_2; BNP09_1; BNP09_2; CKGR02_1; CKGR02_2; CKGR03_1; CKGR03_2; CKGR05_1; CKGR05_2; CNP01_1; CNP01_2; CNP02_1; CNP03_1; CNP03_2; ENP04_1; ENP07_1; ENP07_2; ENP08_1; ENP09_1; ENP09_2; ENP11_1; ENP11_2; ENP12_1; ENP12_2; ENP14_1; ENP14_2; ENP15_1; ENP16_1; ENP17_1; ENP17_2; ENP20_1; ENP21_1; KKR01_1; KKR01_2; KKR03_1; KKR03_2; KKR04_1; KKR05_1; KKR05_2; KKR07_1; KKR07_2; KKR08_1; KKR08_2; LVNP18_1; LVNP18_2; LVNP19_1; LVNP19_2; LVNP20_1; LVNP20_2; LVNP21_1; LVNP21_2; LVNP22_1; LVNP22_2; LVNP23a_1; LVNP23a_2; LVNP31_1; LVNP31_2; LVNP32_1; LVNP32_2; LVNP33_1; LVNP33_2; LVNP34_1; LVNP34_2; LVNP35_1; LVNP35_2; LVNP36_1; LVNP36_2; MGR01_1; MGR01_2; MGR02_1; MGR03_1; MGR03_2; MGR04_1; MGR04_2; MGR05_1; MGR05_2; MTNP01_1; MTNP01_2; MTNP02_1; MTNP02_2; MTNP03_1; MTNP03_2; SGR01_1; SGR01_2; SGR05_1; SGR05_2; SGR06_1; SGR06_2; SGR07_1; SGR12_1; SGR13_1; SGR13_2; SGR14_1; SGR14_2; SNNP_1; SNNP_2; SUN1_1; SUN1_2; SUN2_1; SUN3_1; SUN3_2; SUN4_1; SUN4_2; V23_1; V23_2; V24_1; V24_2; V25_1; V25_2; V26_1; V26_2; V27_1; V28_1; V29_1; V29_2; V30_1; V30_2; V31_1; V31_2; V36_1; V37_1; V37_2; V38_1; V39_1
9	***giraffa*_V27_2**	2	V27_2; V39_2
10	***giraffa*_SUN2_2**	1	SUN2_2

Table G. Overview of the respective alleles comprised by the haplotypes shown in the network of *DDX1*.

n	Representative individual	∑	Included Alleles per Haplotypes
**1**	***camelopardalis*_BaNP4_1**	**1**	BaNP4_1
**2**	***camelopardalis*_BaNP4_2**	**100**	BaNP4_2; ETH1_1; ETH1_2; ETH2_1; ETH2_2; ETH3_1; ETH3_2; GNP01_1; GNP01_2; GNP02_1; GNP02_2; GNP03_1; GNP03_2; GNP04_1; GNP04_2; GNP05_1; GNP05_2; MF01_1; MF01_2; MF02_1; MF02_2; MF03_1; MF03_2; MF04_1; MF04_2; MF05_1; MF05_2; MF06_1; MF06_2; MF07_1; MF07_2; MF09_1; MF09_2; MF11_1; MF11_2; MF13_1; MF13_2; MF14_1; MF14_2; MF15_1; MF15_2; MF16_1; MF16_2; MF17_1; MF17_2; MF24_1; MF24_2; RET1_2; RET3_1; RET3_2; RET6_2; RETRot2_1; RETRot2_2; RETWil_1; RETWil_2; RETWil2_1; ISC03_1; ISC03_2; ISC04_1; ISC08_1; ISC08_2; LWC01_1; SNR1_1; SNR1_2; SNR2_1; SNR2_2; WA026_1; WA026_2; WA036_1; WA036_2; WA117_1; WA117_2; WA606_1; WA606_2; WA609_1; WA609_2; WA612_1; WA612_2; WA614_1; WA614_2; WA619_1; WA619_2; WA621_1; WA621_2; WA622_1; WA622_2; WA623_1; WA623_2; WA700_1; WA700_2; WA705_1; WA705_2; WA707_1; WA707_2; WA708_1; WA708_2; WA720_1; WA720_2; ZNP01_1; ZNP01_2
**3**	***reticulata*_RET1_1**	**10**	RET1_1; RET4_1; RET4_2; RET5_1; RET5_2; RET6_1; RETRot1_1; RETRot1_2; RETRot3_1; RETRot3_2
**4**	***reticulata*_LWC01_2**	**1**	LWC01_2
**5**	***giraffa*_MGR02_2**	**3**	MGR02_2; V27_2; V39_2
**6**	***thornicrofti*_LVNP32_2**	**6**	LVNP32_2; LVNP33_1; LVNP33_2; LVNP36_2; SGR01_2; SGR14_2
**7**	***thornicrofti*_LVNP36_1**	**3**	LVNP36_1; SGR01_1; SGR14_1
**8**	***giraffa*_BNP01_1**	**150**	BNP01_1; BNP01_2; BNP02_1; BNP02_2; BNP03_1; BNP03_2; BNP04_1; BNP04_2; BNP05_1; BNP05_2; BNP06_1; BNP06_2; BNP09_1; BNP09_2; CKGR01_1; CKGR01_2; CKGR02_1; CKGR02_2; CKGR03_1; CKGR03_2; CKGR05_1; CKGR05_2; CNP01_1; CNP01_2; CNP02_1; CNP02_2; CNP03_1; CNP03_2; ENP04_1; ENP04_2; ENP07_1; ENP07_2; ENP08_1; ENP08_2; ENP09_1; ENP09_2; ENP11_1; ENP11_2; ENP12_1; ENP12_2; ENP14_1; ENP14_2; ENP15_1; ENP15_2; ENP16_1; ENP16_2; ENP17_1; ENP17_2; ENP18_1; ENP18_2; ENP19_1; ENP19_2; ENP20_1; ENP20_2; ENP21_1; ENP21_2; KKR01_1; KKR01_2; KKR02_1; KKR02_2; KKR03_1; KKR03_2; KKR04_1; KKR04_2; KKR05_1; KKR05_2; KKR07_1; KKR07_2; KKR08_1; KKR08_2; LVNP18_1; LVNP18_2; LVNP19_1; LVNP19_2; LVNP20_1; LVNP20_2; LVNP21_1; LVNP21_2; LVNP22_1; LVNP22_2; LVNP23a_1; LVNP23a_2; LVNP31_1; LVNP31_2; LVNP32_1; LVNP34_1; LVNP34_2; LVNP35_1; LVNP35_2; MGR01_1; MGR01_2; MGR02_1; MGR03_1; MGR03_2; MGR04_1; MGR04_2; MGR05_1; MGR05_2; MTNP01_1; MTNP01_2; MTNP02_1; MTNP02_2; MTNP03_1; MTNP03_2; RETWil2_2; ISC04_2; SGR05_1; SGR05_2; SGR06_1; SGR06_2; SGR07_1; SGR07_2; SGR12_1; SGR12_2; SGR13_1; SGR13_2; SNNP_1; SNNP_2; SUN1_1; SUN1_2; SUN2_1; SUN2_2; SUN3_1; SUN3_2; SUN4_1; SUN4_2; V23_1; V23_2; V24_1; V24_2; V25_1; V25_2; V26_1; V26_2; V27_1; V28_1; V28_2; V29_1; V29_2; V30_1; V30_2; V31_1; V31_2; V36_1; V36_2; V37_1; V37_2; V38_1; V38_2; V39_1

Table H. Overview of the respective alleles comprised by the haplotypes shown in the network of *DHX36*.

n	Representative individual	∑	Included Alleles per Haplotypes
**1**	***camelopardalis*_ETH1_1**	**39**	ETH1_1; ETH1_2; ETH2_2; ETH3_2; GNP02_2; GNP04_2; MF01_1; MF01_2; MF02_1; MF02_2; MF03_2; MF04_1; MF04_2; MF05_1; MF05_2; MF06_1; MF06_2; MF07_1; MF07_2; MF09_1; MF09_2; MF11_1; MF11_2; MF13_2; MF14_1; MF14_2; MF15_1; MF15_2; MF16_1; MF16_2; MF17_1; MF17_2; MF24_2; LWC01_1; SNR2_1; SNR2_2; WA117_1; WA117_2; WA720_2
**2**	***camelopardalis*_ETH2_1**	**22**	ETH2_1; GNP01_1; GNP01_2; GNP02_1; GNP04_1; GNP05_1; GNP05_2; MF03_1; MF13_1; MF24_1; RET3_1; RET3_2; RET4_1; RETRot1_1; RETWil_2; WA612_2; WA619_1; WA619_2; WA622_2; WA720_1; ZNP01_1; ZNP01_2
**3**	***camelopardalis*_BaNP4_1**	**70**	BaNP4_1; BaNP4_2; ETH3_1; GNP03_1; LVNP18_1; LVNP18_2; LVNP19_1; LVNP19_2; LVNP20_1; LVNP20_2; LVNP21_1; LVNP21_2; LVNP22_1; LVNP22_2; LVNP23a_1; LVNP23a_2; LVNP31_1; LVNP31_2; LVNP32_1; LVNP32_2; LVNP33_1; LVNP33_2; LVNP34_1; LVNP34_2; LVNP35_1; LVNP35_2; LVNP36_1; LVNP36_2; RET1_1; RET1_2; RET5_1; RET6_1; RETRot3_1; RETRot3_2; RETWil_1; RETWil2_1; RETWil2_2; ISC04_1; SGR01_1; SGR01_2; SGR05_1; SGR05_2; SGR06_1; SGR07_1; SGR07_2; SGR12_1; SGR12_2; SGR13_1; SGR13_2; SGR14_1; SGR14_2; SNR1_1; WA026_1; WA026_2; WA036_1; WA606_1; WA606_2; WA609_1; WA612_1; WA614_1; WA622_1; WA623_1; WA623_2; WA700_1; WA700_2; WA705_1; WA705_2; WA707_1; WA708_1; WA708_2
**4**	***reticulata*_RET4_2**	**7**	RET4_2; RET5_2; RET6_2; RETRot1_2; RETRot2_1; ISC03_1; ISC03_2
**5**	***reticulata*_ISC08_1**	**1**	ISC08_1
**6**	***reticulata*_ISC08_2**	**1**	ISC08_2
**7**	***reticulata*_ISC04_2**	**1**	ISC04_2
**8**	***antiquorum*_GNP03_2**	**2**	GNP03_2; SNR1_2
**9**	***reticulata*_RETRot2_2**	**2**	RETRot2_2; LWC01_2
**10**	***peralta*_WA036_2**	**5**	WA036_2; WA614_2; WA621_1; WA621_2; WA707_2
**11**	***peralta*_WA609_2**	**1**	WA609_2
**12**	***tippelskirchi*_SGR06_2**	**1**	SGR06_2
**13**	***giraffa*_BNP01_1**	**122**	BNP01_1; BNP01_2; BNP02_1; BNP02_2; BNP03_1; BNP03_2; BNP04_1; BNP04_2; BNP05_1; BNP05_2; BNP06_1; BNP06_2; BNP09_1; BNP09_2; CKGR01_1; CKGR01_2; CKGR02_1; CKGR02_2; CKGR03_1; CKGR03_2; CKGR05_1; CKGR05_2; CNP01_1; CNP01_2; CNP02_1; CNP02_2; CNP03_1; CNP03_2; ENP04_1; ENP04_2; ENP07_1; ENP07_2; ENP08_1; ENP08_2; ENP09_1; ENP09_2; ENP11_1; ENP11_2; ENP12_1; ENP12_2; ENP14_1; ENP14_2; ENP15_1; ENP15_2; ENP16_1; ENP16_2; ENP17_1; ENP17_2; ENP18_1; ENP18_2; ENP19_1; ENP19_2; ENP20_1; ENP20_2; ENP21_1; ENP21_2; KKR01_1; KKR01_2; KKR02_1; KKR02_2; KKR03_1; KKR03_2; KKR04_1; KKR04_2; KKR05_1; KKR05_2; KKR07_1; KKR07_2; KKR08_1; KKR08_2; MGR01_1; MGR01_2; MGR02_1; MGR02_2; MGR03_1; MGR03_2; MGR04_1; MGR04_2; MGR05_1; MGR05_2; MTNP01_1; MTNP01_2; MTNP02_1; MTNP02_2; MTNP03_1; MTNP03_2; SNNP_1; SNNP_2; SUN1_1; SUN1_2; SUN2_1; SUN2_2; SUN3_1; SUN3_2; SUN4_1; SUN4_2; V23_1; V23_2; V24_1; V24_2; V25_1; V25_2; V26_1; V26_2; V27_1; V27_2; V28_1; V28_2; V29_1; V29_2; V30_1; V30_2; V31_1; V31_2; V36_1; V36_2; V37_1; V37_2; V38_1; V38_2; V39_1; V39_2

Table I. Overview of the respective alleles comprised by the haplotypes shown in the network of *IGF2B1*.

n	Representative individual	∑	Included Alleles per Haplotypes
1	***camelopardalis*_BaNP4_1**	102	BaNP4_1; BaNP4_2; ETH1_1; ETH1_2; ETH2_1; ETH2_2; ETH3_1; ETH3_2; GNP01_1; GNP02_1; GNP02_2; GNP04_1; MF01_1; MF01_2; MF02_1; MF03_1; MF03_2; MF04_1; MF04_2; MF05_1; MF05_2; MF06_1; MF06_2; MF07_1; MF07_2; MF09_1; MF09_2; MF11_1; MF11_2; MF13_1; MF13_2; MF16_1; MF16_2; MF17_1; MF17_2; MF24_1; MF24_2; RET1_1; RET1_2; RET3_1; RET3_2; RET4_1; RET4_2; RET5_1; RET5_2; RET6_1; RET6_2; RETRot1_1; RETRot1_2; RETRot2_1; RETRot3_1; RETRot3_2; RETWil_1; RETWil_2; RETWil2_1; RETWil2_2; ISC03_1; ISC03_2; ISC04_1; ISC08_1; ISC08_2; LWC01_1; LWC01_2; SGR01_1; SGR01_2; SGR05_1; SGR05_2; SGR06_1; SGR06_2; SGR07_1; SGR07_2; SGR12_1; SGR12_2; SGR13_1; SGR13_2; SGR14_1; SGR14_2; SNR1_1; SNR1_2; SNR2_1; WA036_1; WA036_2; WA117_1; WA117_2; WA606_1; WA606_2; WA609_1; WA612_1; WA612_2; WA614_1; WA619_1; WA621_1; WA621_2; WA622_1; WA623_1; WA700_1; WA700_2; WA705_1; WA705_2; WA708_1; WA708_2; WA720_1
2	***antiquorum*_GNP03_1**	5	GNP03_1; GNP03_2; GNP04_2; ZNP01_1; ZNP01_2
3	***rothschildi*_MF14_1**	2	MF14_1; MF14_2
4	***peralta*_WA609_2**	6	WA609_2; WA614_2; WA619_2; WA622_2; WA623_2; WA707_1
5	***antiquorum*_GNP01_2**	3	GNP01_2; GNP05_2; SNR2_2
6	***rothschildi*_MF15_1**	2	MF15_1; MF15_2
7	***rothschildi*_MF02_2**	1	MF02_2
8	***antiquorum*_GNP05_1**	1	GNP05_1
9	***reticulata*_RETRot2_2**	1	RETRot2_2
10	***reticulata*_ISC04_2**	1	ISC04_2
11	***peralta*_WA026_1**	1	WA026_1
12	***peralta*_WA026_2**	1	WA026_2
13	***peralta*_WA720_2**	1	WA720_2
14	***peralta*_WA707_2**	1	WA707_2
15	***tippelskirchi*_LVNP32_1**	1	LVNP32_1
16	***tippelskirchi*_LVNP18_1**	1	LVNP18_1
17	***tippelskirchi*_LVNP18_2**	22	LVNP18_2; LVNP19_1; LVNP19_2; LVNP20_1; LVNP20_2; LVNP21_1; LVNP21_2; LVNP22_1; LVNP22_2; LVNP23a_1; LVNP23a_2; LVNP31_1; LVNP31_2; LVNP32_2; LVNP33_1; LVNP33_2; LVNP34_1; LVNP34_2; LVNP35_1; LVNP35_2; LVNP36_1; LVNP36_2
18	***giraffa*_V38_1**	1	V38_1
19	***giraffa*_V38_2**	1	V38_2
20	***angolensis*_ENP09_2**	4	ENP09_2; ENP17_2; ENP20_2; ENP21_2
21	***giraffa*_MGR02_1**	4	MGR02_1; V25_1; V25_2; V29_1

**22**	***giraffa*_BNP01_1**	**112**	BNP01_1; BNP01_2; BNP02_1; BNP02_2; BNP03_1; BNP03_2; BNP04_1; BNP04_2; BNP05_1; BNP05_2; BNP06_1; BNP06_2; BNP09_1; BNP09_2; CKGR01_1; CKGR01_2; CKGR02_1; CKGR02_2; CKGR03_1; CKGR03_2; CKGR05_1; CKGR05_2; CNP01_1; CNP01_2; CNP02_1; CNP02_2; CNP03_1; CNP03_2; ENP04_1; ENP04_2; ENP07_1; ENP07_2; ENP08_1; ENP08_2; ENP09_1; ENP11_1; ENP11_2; ENP12_1; ENP12_2; ENP14_1; ENP14_2; ENP15_1; ENP15_2; ENP16_1; ENP16_2; ENP17_1; ENP18_1; ENP18_2; ENP19_1; ENP19_2; ENP20_1; ENP21_1; KKR01_1; KKR01_2; KKR02_1; KKR02_2; KKR03_1; KKR03_2; KKR04_1; KKR04_2; KKR05_1; KKR05_2; KKR07_1; KKR07_2; KKR08_1; KKR08_2; MGR01_1; MGR01_2; MGR02_2; MGR03_1; MGR03_2; MGR04_1; MGR04_2; MGR05_1; MGR05_2; MTNP01_1; MTNP01_2; MTNP02_1; MTNP02_2; MTNP03_1; MTNP03_2; SNNP_1; SNNP_2; SUN1_1; SUN1_2; SUN2_1; SUN2_2; SUN3_1; SUN3_2; SUN4_1; SUN4_2; V23_1; V23_2; V24_1; V24_2; V26_1; V26_2; V27_1; V27_2; V28_1; V28_2; V29_2; V30_1; V30_2; V31_1; V31_2; V36_1; V36_2; V37_1; V37_2; V39_1; V39_2

Table J. Overview of the respective alleles comprised by the haplotypes shown in the network of *MACF1*.

n	Representative individual	∑	Included Alleles per Haplotypes
**1**	***camelopardalis*_BaNP4_1**	**108**	BaNP4_1; BaNP4_2; BNP03_1; BNP06_1; CNP01_1; ETH1_1; ETH2_1; ETH3_1; GNP05_1; LVNP18_1; LVNP18_2; LVNP19_1; LVNP19_2; LVNP20_1; LVNP20_2; LVNP21_1; LVNP21_2; LVNP22_1; LVNP22_2; LVNP23a_1; LVNP23a_2; LVNP31_1; LVNP31_2; LVNP32_1; LVNP32_2; LVNP33_1; LVNP33_2; LVNP34_1; LVNP34_2; LVNP35_1; LVNP35_2; LVNP36_1; LVNP36_2; MF01_1; MF02_1; MF03_1; MF05_1; MF06_1; MF07_1; MF09_1; MF11_1; MF13_1; MF13_2; MF14_1; MF16_1; MF17_1; MF24_1; MF24_2; MGR01_1; MGR01_2; MGR02_1; MGR02_2; MGR03_1; MGR03_2; MGR04_1; MGR04_2; MGR05_1; MGR05_2; RET1_1; RET1_2; RET4_1; RET4_2; RET5_1; RET6_1; RET6_2; RETRot1_1; RETRot2_1; RETRot2_2; RETRot3_1; RETRot3_2; RETWil_1; RETWil2_1; RETWil2_2; ISC04_1; ISC04_2; ISC08_1; LWC01_1; LWC01_2; SGR05_1; SGR05_2; SGR06_1; SGR06_2; SGR07_1; SGR07_2; SGR12_1; SGR12_2; SGR14_1; SGR14_2; SNR1_1; SNR1_2; SNR2_1; SNR2_2; V36_1; WA026_1; WA026_2; WA117_1; WA117_2; WA606_1; WA614_1; WA623_1; WA700_1; WA705_1; WA705_2; WA707_1; WA707_2; WA720_1; WA720_2; ZNP01_1
**2**	***giraffa*_CNP02_1**	**2**	CNP02_1; V37_1
**3**	***camelopardalis*_ETH1_2**	**6**	ETH1_2; GNP01_2; WA606_2; WA612_1; WA614_2; WA621_1;
**4**	***peralta*_WA036_1**	**8**	WA036_1; WA036_2; WA609_1; WA609_2; WA621_2; WA623_2; WA700_2; WA708_2
**5**	***angolensis*_ENP04_1**	**22**	ENP04_1; ENP08_1; ENP17_1; ENP19_1; ENP20_1; KKR03_1; KKR05_1; MTNP01_1; MTNP01_2; MTNP02_1; MTNP03_1; MTNP03_2; SGR01_1; SGR01_2; SGR13_1; SGR13_2; V25_1; V25_2; V27_1; V30_1; V30_2; V39_1
**6**	***camelopardalis*_ETH2_2**	**13**	ETH2_2; ETH3_2; MF01_2; MF02_2; MF04_1; MF04_2; MF06_2; MF07_2; MF11_2; MF14_2; MF17_2; ISC03_1; ISC03_2
**7**	***antiquorum*_GNP01_1**	**24**	GNP01_1; GNP02_1; GNP02_2; GNP03_1; GNP03_2; GNP04_1; GNP04_2; MF03_2; MF05_2; MF09_2; MF15_1; MF15_2; MF16_2; RET3_1; RET3_2; RET5_2; RETRot1_2; RETWil_2; ISC08_2; WA619_1; WA622_1; WA622_2;

			WA708_1; ZNP01_2
8	***peralta*_WA612_2**	2	WA612_2; WA619_2
9	***antiquorum*_GNP05_2**	1	GNP05_2
10	***giraffa*_V28_1**	1	V28_1
12	***giraffa*_BNP03_2**	35	BNP03_2; BNP06_2; CKGR02_2; CKGR05_1; CKGR05_2; CNP01_2; CNP02_2; ENP04_2; ENP07_1; ENP07_2; ENP08_2; ENP09_1; ENP09_2; ENP11_1; ENP11_2; ENP12_1; ENP12_2; ENP14_1; ENP14_2; ENP15_1; ENP15_2; ENP16_1; ENP16_2; ENP17_2; ENP18_1; ENP18_2; ENP19_2; ENP20_2; ENP21_2; MTNP02_2; V29_1; V29_2; V37_2; V38_1; V38_2
13	***giraffa*_V39_2**	1	V39_2
13	***giraffa*_V36_2**	1	V36_2
14	***giraffa*_V27_2**	1	V27_2
15	***giraffa*_BNP02_2**	1	BNP02_2
16	***giraffa*_SNNP_1**	1	SNNP_1
17	***giraffa*_KKR02_1**	1	KKR02_1
18	***giraffa*_KKR02_2**	1	KKR02_2
19	***giraffa*_BNP01_1**	45	BNP01_1; BNP01_2; BNP02_1; BNP04_1; BNP04_2; BNP05_1; BNP05_2; BNP09_1; BNP09_2; CKGR01_1; CKGR01_2; CKGR02_1; CKGR03_1; CKGR03_2; CNP03_1; CNP03_2; ENP21_1; KKR01_1; KKR01_2; KKR03_2; KKR04_1; KKR04_2; KKR05_2; KKR07_1; KKR07_2; KKR08_1; KKR08_2; SNNP_2; SUN1_1; SUN1_2; SUN2_1; SUN2_2; SUN3_1; SUN3_2; SUN4_1; SUN4_2; V23_1; V23_2; V24_1; V24_2; V26_1; V26_2; V28_2; V31_1; V31_2

Table K. Overview of the respective alleles comprised by the haplotypes shown in the network of *NOTCH2*.

n	Representative individual	∑	Included Alleles per Haplotypes
1	***camelopardalis*_BaNP4_1**	105	BaNP4_1; BaNP4_2; ETH1_1; ETH1_2; ETH2_1; ETH2_2; ETH3_1; ETH3_2; GNP01_1; GNP01_2; GNP02_1; GNP02_2; GNP03_1; GNP03_2; GNP04_1; GNP04_2; GNP05_1; GNP05_2; LVNP18_1; LVNP18_2; LVNP19_1; LVNP19_2; LVNP20_1; LVNP20_2; LVNP21_1; LVNP21_2; LVNP22_1; LVNP22_2; LVNP23a_1; LVNP23a_2; LVNP31_1; LVNP31_2; LVNP32_1; LVNP32_2; LVNP33_1; LVNP33_2; LVNP34_1; LVNP34_2; LVNP35_1; LVNP35_2; LVNP36_1; LVNP36_2; MF01_1; MF01_2; MF02_1; MF02_2; MF03_1; MF03_2; MF04_1; MF05_1; MF05_2; MF09_1; MF13_1; MF13_2; MF15_1; MF16_1; MF16_2; MF17_1; MF24_1; MF24_2; RET3_1; RET4_1; RET5_1; RET5_2; RET6_1; RETRot1_1; RETRot2_1; RETWil_1; ISC03_1; ISC04_1; ISC04_2; ISC08_1; ISC08_2; LWC01_1; SGR05_1; SGR05_2; SGR06_1; SGR06_2; SGR07_1; SGR07_2; SGR12_1; SGR12_2; SGR13_1; SGR13_2; SGR14_1; SGR14_2; SNR1_1; SNR2_1; SNR2_2; WA036_1; WA036_2; WA117_1; WA609_1; WA612_1; WA614_1; WA614_2; WA619_1; WA619_2; WA622_1; WA705_1; WA705_2; WA707_1; WA708_1; WA708_2; WA720_1

**2**	***rothschildi*_MF04_2**	**15**	MF04_2; MF06_1; MF06_2; MF07_1; MF07_2; MF09_2; MF11_1; MF11_2; MF14_1; MF14_2; MF15_2; MF17_2; SNR1_2; ZNP01_1; ZNP01_2
**3**	***peralta*_WA026_2**	**4**	WA026_2; WA612_2; WA623_2; WA700_2
**4**	***peralta*_WA117_2**	**1**	WA117_2
**5**	***peralta*_WA606_1**	**1**	WA606_1
**6**	***reticulata*_RET1_1**	**12**	RET1_1; RETRot3_1; RETWil2_1; ISC03_2; WA026_1; WA606_2; WA609_2; WA621_1; WA621_2; WA622_2; WA707_2; WA720_2
**7**	***reticulata*_RET1_2**	**8**	RET1_2; RET4_2; RET6_2; RETRot1_2; RETRot2_2; RETRot3_2; RETWil_2; RETWil2_2
**8**	***reticulata*_RET3_2**	**4**	RET3_2; LWC01_2; WA623_1; WA700_1
**9**	***tippelskirchi*_SGR01_1**	**1**	SGR01_1
**10**	***giraffa*_BNP01_1**	**123**	BNP01_1; BNP01_2; BNP02_1; BNP02_2; BNP03_1; BNP03_2; BNP04_1; BNP04_2; BNP05_1; BNP05_2; BNP06_1; BNP06_2; BNP09_1; BNP09_2; CKGR01_1; CKGR01_2; CKGR02_1; CKGR02_2; CKGR03_1; CKGR03_2; CKGR05_1; CKGR05_2; CNP01_1; CNP01_2; CNP02_1; CNP02_2; CNP03_1; CNP03_2; ENP04_1; ENP04_2; ENP07_1; ENP07_2; ENP08_1; ENP08_2; ENP09_1; ENP09_2; ENP11_1; ENP11_2; ENP12_1; ENP12_2; ENP14_1; ENP14_2; ENP15_1; ENP15_2; ENP16_1; ENP16_2; ENP17_1; ENP17_2; ENP18_1; ENP18_2; ENP19_1; ENP19_2; ENP20_1; ENP20_2; ENP21_1; ENP21_2; KKR01_1; KKR01_2; KKR02_1; KKR02_2; KKR03_1; KKR03_2; KKR04_1; KKR04_2; KKR05_1; KKR05_2; KKR07_1; KKR07_2; KKR08_1; KKR08_2; MGR01_1; MGR01_2; MGR02_1; MGR02_2; MGR03_1; MGR03_2; MGR04_1; MGR04_2; MGR05_1; MGR05_2; MTNP01_1; MTNP01_2; MTNP02_1; MTNP02_2; MTNP03_1; MTNP03_2; SGR01_2; SNNP_1; SNNP_2; SUN1_1; SUN1_2; SUN2_1; SUN2_2; SUN3_1; SUN3_2; SUN4_1; SUN4_2; V23_1; V23_2; V24_1; V24_2; V25_1; V25_2; V26_1; V26_2; V27_1; V27_2; V28_1; V28_2; V29_1; V29_2; V30_1; V30_2; V31_1; V31_2; V36_1; V36_2; V37_1; V37_2; V38_1; V38_2; V39_1; V39_2

Table L. Overview of the respective alleles comprised by the haplotypes shown in the network of *NUP155*.

n	Representative individual	∑	Included Alleles per Haplotypes
**1**	***camelopardalis*_BaNP4_1**	**2**	BaNP4_1; SNR1_1
**2**	***camelopardalis*_BaNP4_2**	**171**	BaNP4_2; BNP01_1; BNP01_2; BNP02_1; BNP02_2; BNP03_1; BNP03_2; BNP04_1; BNP04_2; BNP05_1; BNP05_2; BNP06_1; BNP06_2; BNP09_1; BNP09_2; CKGR01_1; CKGR01_2; CKGR02_1; CKGR02_2; CKGR03_1; CKGR03_2; CKGR05_1; CKGR05_2; CNP01_1; CNP01_2; CNP02_1; CNP02_2; CNP03_1; CNP03_2; ENP04_1; ENP04_2; ENP07_1; ENP07_2; ENP08_1; ENP08_2; ENP09_1; ENP09_2; ENP11_1; ENP11_2; ENP12_1; ENP12_2; ENP14_1; ENP14_2; ENP15_1; ENP15_2; ENP16_1; ENP16_2; ENP17_1; ENP17_2; ENP18_1; ENP18_2; ENP19_1; ENP19_2; ENP20_1; ENP20_2; ENP21_1; ENP21_2; ETH2_1; GNP02_1; GNP02_2; KKR01_1; KKR01_2; KKR02_1; KKR02_2; KKR03_1; KKR03_2; KKR04_1; KKR04_2; KKR05_1; KKR05_2; KKR07_1; KKR07_2; KKR08_1; KKR08_2; LVNP18_1;

			LVNP18_2; LVNP19_1; LVNP19_2; LVNP20_1; LVNP20_2; LVNP21_1; LVNP21_2; LVNP22_1; LVNP22_2; LVNP23a_1; LVNP23a_2; LVNP32_1; LVNP32_2; LVNP33_1; LVNP33_2; LVNP34_1; LVNP34_2; LVNP35_1; LVNP35_2; LVNP36_1; LVNP36_2; MGR01_1; MGR01_2; MGR02_1; MGR02_2; MGR03_1; MGR03_2; MGR04_1; MGR04_2; MGR05_1; MGR05_2; MTNP01_1; MTNP01_2; MTNP02_1; MTNP02_2; MTNP03_1; MTNP03_2; RET1_1; RET1_2; RET5_1; RET6_1; RETWil_1; ISC03_1; ISC03_2; ISC08_1; ISC08_2; LWC01_1; SGR01_1; SGR01_2; SGR05_1; SGR06_1; SGR06_2; SGR07_1; SGR07_2; SGR12_1; SGR12_2; SGR13_1; SGR13_2; SGR14_1; SGR14_2; SNNP_1; SNNP_2; SNR1_2; SUN1_1; SUN1_2; SUN2_1; SUN3_1; SUN3_2; SUN4_1; SUN4_2; V23_1; V23_2; V24_1; V24_2; V25_1; V25_2; V26_1; V26_2; V27_1; V27_2; V28_1; V28_2; V29_1; V29_2; V30_1; V30_2; V31_1; V31_2; V36_1; V36_2; V37_1; V37_2; V38_1; V38_2; V39_1; V39_2
**3**	***camelopardalis*_ETH1_1**	**2**	ETH1_1; ETH3_1
**4**	***camelopardalis*_ETH1_2**	**2**	ETH1_2; ETH3_2
**5**	***camelopardalis*_ETH2_2**	**1**	ETH2_2
**6**	***peralta*_WA036_1**	**25**	WA036_1; WA117_2; WA606_1; WA606_2; WA609_1; WA609_2; WA612_1; WA612_2; WA614_1; WA614_2; WA619_1; WA621_1; WA621_2; WA622_1; WA622_2; WA623_1; WA623_2; WA700_1; WA700_2; WA705_1; WA705_2; WA707_2; WA708_1; WA720_1; WA720_2
**7**	***peralta*_WA036_2**	**3**	WA036_2; WA619_2; WA708_2
**8**	***antiquorum*_GNP01_1**	**16**	GNP01_1; GNP03_1; GNP05_1; RET3_1; RETRot1_1; RETRot1_2; RETRot2_1; RETRot2_2; RETRot3_2; RETWil_2; ISC04_1; ISC04_2; LWC01_2; SNR2_1; ZNP01_1; ZNP01_2
**9**	***antiquorum*_GNP01_2**	**13**	GNP01_2; GNP03_2; GNP04_1; GNP04_2; GNP05_2; MF01_1; MF03_1; MF09_1; MF09_2; MF17_1; MF24_1; RETWil2_1; SNR2_2
**10**	***rothschildi*_MF01_2**	**28**	MF01_2; MF02_1; MF02_2; MF03_2; MF04_1; MF04_2; MF05_1; MF05_2; MF06_1; MF06_2; MF07_1; MF07_2; MF11_1; MF11_2; MF13_1; MF13_2; MF14_1; MF14_2; MF15_1; MF15_2; MF17_2; MF24_2; RET3_2; RETWil2_2; WA026_1; WA026_2; WA117_1; WA707_1
**11**	***reticulata*_RET4_1**	**1**	RET4_1
**12**	***reticulata*_RET4_2**	**1**	RET4_2
**13**	***reticulata*_RET5_2**	**1**	RET5_2
**14**	***reticulata*_RET6_2**	**2**	RET6_2; RETRot3_1
**15**	***thornicrofti*_LVNP31_1**	**2**	LVNP31_1; LVNP31_2
**16**	***tippelskirchi*_SGR05_2**	**1**	SGR05_2
**17**	***giraffa*_SUN_2**	**1**	SUN_2

Table M. Overview of the respective alleles comprised by the haplotypes shown in the network of *OTOF*.

n	Representative individual	∑	Included Alleles per Haplotypes
1	*camelopardalis*_BaNP4_1	37	BaNP4_1; ETH1_1; ETH1_2; GNP01_1; GNP02_1; GNP02_2; GNP03_1; GNP04_1; GNP04_2; GNP05_1; GNP05_2; MF01_1; MF01_2; MF02_1; MF03_1; MF05_1; MF06_1; MF09_1; MF11_1; MF13_1; MF13_2; MF14_1; MF14_2; MF15_1; MF16_1; MF16_2; MF17_2; MF24_1; MF24_2; RETWil2_1; SNR1_1; SNR1_2; SNR2_1; SNR2_2; WA036_1; WA606_1; WA708_1
2	*camelopardalis*_BaNP4_2	4	BaNP4_2; WA026_2; WA705_1; WA720_1
3	*camelopardalis*_ETH2_1	26	ETH2_1; ETH2_2; ETH3_1; ETH3_2; GNP01_2; GNP03_2; MF02_2; MF03_2; MF04_1; MF04_2; MF06_2; MF07_1; MF07_2; MF09_2; MF11_2; MF15_2; RET1_1; RET1_2; RET3_2; RET4_2; RET6_2; RETRot1_2; RETRot2_2; RETRot3_1; RETRot3_2; ISC04_1; ISC04_2
4	*antiquorum*_ZNP01_1	2	ZNP01_1; ZNP01_2
5	*peralta*_WA026_1	10	WA026_1; WA036_2; WA117_2; WA612_2; WA621_1; WA621_2; WA623_1; WA623_2; WA707_1; WA707_2
6	*peralta*_WA117_1	4	WA117_1; WA614_1; WA622_1; WA700_1
7	*peralta*_WA606_2	6	WA606_2; WA609_1; WA612_1; WA614_2; WA619_2; WA708_2
8	*peralta*_WA609_2	1	WA609_2
9	*peralta*_WA622_2	1	WA622_2
10	*peralta*_WA626_1	1	WA626_1
11	*peralta*_WA705_2	2	WA705_2; WA720_2
12	*reticulata*_LWC01_2	1	LWC01_2
13	*rothschildi*_MF05_2	1	MF05_2
14	*rothschildi*_MF17_1	1	MF17_1
15	*reticulata*_RET3_1	9	RET3_1; RET5_2; RET6_1; RETRot1_1; RETWil_2; RETWil2_2; ISC03_1; ISC03_2; ISC08_2
16	*thornicrofti*_LVNP31_1	8	LVNP31_1; LVNP31_2; SGR01_2; SGR07_2; SGR12_1; SGR12_2; SGR13_1; SGR13_2
17	*thornicrofti*_LVNP35_1	2	LVNP35_1; LVNP35_2
18	*giraffa*_BNP01_1	95	BNP01_1; BNP01_2; BNP02_1; BNP03_1; BNP03_2; BNP04_1; BNP05_1; BNP05_2; BNP06_1; BNP06_2; BNP09_1; BNP09_2; CKGR03_1; CKGR03_2; CKGR05_1; CNP01_1; CNP01_2; CNP02_1; CNP03_1; CNP03_2; ENP04_1; ENP07_1; ENP08_1; ENP09_1; ENP11_1; ENP11_2; ENP12_1; ENP12_2; ENP14_1; ENP14_2; ENP15_1; ENP15_2; ENP16_1; ENP16_2; ENP17_1; ENP17_2; ENP18_1; ENP18_2; ENP19_1; ENP19_2; ENP20_1; ENP21_1; ENP21_2; KKR01_1; KKR01_2; KKR02_1; KKR02_2; KKR03_1; KKR04_1; KKR04_2; KKR05_1; KKR05_2; KKR07_1; KKR08_1; KKR08_2; MGR01_1; MGR01_2; MGR02_1; MGR02_2; MGR03_1; MGR03_2; MGR04_1; MGR05_1; MGR05_2; MTNP02_1; MTNP02_2; SNNP_1; SNNP_2; SUN1_1; SUN1_2; SUN2_1; SUN2_2; SUN3_1; SUN3_2; SUN4_1; SUN4_2; V23_1; V23_2; V24_1; V25_1; V26_1; V27_1; V27_2; V28_1; V28_2; V29_1; V30_1; V31_1; V36_1; V36_2; V37_1; V37_2; V38_1; V38_2; V39_1; V39_2
19	*giraffa*_BNP02_2	59	BNP02_2; BNP04_2; CKGR01_1; CKGR01_2; CKGR02_1; CKGR02_2; CKGR05_2; CNP02_2; ENP04_2; ENP07_2; ENP08_2; ENP09_2; ENP20_2; KKR07_2; LVNP18_1; LVNP18_2; LVNP19_1; LVNP19_2; LVNP20_1; LVNP20_2; LVNP21_1; LVNP21_2; LVNP22_1; LVNP22_2; LVNP23a_1; LVNP23a_2; LVNP32_1; LVNP32_2; LVNP34_1; LVNP34_2; LVNP36_1; LVNP36_2; MGR04_2; MTNP01_1; MTNP01_2; MTNP03_1; MTNP03_2; RET4_1; RET5_1; RETRot2_1; RETWil_1; ISC08_1; LWC01_1; SGR01_1;

	SGR05_1; SGR05_2; SGR06_1; SGR06_2; SGR07_1; SGR14_1; SGR14_2; V24_2; V25_2; V26_2; V29_2; V30_2; V31_2; WA619_1; WA700_2

Table N. Overview of the respective alleles comprised by the haplotypes shown in the network of *PLCE1*.

n	Representative individual	∑	Included Alleles per Haplotypes
**1**	***camelopardalis*_BaNP4_1**	**110**	BaNP4_1; BaNP4_2; CKGR01_1; CKGR01_2; ETH1_1; ETH1_2; ETH2_1; ETH2_2; ETH3_1; ETH3_2; GNP01_1; GNP01_2; GNP02_1; GNP02_2; GNP03_1; GNP03_2; GNP04_1; GNP04_2; GNP05_1; GNP05_2; MF01_1; MF01_2; MF02_1; MF03_1; MF03_2; MF04_1; MF05_1; MF05_2; MF07_1; MF11_1; MF11_2; MF13_1; MF13_2; MF14_1; MF14_2; MF15_1; MF16_1; MF16_2; MF17_1; MF24_1; MF24_2; RET1_1; RET1_2; RET3_1; RET3_2; RET4_1; RET4_2; RET5_1; RET5_2; RET6_1; RET6_2; RETRot1_1; RETRot1_2; RETRot2_1; RETRot2_2; RETRot3_1; RETRot3_2; RETWil_1; RETWil_2; RETWil2_1; RETWil2_2; ISC03_1; ISC03_2; ISC04_1; ISC04_2; ISC08_1; ISC08_2; LWC01_1; LWC01_2; SNR1_1; SNR1_2; SNR2_1; SNR2_2; V30_1; WA026_1; WA026_2; WA036_1; WA036_2; WA117_1; WA117_2; WA606_1; WA606_2; WA609_1; WA609_2; WA612_1; WA612_2; WA614_1; WA614_2; WA619_1; WA619_2; WA621_1; WA621_2; WA622_1; WA622_2; WA623_1; WA623_2; WA700_1; WA700_2; WA705_1; WA705_2; WA707_1; WA707_2; WA708_1; WA708_2; WA720_1; WA720_2; ZNP01_1; ZNP01_2
**2**	***giraffa*_MGR02_1**	**1**	MGR02_1
**3**	***giraffa*_MGR02_2**	**13**	MGR02_2; SNNP_1; SNNP_2; V24_1; V26_2; V27_1; V27_2; V28_2; V30_2; V31_1; V31_2; V39_1; V39_2
**4**	***giraffa*_MGR05_1**	**1**	MGR05_1
**5**	***giraffa*_V36_1**	**1**	V36_1
**6**	***giraffa*_KKR05_2**	**1**	KKR05_2
**7**	***giraffa*_BNP01_1**	**13**	BNP01_1; BNP02_1; BNP04_1; BNP09_1; CNP01_1; CNP01_2; ENP15_2; ENP16_1; ENP16_2; ENP18_2; ENP19_2; MGR05_2; V37_2
**8**	***giraffa*_BNP01_2**	**50**	BNP01_2; BNP02_2; BNP03_1; BNP03_2; BNP04_2; BNP05_2; BNP06_1; BNP06_2; BNP09_2; CNP03_1; CNP03_2; ENP04_2; ENP07_2; ENP08_1; ENP08_2; ENP09_2; ENP21_2; KKR01_1; KKR01_2; KKR02_2; KKR03_1; KKR03_2; KKR04_2; KKR07_2; KKR08_1; KKR08_2; MF02_2; MF04_2; MF06_1; MF06_2; MF07_2; MF09_1; MF09_2; MF15_2; MF17_2; MGR01_1; MGR01_2; MGR03_2; MGR04_1; MGR04_2; MTNP01_2; MTNP02_2; MTNP03_2; V23_2; V24_2; V25_1; V25_2; V36_2; V38_1; V38_2
**9**	***giraffa*_BNP05_1**	**84**	BNP05_1; CKGR02_1; CKGR02_2; CKGR03_1; CKGR03_2; CKGR05_1; CKGR05_2; ENP04_1; ENP07_1; ENP09_1; ENP11_1; ENP11_2; ENP12_1; ENP12_2; ENP14_1; ENP14_2; ENP15_1; ENP17_1; ENP17_2; ENP18_1; ENP19_1; ENP20_1; ENP20_2; ENP21_1; KKR02_1; KKR04_1; KKR05_1; KKR07_1; LVNP18_1; LVNP18_2; LVNP19_1; LVNP19_2; LVNP20_1; LVNP20_2; LVNP21_1; LVNP21_2; LVNP22_1; LVNP22_2; LVNP23a_1; LVNP23a_2; LVNP31_1; LVNP31_2; LVNP32_1; LVNP32_2; LVNP33_1; LVNP33_2; LVNP34_1; LVNP34_2; LVNP35_1; LVNP35_2; LVNP36_1; LVNP36_2; MGR03_1; MTNP01_1; MTNP02_1; MTNP03_1; SGR01_1; SGR01_2; SGR05_1; SGR05_2; SGR06_1; SGR06_2; SGR07_1; SGR07_2; SGR12_1; SGR12_2; SGR13_1; SGR13_2; SGR14_1; SGR14_2; SUN1_1; SUN1_2; SUN2_1; SUN2_2; SUN3_1; SUN3_2; SUN4_1; SUN4_2; V23_1; V26_1; V28_1; V29_1; V29_2; V37_1

Table O. Overview of the respective alleles comprised by the haplotypes shown in the network of *RASSF4*.

n	Representative individual	∑	Included Alleles per Haplotypes
1	*antiquorum*_GNP01_1	8	GNP01_1; GNP01_2; GNP03_1; GNP03_2; GNP04_1; GNP04_2; SNR2_1; SNR2_2
2	*angolensis*_CKGR02_2	24	CKGR02_2; CNP01_2; GNP02_2; GNP05_1; GNP05_2; SNR1_1; SNR1_2; WA026_1; WA026_2; WA036_1; WA036_2; WA606_1; WA606_2; WA609_1; WA609_2; WA623_1; WA623_2; WA700_1; WA700_2; WA707_1; WA708_1; WA708_2; ZNP01_1; ZNP01_2
3	*camelopardalis*_ETH1_1	40	ETH1_1; ETH1_2; LVNP18_1; LVNP18_2; LVNP19_1; LVNP19_2; LVNP20_1; LVNP20_2; LVNP21_1; LVNP21_2; LVNP22_1; LVNP22_2; LVNP23a_1; LVNP23a_2; LVNP31_1; LVNP31_2; LVNP33_1; LVNP33_2; LVNP34_1; LVNP34_2; LVNP35_1; LVNP35_2; LVNP36_1; LVNP36_2; RET6_2; RETRot1_1; SGR01_1; SGR01_2; SGR05_1; SGR05_2; SGR06_1; SGR06_2; SGR07_1; SGR07_2; SGR12_1; SGR12_2; SGR13_1; SGR13_2; SGR14_1; SGR14_2
4	*camelopardalis*_ETH2_1	22	ETH2_1; ETH2_2; ETH3_1; MF01_1; MF03_1; MF03_2; MF04_1; MF05_1; MF07_1; MF09_1; MF09_2; MF13_1; MF13_2; MF14_1; MF15_1; MF16_1; MF16_2; MF24_1; WA619_1; WA619_2; WA621_1; WA622_1
5	*camelopardalis*_ETH3_2	5	ETH3_2; MF02_1; MF02_2; MF06_1; MF06_2
6	*rothschildi*_MF01_2	1	MF01_2
7	*rothschildi*_MF04_2	3	MF04_2; MF05_2; MF07_2
8	*rothschildi*_MF11_1	1	MF11_1
9	*rothschildi*_MF11_2	2	MF11_2; MF17_1
10	*rothschildi*_MF14_2	1	MF14_2
11	*rothschildi*_MF15_2	2	MF15_2; MF24_2
12	*rothschildi*_MF17_2	1	MF17_2
13	*peralta*_WA614_1	4	WA614_1; WA614_2; WA622_2; WA705_1
14	*peralta*_WA621_2	1	WA621_2
15	*peralta*_WA705_2	1	WA705_2
16	*peralta*_WA707_2	1	WA707_2
17	*peralta*_WA720_1	2	WA720_1; WA720_2
18	*reticulata*_RET1_2	1	RET1_2
19	*reticulata*_RET3_2	1	RET3_2
20	*reticulata*_RET4_1	2	RET4_1; RETRot2_1
21	*reticulata*_RET4_2	1	RET4_2
22	*reticulata*_RET5_1	1	RET5_1
23	*reticulata*_RET5_2	1	RET5_2
24	*reticulata*_RET6_1	8	RET6_1; RETRot1_2; RETRot3_1; RETWil2_1; ISC03_1; ISC03_2; ISC04_1; ISC08_1

**25**	***reticulata*_RETRot2_2**	**3**	RETRot2_2; RETRot3_2; RETWil2_2
**26**	***reticulata*_RETWil_1**	**1**	RETWil_1
**27**	***reticulata*_RETWil_2**	**1**	RETWil_2
**28**	***reticulata*_ISC04_2**	**2**	ISC04_2; ISC08_2
**29**	***reticulata*_LWC01_1**	**2**	LWC01_1; LWC01_2
**30**	***giraffa*_MGR01_1**	**3**	MGR01_1; MGR02_1; RET3_1
**31**	***giraffa*_MGR01_2**	**2**	MGR01_2; MGR02_2
**32**	***giraffa*_BNP01_1**	**118**	BNP01_1; BNP01_2; BNP02_1; BNP02_2; BNP03_1; BNP03_2; BNP04_1; BNP04_2; BNP05_1; BNP05_2; BNP06_1; BNP06_2; BNP09_1; BNP09_2; CKGR01_1; CKGR01_2; CKGR02_1; CKGR03_1; CKGR03_2; CKGR05_1; CKGR05_2; CNP01_1; CNP02_1; CNP02_2; CNP03_1; CNP03_2; ENP04_1; ENP04_2; ENP07_1; ENP07_2; ENP08_1; ENP08_2; ENP09_1; ENP09_2; ENP11_1; ENP11_2; ENP12_1; ENP12_2; ENP14_1; ENP14_2; ENP15_1; ENP15_2; ENP16_1; ENP16_2; ENP17_1; ENP17_2; ENP18_1; ENP18_2; ENP19_1; ENP19_2; ENP20_1; ENP20_2; ENP21_1; ENP21_2; GNP02_1; KKR01_1; KKR01_2; KKR02_1; KKR02_2; KKR03_1; KKR03_2; KKR04_1; KKR04_2; KKR05_1; KKR05_2; KKR07_1; KKR07_2; KKR08_1; KKR08_2; MGR03_1; MGR03_2; MGR04_1; MGR04_2; MGR05_1; MGR05_2; MTNP01_1; MTNP01_2; MTNP02_1; MTNP02_2; MTNP03_1; MTNP03_2; RET1_1; SNNP_1; SNNP_2; SUN1_1; SUN1_2; SUN2_1; SUN2_2; SUN3_1; SUN3_2; SUN4_1; SUN4_2; V23_1; V23_2; V24_1; V24_2; V25_1; V25_2; V26_1; V26_2; V27_1; V27_2; V28_1; V28_2; V29_1; V29_2; V30_1; V30_2; V31_1; V31_2; V36_1; V36_2; V37_1; V37_2; V38_1; V38_2; V39_1; V39_2

Table P. Overview of the respective alleles comprised by the haplotypes shown in the network of *RFC5*.

n	Representative individual	∑	Included Alleles per Haplotypes
**1**	***camelopardalis*_BaNP4_1**	**76**	BaNP4_1; ETH1_1; ETH1_2; ETH2_1; ETH3_1; ETH3_2; GNP01_1; GNP01_2; GNP03_1; GNP03_2; GNP04_1; GNP04_2; GNP05_1; MF01_1; MF01_2; MF02_1; MF02_2; MF03_1; MF03_2; MF04_1; MF04_2; MF05_1; MF05_2; MF06_1; MF06_2; MF07_1; MF07_2; MF09_1; MF09_2; MF11_1; MF11_2; MF13_1; MF13_2; MF14_1; MF14_2; MF15_1; MF15_2; MF16_1; MF16_2; MF17_1; MF17_2; MF24_1; MF24_2; RET1_1; RET4_1; RET6_1; RETWil2_1; ISC04_1; LWC01_1; LWC01_2; SNR1_1; SNR1_2; SNR2_1; SNR2_2; WA026_1; WA026_2; WA036_1; WA117_1; WA606_1; WA606_2; WA609_1; WA612_1; WA614_1; WA614_2; WA619_1; WA619_2; WA621_1; WA622_1; WA623_1; WA623_2; WA700_1; WA705_1; WA707_1; WA720_1; WA720_2; ZNP01_1
**2**	***camelopardalis*_BaNP4_2**	**2**	BaNP4_2; ETH2_2
**3**	***antiquorum*_GNP02_1**	**4**	GNP02_1; GNP02_2; GNP05_2; ZNP01_2
**4**	***peralta*_WA036_2**	**11**	WA036_2; WA117_2; WA609_2; WA612_2; WA621_2; WA622_2; WA700_2; WA705_2; WA707_2; WA708_1; WA708_2
**5**	***reticulata*_RET1_2**	**14**	RET1_2; RET3_2; RET4_2; RET5_1; RET5_2; RET6_2; RETRot1_1; RETRot1_2; RETWil_2; RETWil2_2; ISC03_1; ISC03_2; ISC04_2; ISC08_2

6	*reticulata*_RET3_1	3	RET3_1; RETWil_1; ISC08_1
7	*reticulata*_RETRot2_1	1	RETRot2_1
8	*reticulata*_RETRot2_2	1	RETRot2_2
9	*reticulata*_RETRot3_1	1	RETRot3_1
10	*reticulata*_RETRot3_2	1	RETRot3_2
11	*thornicrofti*_LVNP18_1	38	LVNP18_1; LVNP18_2; LVNP19_1; LVNP19_2; LVNP20_1; LVNP20_2; LVNP21_1; LVNP21_2; LVNP22_1; LVNP22_2; LVNP23a_1; LVNP23a_2; LVNP31_1; LVNP31_2; LVNP32_1; LVNP32_2; LVNP33_1; LVNP33_2; LVNP34_1; LVNP34_2; LVNP35_1; LVNP35_2; LVNP36_1; LVNP36_2; SGR01_1; SGR01_2; SGR05_1; SGR05_2; SGR06_1; SGR06_2; SGR07_1; SGR07_2; SGR12_1; SGR12_2; SGR13_1; SGR13_2; SGR14_1; SGR14_2
12	*angolensis*_ENP04_1	1	ENP04_1
13	*angolensis*_ENP08_1	2	ENP08_1; ENP12_1
14	*angolensis*_ENP08_2	9	ENP08_2; ENP11_1; ENP11_2; ENP14_1; ENP15_1; ENP20_1; ENP20_2; ENP21_1; ENP21_2
15	*angolensis*_ENP12_2	2	ENP12_2; ENP15_2
16	*angolensis*_ENP17_1	2	ENP17_1; ENP18_1
17	*angolensis*_ENP17_2	1	ENP17_2
18	*giraffa*_KKR05_1	2	KKR05_1; KKR05_2
19	*giraffa*_BNP01_1	103	BNP01_1; BNP01_2; BNP02_1; BNP02_2; BNP03_1; BNP03_2; BNP04_1; BNP04_2; BNP05_1; BNP05_2; BNP06_1; BNP06_2; BNP09_1; BNP09_2; CKGR01_1; CKGR01_2; CKGR02_1; CKGR02_2; CKGR03_1; CKGR03_2; CKGR05_1; CKGR05_2; CNP01_1; CNP01_2; CNP02_1; CNP02_2; CNP03_1; CNP03_2; ENP04_2; ENP07_1; ENP07_2; ENP09_1; ENP09_2; ENP14_2; ENP16_1; ENP16_2; ENP18_2; ENP19_1; ENP19_2; KKR01_1; KKR01_2; KKR02_1; KKR02_2; KKR03_1; KKR03_2; KKR04_1; KKR04_2; KKR07_1; KKR07_2; KKR08_1; KKR08_2; MGR01_1; MGR01_2; MGR02_1; MGR02_2; MGR03_1; MGR03_2; MGR04_1; MGR04_2; MGR05_1; MGR05_2; MTNP01_1; MTNP01_2; MTNP02_1; MTNP02_2; MTNP03_1; MTNP03_2; SNNP_1; SNNP_2; SUN1_1; SUN1_2; SUN2_1; SUN2_2; SUN3_1; SUN3_2; SUN4_1; SUN4_2; V23_1; V23_2; V24_1; V24_2; V25_1; V25_2; V26_1; V26_2; V27_1; V27_2; V28_1; V28_2; V29_1; V29_2; V30_1; V30_2; V31_1; V31_2; V36_1; V36_2; V37_1; V37_2; V38_1; V38_2; V39_1; V39_2

Table Q. Overview of the respective alleles comprised by the haplotypes shown in the network of *SAP130*.

n	Representative individual	∑	Included Alleles per Haplotypes
1	*antiquorum*_SNR2_1	2	SNR2_1; SNR2_2
2	*peralta*_WA036_2	3	WA036_2; WA612_2; WA622_2

**3**	***camelopardalis*_BaNP4_1**	**86**	BaNP4_1; BaNP4_2; ETH1_1; ETH1_2; ETH2_1; ETH2_2; ETH3_1; ETH3_2; GNP01_1; GNP01_2; GNP02_1; GNP02_2; GNP03_1; GNP03_2; GNP04_1; GNP04_2; GNP05_1; GNP05_2; MF01_1; MF01_2; MF02_1; MF02_2; MF03_1; MF03_2; MF04_1; MF04_2; MF05_1; MF05_2; MF06_1; MF06_2; MF07_1; MF07_2; MF09_1; MF09_2; MF11_1; MF11_2; MF13_1; MF13_2; MF14_1; MF14_2; MF15_1; MF15_2; MF16_1; MF16_2; MF17_1; MF17_2; MF24_1; MF24_2; RET1_1; RET3_1; RET3_2; RET4_1; RET4_2; RETWil2_1; ISC03_1; LWC01_1; SNR1_1; SNR1_2; WA026_1; WA026_2; WA036_1; WA117_1; WA117_2; WA606_1; WA609_1; WA609_2; WA612_1; WA614_1; WA614_2; WA619_1; WA619_2; WA622_1; WA623_1; WA623_2; WA700_1; WA700_2; WA705_1; WA705_2; WA707_1; WA707_2; WA708_1; WA708_2; WA720_1; WA720_2; ZNP01_1; ZNP01_2
**4**	***peralta*_WA606_2**	**3**	WA606_2; WA621_1; WA621_2
**5**	***reticulata*_RET1_2**	**12**	RET1_2; RET6_2; RETRot1_2; RETRot2_1; RETRot2_2; RETRot3_2; RETWil_1; RETWil_2; ISC04_1; ISC04_2; ISC08_1; ISC08_2
6	***reticulata*_RET5_1**	**1**	RET5_1
**7**	***reticulata*_RET5_2**	**5**	RET5_2; RET6_1; RETRot1_1; RETRot3_1; LWC01_2
**8**	***reticulata*_RETWil2_2**	**1**	RETWil2_2
**9**	***reticulata*_ISC03_2**	**1**	ISC03_2
**10**	***thornicrofti*_LVNP18_1**	**27**	LVNP18_1; LVNP18_2; LVNP19_1; LVNP19_2; LVNP20_1; LVNP20_2; LVNP21_1; LVNP21_2; LVNP22_1; LVNP22_2; LVNP23a_1; LVNP23a_2; LVNP31_1; LVNP31_2; LVNP32_1; LVNP32_2; LVNP33_1; LVNP33_2; LVNP34_1; LVNP34_2; LVNP35_1; LVNP35_2; LVNP36_1; LVNP36_2; SGR05_1; SGR06_1; SGR14_1
**11**	***angolensis*_CKGR03_1**	**2**	CKGR03_1; CKGR03_2
**12**	***angolensis*_ENP04_2**	**1**	ENP04_2
**13**	***giraffa*_BNP01_1**	**130**	BNP01_1; BNP01_2; BNP02_1; BNP02_2; BNP03_1; BNP03_2; BNP04_1; BNP04_2; BNP05_1; BNP05_2; BNP06_1; BNP06_2; BNP09_1; BNP09_2; CKGR01_1; CKGR01_2; CKGR02_1; CKGR02_2; CKGR05_1; CKGR05_2; CNP01_1; CNP01_2; CNP02_1; CNP02_2; CNP03_1; CNP03_2; ENP04_1; ENP07_1; ENP07_2; ENP08_1; ENP08_2; ENP09_1; ENP09_2; ENP11_1; ENP11_2; ENP12_1; ENP12_2; ENP14_1; ENP14_2; ENP15_1; ENP15_2; ENP16_1; ENP16_2; ENP17_1; ENP17_2; ENP18_1; ENP18_2; ENP19_1; ENP19_2; ENP20_1; ENP20_2; ENP21_1; ENP21_2; KKR01_1; KKR01_2; KKR02_1; KKR02_2; KKR03_1; KKR03_2; KKR04_1; KKR04_2; KKR05_1; KKR05_2; KKR07_1; KKR07_2; KKR08_1; KKR08_2; MGR01_1; MGR01_2; MGR02_1; MGR02_2; MGR03_1; MGR03_2; MGR04_1; MGR04_2; MGR05_1; MGR05_2; MTNP01_1; MTNP01_2; MTNP02_1; MTNP02_2; MTNP03_1; MTNP03_2; SGR01_1; SGR01_2; SGR05_2; SGR06_2; SGR07_1; SGR07_2; SGR12_1; SGR12_2; SGR13_1; SGR13_2; SGR14_2; SNNP_1; SNNP_2; SUN1_1; SUN1_2; SUN2_1; SUN2_2; SUN3_1; SUN3_2; SUN4_1; SUN4_2; V23_1; V23_2; V24_1; V24_2; V25_1; V25_2; V26_1; V26_2; V27_1; V27_2; V28_1; V28_2; V29_1; V29_2; V30_1; V30_2; V31_1; V31_2; V36_1; V36_2; V37_1; V37_2; V38_1; V38_2; V39_1; V39_2

Table R. Overview of the respective alleles comprised by the haplotypes shown in the network of *SOS1*.

n	Representative individual	∑	Included Alleles per Haplotypes
**1**	***camelopardalis*_BaNP4_1**	**96**	BaNP4_1; BaNP4_2; ETH1_1; ETH1_2; ETH2_1; ETH2_2; ETH3_1; ETH3_2; GNP01_1; GNP01_2; GNP02_1; GNP02_2; GNP03_1; GNP03_2; GNP04_1; GNP04_2; GNP05_1; GNP05_2; MF01_1; MF01_2; MF02_1; MF02_2; MF03_1; MF03_2; MF04_1; MF04_2; MF05_1; MF05_2; MF06_1; MF06_2; MF07_1; MF07_2; MF09_1; MF09_2; MF11_1; MF11_2; MF13_1; MF13_2; MF14_1; MF14_2; MF15_1; MF15_2; MF16_1; MF16_2; MF17_1; MF17_2; MF24_1; MF24_2; RET1_1; RET1_2; RET3_1; RET3_2; RET4_1; RET4_2; RET5_1; RET6_1; RETRot1_1; RETRot2_1; RETRot2_2; RETRot3_1; RETRot3_2; RETWil_1; RETWil2_1; RETWil2_2; ISC04_1; ISC04_2; ISC08_1; ISC08_2; LWC01_1; LWC01_2; SNR1_1; SNR1_2; SNR2_1; SNR2_2; WA026_1; WA026_2; WA036_1; WA036_2; WA621_1; WA621_2; WA622_1; WA622_2; WA623_1; WA623_2; WA700_1; WA700_2; WA705_1; WA705_2; WA707_1; WA707_2; WA708_1; WA708_2; WA720_1; WA720_2; ZNP01_1; ZNP01_2
**2**	***reticulata*_RET5_2**	**4**	RET5_2; RET6_2; RETRot1_2; RETWil_2
**3**	***reticulata*_ISC03_1**	**2**	ISC03_1; ISC03_2
**4**	***thornicrofti*_LVNP32_1**	**2**	LVNP32_1; LVNP32_2
**5**	***giraffa*_KKR03_2**	**38**	KKR03_2; LVNP18_1; LVNP18_2; LVNP19_1; LVNP19_2; LVNP20_1; LVNP20_2; LVNP21_1; LVNP21_2; LVNP22_1; LVNP22_2; LVNP23a_1; LVNP23a_2; LVNP31_1; LVNP31_2; LVNP33_1; LVNP33_2; LVNP34_1; LVNP34_2; LVNP35_1; LVNP35_2; LVNP36_1; LVNP36_2; MGR03_1; SGR01_1; SGR01_2; SGR05_1; SGR05_2; SGR06_1; SGR06_2; SGR07_1; SGR07_2; SGR12_1; SGR12_2; SGR13_1; SGR13_2; SGR14_1; SGR14_2
**6**	***giraffa*_MGR04_2**	**1**	MGR04_2
**7**	***giraffa*_BNP01_1**	**28**	BNP01_1; BNP01_2; BNP03_1; BNP04_1; BNP04_2; BNP05_1; BNP06_1; BNP09_1; BNP09_2; CKGR05_1; CKGR05_2; KKR01_1; KKR01_2; KKR03_1; MGR01_1; MGR01_2; MGR02_1; MGR04_1; MTNP01_1; MTNP01_2; MTNP02_1; MTNP03_1; MTNP03_2; V29_1; V31_1; V36_1; V37_1; V38_1
**8**	***giraffa*_BNP02_1**	**91**	BNP02_1; BNP02_2; BNP03_2; BNP05_2; BNP06_2; CKGR01_1; CKGR01_2; CKGR02_1; CKGR02_2; CKGR03_1; CKGR03_2; CNP01_1; CNP01_2; CNP02_1; CNP02_2; CNP03_1; CNP03_2; ENP04_1; ENP04_2; ENP07_1; ENP07_2; ENP08_1; ENP08_2; ENP09_1; ENP09_2; ENP11_1; ENP11_2; ENP12_1; ENP12_2; ENP14_1; ENP14_2; ENP15_1; ENP15_2; ENP16_1; ENP16_2; ENP17_1; ENP17_2; ENP18_1; ENP18_2; ENP19_1; ENP19_2; ENP20_1; ENP20_2; ENP21_1; ENP21_2; KKR02_1; KKR02_2; KKR04_1; KKR04_2; KKR05_1; KKR05_2; KKR07_1; KKR07_2; KKR08_1; KKR08_2; MGR02_2; MGR03_2; MGR05_1; MGR05_2; MTNP02_2; SNNP_1; SNNP_2; SUN1_1; SUN1_2; SUN2_1; SUN2_2; SUN3_1; SUN3_2; SUN4_1; SUN4_2; V23_1; V23_2; V24_1; V24_2; V25_1; V25_2; V26_1; V26_2; V27_1; V27_2; V28_1; V28_2; V29_2; V30_1; V30_2; V31_2; V36_2; V37_2; V38_2; V39_1; V39_2

Table S. Overview of the respective alleles comprised by the haplotypes shown in the network of *UBN2*.

n	Representative individual	∑	Included Alleles per Haplotypes
**1**	***camelopardalis*_BaNP4_1**	**42**	BaNP4_1; BaNP4_2; ETH1_2; ETH2_1; ETH3_1; GNP01_1; GNP01_2; GNP02_2; GNP04_1; GNP04_2; GNP05_2; MF01_1; MF03_2; MF05_1; MF07_1; MF11_1; MF11_2; MF15_1; MF16_1; MF16_2; MTNP03_1; MTNP03_2; RET1_1; RET1_2; RET3_2; RET5_1; RET5_2; RET6_1; RET6_2; RETRot3_1; SNNP_2; SNR1_1; SNR1_2; SNR2_1; SNR2_2; WA036_1; WA036_2; WA612_2; WA614_2; WA708_1; ZNP01_1; ZNP01_2
**2**	***reticulata*_RET4_1**	**14**	RET4_1; RET4_2; RETRot1_1; RETRot1_2; RETRot2_1; RETRot2_2; RETWil2_1; ISC03_1; ISC03_2; ISC04_1; ISC04_2; ISC08_1; LWC01_1; LWC01_2
**3**	***reticulata*_RETWil2_2**	**1**	RETWil2_2
**4**	***reticulata*_ISC08_2**	**1**	ISC08_2
**5**	***thornicrofti*_LVNP18_1**	**38**	LVNP18_1; LVNP18_2; LVNP19_1; LVNP19_2; LVNP20_1; LVNP20_2; LVNP21_1; LVNP21_2; LVNP22_1; LVNP22_2; LVNP23a_1; LVNP23a_2; LVNP31_1; LVNP31_2; LVNP32_1; LVNP32_2; LVNP33_1; LVNP33_2; LVNP34_1; LVNP34_2; LVNP35_1; LVNP35_2; LVNP36_1; LVNP36_2; SGR01_1; SGR01_2; SGR05_1; SGR05_2; SGR06_1; SGR06_2; SGR07_1; SGR07_2; SGR12_1; SGR12_2; SGR13_1; SGR13_2; SGR14_1; SGR14_2
**6**	***giraffa*_V25_2**	**1**	V25_2
**7**	***giraffa*_BNP01_1**	**131**	BNP01_1; BNP01_2; BNP03_1; BNP04_1; BNP04_2; BNP06_1; BNP09_1; BNP09_2; CKGR01_1; CKGR01_2; CKGR02_1; CKGR02_2; CKGR03_1; CKGR05_1; CKGR05_2; CNP03_1; CNP03_2; ENP04_1; ENP07_1; ENP07_2; ENP08_1; ENP09_1; ENP09_2; ENP11_1; ENP12_1; ENP12_2; ENP14_1; ENP15_1; ENP15_2; ENP16_1; ENP16_2; ENP17_1; ENP17_2; ENP18_1; ENP18_2; ENP19_1; ENP19_2; ENP20_1; ENP21_1; ENP21_2; ETH1_1; ETH2_2; ETH3_2; GNP02_1; GNP03_1; GNP03_2; GNP05_1; KKR01_1; KKR01_2; KKR02_1; KKR02_2; KKR03_1; KKR03_2; KKR04_1; KKR04_2; KKR05_1; KKR05_2; KKR07_1; KKR07_2; KKR08_1; KKR08_2; MF01_2; MF02_1; MF02_2; MF03_1; MF04_1; MF04_2; MF05_2; MF06_1; MF06_2; MF07_2; MF09_1; MF09_2; MF13_1; MF13_2; MF14_1; MF14_2; MF15_2; MF17_1; MF17_2; MF24_1; MF24_2; MGR01_1; MGR01_2; MGR03_1; MTNP01_1; MTNP01_2; MTNP02_1; MTNP02_2; RET3_1; RETRot3_2; RETWil_1; RETWil_2; SNNP_1; SUN1_1; SUN3_1; SUN3_2; SUN4_1; V24_1; V25_1; V28_1; V28_2; V36_1; V37_1; WA026_1; WA026_2; WA117_1; WA117_2; WA606_1; WA606_2; WA609_1; WA609_2; WA612_1; WA614_1; WA619_1; WA619_2; WA621_1; WA621_2; WA622_1; WA622_2; WA623_1; WA623_2; WA700_1; WA700_2; WA705_1; WA705_2; WA707_1; WA707_2; WA708_2; WA720_1; WA720_2
**8**	***giraffa*_BNP02_1**	**1**	BNP02_1
**9**	***giraffa*_BNP02_2**	**45**	BNP02_2; BNP03_2; BNP05_1; BNP05_2; BNP06_2; CKGR03_2; CNP01_1; CNP01_2; CNP02_1; CNP02_2; ENP04_2; ENP08_2; ENP11_2; ENP14_2; ENP20_2; MGR02_1; MGR02_2; MGR03_2; MGR04_1; MGR04_2; MGR05_1; MGR05_2; SUN1_2; SUN2_1; SUN2_2; SUN4_2; V23_1; V23_2; V24_2; V26_1; V26_2; V27_1; V27_2; V29_1; V29_2; V30_1; V30_2; V31_1; V31_2; V36_2; V37_2; V38_1; V38_2; V39_1; V39_2

Table T. Overview of the respective alleles comprised by the haplotypes shown in the network of *USP33*.

n	Representative individual	∑	Included Alleles per Haplotypes
1	***camelopardalis*_BaNP4_1**	2	BaNP4_1; BaNP4_2
2	***camelopardalis*_ETH1_1**	37	ETH1_1; ETH2_1; ETH3_1; GNP02_1; GNP04_1; MF02_1; MF02_2; MF03_1; MF04_1; MF04_2; MF05_1; MF06_1; MF11_1; MF13_1; MF14_1; MF16_1; MF17_1; MF24_1; MF24_2; RET4_1; RET5_1; RET5_2; RET6_1; RETWil2_1; ISC04_1; LWC01_1; SNR1_1; SNR1_2; SNR2_1; SNR2_2; WA036_1; WA606_1; WA606_2; WA612_1; WA621_1; ZNP01_1; ZNP01_2
3	***camelopardalis*_ETH1_2**	38	ETH1_2; ETH2_2; ETH3_2; GNP01_1; GNP01_2; GNP02_2; GNP03_1; GNP03_2; GNP04_2; GNP05_1; GNP05_2; MF01_1; MF06_2; MF11_2; MF13_2; MF16_2; RET3_1; RET3_2; RETRot1_1; RETRot1_2; RETRot2_1; RETWil_1; RETWil2_2; ISC03_1; ISC08_1; ISC08_2; WA026_2; WA036_2; WA117_2; WA612_2; WA614_1; WA622_1; WA622_2; WA623_1; WA623_2; WA705_2; WA707_1; WA707_2
4	***rothschildi*_MF01_2**	11	MF01_2; MF03_2; MF05_2; MF07_1; MF07_2; MF09_1; MF09_2; MF14_2; MF15_1; MF15_2; MF17_2
5	***peralta*_WA026_1**	1	WA026_1
6	***peralta*_WA609_1**	5	WA609_1; WA609_2; WA619_2; WA621_2; WA700_2
7	***peralta*_WA614_2**	3	WA614_2; WA708_2; WA720_2
8	***peralta*_WA117_1**	6	WA117_1; WA619_1; WA700_1; WA705_1; WA708_1; WA720_1
9	***reticulata*_RET1_1**	6	RET1_1; RET1_2; RETRot2_2; RETRot3_2; ISC04_2; LWC01_2
10	***reticulata*_RET4_2**	4	RET4_2; RET6_2; RETWil_2; ISC03_2
11	***reticulata*_RETRot3_1**	1	RETRot3_1
12	***thornicrofti*_LVNP18_1**	36	LVNP18_1; LVNP18_2; LVNP19_1; LVNP19_2; LVNP20_1; LVNP20_2; LVNP21_1; LVNP21_2; LVNP22_1; LVNP22_2; LVNP23a_1; LVNP23a_2; LVNP31_1; LVNP31_2; LVNP32_1; LVNP32_2; LVNP33_1; LVNP33_2; LVNP34_1; LVNP34_2; LVNP35_1; LVNP35_2; LVNP36_1; LVNP36_2; SGR01_1; SGR01_2; SGR05_1; SGR05_2; SGR06_1; SGR07_1; SGR07_2; SGR12_1; SGR12_2; SGR13_1; SGR13_2; SGR14_1
13	***tippelskirchi*_SGR06_2**	1	SGR06_2
14	***tippelskirchi*_SGR14_2**	1	SGR14_2
15	***giraffa*_MGR01_2**	1	MGR01_2
16	***angolensis*_CKGR01_1**	18	CKGR01_1; CKGR01_2; CKGR02_1; CKGR02_2; CKGR05_2; CNP03_2; ENP07_2; ENP08_1; ENP08_2; ENP09_2; ENP14_2; ENP15_1; ENP15_2; ENP16_1; ENP16_2; ENP17_2; ENP19_2; V29_2
17	***giraffa*_BNP01_1**	103	BNP01_1; BNP01_2; BNP02_1; BNP02_2; BNP03_1; BNP03_2; BNP04_1; BNP04_2; BNP05_1; BNP05_2; BNP06_1; BNP06_2; BNP09_1; BNP09_2; CKGR03_1; CKGR03_2; CKGR05_1; CNP01_1; CNP01_2; CNP02_1; CNP02_2; CNP03_1; ENP04_1; ENP04_2; ENP07_1; ENP09_1; ENP11_1; ENP11_2; ENP12_1; ENP12_2; ENP14_1; ENP17_1; ENP18_1; ENP18_2; ENP19_1; ENP20_1; ENP20_2; ENP21_1; ENP21_2; KKR01_1; KKR01_2; KKR02_1; KKR02_2; KKR03_1; KKR03_2; KKR04_1; KKR04_2; KKR05_1; KKR05_2; KKR07_1; KKR07_2; KKR08_1; KKR08_2; MGR01_1; MGR02_1; MGR02_2; MGR03_1; MGR03_2; MGR04_1; MGR04_2; MGR05_1; MGR05_2; MTNP01_1; MTNP01_2; MTNP02_1; MTNP02_2; MTNP03_1; MTNP03_2; SNNP_1; SNNP_2; SUN1_1; SUN1_2; SUN2_1; SUN2_2; SUN3_1; SUN3_2; SUN4_1; SUN4_2; V23_1; V23_2; V24_1; V24_2; V25_1; V25_2; V26_1; V26_2; V27_1; V27_2; V28_1; V28_2; V29_1; V30_1; V30_2; V31_1; V31_2; V36_1; V36_2; V37_1; V37_2; V38_1; V38_2; V39_1; V39_2

Table U. Overview of the respective alleles comprised by the haplotypes shown in the network of *USP54*.

n	Representative individual	∑	Included Alleles per Haplotypes
1	*camelopardalis*_BaNP4_1	35	BaNP4_1; ETH1_1; ETH2_1; GNP05_1; MF01_1; MF02_1; MF03_1; MF03_2; MF05_1; MF05_2; MF06_1; MF07_1; MF09_1; MF09_2; MF11_1; MF13_1; MF14_1; MF15_1; MF15_2; MF16_1; MF16_2; MF17_1; MF17_2; MF24_1; MF24_2; RETRot2_1; SNR1_1; SNR2_1; WA026_1; WA026_2; WA036_1; WA606_1; WA606_2; WA700_1; WA720_1
2	*camelopardalis*_BaNP4_2	32	BaNP4_2; ETH1_2; ETH2_2; GNP01_1; GNP01_2; GNP02_1; GNP02_2; GNP03_1; GNP03_2; GNP04_1; GNP04_2; GNP05_2; MF01_2; MF04_1; MF04_2; MF06_2; MF07_2; MF11_2; MF13_2; MF14_2; RET3_1; SNR1_2; SNR2_2; WA612_1; WA614_1; WA621_1; WA622_1; WA623_1; WA705_1; WA708_1; WA708_2; ZNP01_2
3	*camelopardalis*_ETH3_1	1	ETH3_1
4	*camelopardalis*_ETH3_2	1	ETH3_2
5	*antiquorum*_ZNP01_1	1	ZNP01_1
6	*rothschildi*_MF02_2	1	MF02_2
7	*reticulata*_RET1_1	20	RET1_1; RET1_2; RET4_1; RET4_2; RET5_1; RET6_1; RET6_2; RETRot1_1; RETRot1_2; RETRot3_1; RETRot3_2; RETWil_1; RETWil_2; RETWil2_1; RETWil2_2; ISC03_1; ISC04_1; ISC04_2; ISC08_1; ISC08_2
8	*reticulata*_RET3_2	2	RET3_2; RETRot2_2
9	*reticulata*_RET5_2	2	RET5_2; ISC03_2
10	*reticulata*_LWC01_1	18	LWC01_1; WA036_2; WA117_1; WA117_2; WA609_1; WA609_2; WA612_2; WA614_2; WA619_1; WA619_2; WA621_2; WA622_2; WA623_2; WA700_2; WA705_2; WA707_1; WA707_2; WA720_2
11	*reticulata*_LWC01_2	1	LWC01_2
12	*thornicrofti*_LVNP18_1	9	LVNP18_1; LVNP18_2; LVNP20_1; LVNP20_2; LVNP21_2; LVNP23a_2; LVNP31_2; LVNP32_2; LVNP36_2
13	*thornicrofti*_LVNP19_1	21	LVNP19_1; LVNP19_2; LVNP21_1; LVNP22_1; LVNP22_2; LVNP23a_1; LVNP32_1; LVNP33_1; LVNP33_2; LVNP35_1; LVNP35_2; LVNP36_1; SGR01_1; SGR01_2; SGR06_1; SGR06_2; SGR07_1; SGR13_1; SGR13_2; SGR14_1; SGR14_2
14	*thornicrofti*_LVNP34_2	2	LVNP34_2; SGR05_1
15	*giraffa*_MTNP02_2	1	MTNP02_2
16	*tippelskirchi*_SGR05_2	1	SGR05_2
17	*tippelskirchi*_SGR07_2	3	SGR07_2; SGR12_1; SGR12_2;
18	*angolensis*_CKGR05_1	67	CKGR05_1; CNP01_1; CNP02_1; CNP03_1; ENP04_1; ENP04_2; ENP07_1; ENP07_2; ENP08_1; ENP08_2; ENP09_1; ENP11_1; ENP11_2; ENP15_1; ENP16_1; ENP16_2; ENP17_1; ENP17_2; ENP18_1; ENP18_2; ENP19_1; ENP19_2; ENP20_1; ENP21_1; KKR01_1; KKR01_2; KKR03_1; KKR04_1; KKR04_2; KKR05_1; KKR07_1; LVNP31_1; LVNP34_1; MGR01_1; MGR01_2; MGR02_1; MGR02_2; MGR03_1; MGR03_2; MGR04_1; MGR05_1; MGR05_2; MTNP01_1; MTNP01_2; MTNP03_1; MTNP03_2; SUN1_1; SUN1_2; SUN2_1; SUN2_2; SUN3_1; SUN3_2; SUN4_1; SUN4_2; V23_1; V26_1; V27_1; V28_1; V29_1; V29_2; V30_1; V30_2; V31_1; V37_1; V38_1; V38_2; V39_1
19	*giraffa*_BNP01_1	56	BNP01_1; BNP01_2; BNP02_1; BNP02_2; BNP03_1; BNP03_2; BNP04_1; BNP04_2; BNP05_1; BNP05_2; BNP06_1; BNP06_2; BNP09_1; BNP09_2;CKGR01_1; CKGR01_2; CKGR02_1; CKGR02_2; CKGR03_1; CKGR03_2; CKGR05_2; CNP01_2; CNP02_2; CNP03_2; ENP09_2; ENP12_1; ENP12_2; ENP14_1; ENP14_2; ENP15_2; ENP20_2; ENP21_2; KKR02_1; KKR02_2; KKR03_2; KKR05_2; KKR07_2; KKR08_1; KKR08_2; MGR04_2; MTNP02_1; SNNP_1; SNNP_2; V23_2; V24_1; V24_2; V25_1; V25_2; V26_2; V27_2; V28_2; V31_2; V36_1; V36_2; V37_2; V39_2

Tables V-Ap. List of alleles comprised by the haplotypes shown in the haplowebs constructed for each of the 21 nuclear introns (Fig 4).

Table V. Overview of the alleles comprised by the haplotypes shown in the haploweb of *ACP5*. Groups of individuals sharing an exclusive allele pool [field for recombination: FFR] are highlighted by a grey box.

n	Representative allele	∑	Allele sharing information
1	*giraffa*_BNP01_1	58	BNP01_1; BNP02_1; BNP03_1; BNP04_1; BNP05_1; BNP06_1; BNP09_1; CKGR01_1; CKGR02_1; CKGR03_1; CKGR05_1; CNP02_1; CNP03_1; ENP04_1; ENP07_1; ENP08_1; ENP09_1; ENP11_1; ENP12_1; ENP14_1; ENP15_1; ENP16_1; ENP17_1; ENP18_1; ENP19_1; ENP20_1; ENP21_1; KKR01_1; KKR02_1; KKR03_1; KKR04_1; KKR05_1; KKR07_1; KKR08_1; MGR02_1; MGR03_1; MGR04_1; MGR05_1; MTNP01_1; MTNP02_1; MTNP03_1; SNNP_1; SUN1_1; SUN2_1; SUN3_1; SUN4_1; V23_1; V24_1; V25_1; V26_1; V27_1; V28_1; V29_1; V30_1; V31_1; V36_1; V38_1; V39_1
2	*giraffa*_CNP03_2	7	CNP03_2; MGR02_2; V27_2; V38_2; V39_2; MGR01_1; V37_1
3	*angolensis*_ENP07_2	4	ENP07_2; ENP11_2; ENP14_2; ENP17_2
4	*giraffa*_MGR05_2	4	MGR05_2; MTNP01_2; MTNP03_2; SNNP_2
5	*giraffa*_V23_2	3	V23_2; V36_2; CNP01_1
6	*thornicrofti*_LVNP18_1	3	LVNP18_1; LVNP19_1; LVNP21_1
7	*thornicrofti*_LVNP33_1	1	LVNP33_1
8	*thornicrofti*_LVNP22_1	5	LVNP22_1; LVNP23a_1; LVNP31_1; LVNP32_1; LVNP34_1
9	*thornicrofti*_LVNP20_1	11	LVNP20_1; LVNP22_2; LVNP35_1; LVNP36_2; SGR01_1; SGR05_1; SGR06_1; SGR07_1; SGR12_1; SGR13_1; SGR14_1
10	*thornicrofti*_LVNP36_1	3	LVNP36_1; MF11_2; RETWil2_2
11	*peralta*_WA700_1	1	WA700_1
12	*antiquorum*_GNP04_1	38	GNP04_1; SNR1_1; WA026_1; WA117_1; WA614_1; WA619_1; WA622_1; ZNP01_1; ETH1_1; ETH2_1; GNP01_1; GNP02_1; GNP03_1; GNP05_1; MF01_1; MF02_1; MF03_2; MF04_1; MF05_2; MF06_1; MF07_1; MF09_1; MF13_1; MF14_2; MF15_1; MF16_1; MF17_1; MF24_1; RETRot3_1; SNR2_1; WA036_1; WA606_1, WA621_1; WA623_1; WA705_2; WA707_1; WA708_2; WA720_1
13	*rothschildi*_MF14_1	2	MF14_1; WA705_1
14	*camelopardalis*_BaNP4_1	12	BaNP4_1; ETH3_1; GNP04_2; SNR1_2; WA026_2; WA117_2; WA609_1; WA612_1; WA614_2; WA619_2; WA622_2; ZNP01_2
15	*rothschildi*_MF03_1	2	MF03_1; MF05_1
16	*reticulata*_RETRot3_2	11	RETRot3_2; RET1_1; RET3_1; RET4_1; RET5_1; RET6_1; RETRot1_1; RETRot2_1; ISC03_1; ISC04_1; LWC01_1
17	*reticulata*_RETWil_1	1	RETWil_1

Table W. Overview of the alleles comprised by the haplotypes shown in the haploweb of *C1orf74*. Groups of individuals sharing an exclusive allele pool [field for recombination: FFR] are highlighted by a grey box.

n	Representative allele	∑	Allele sharing information
1	*giraffa*_BNP01_1	61	BNP01_1; BNP02_1; BNP03_1; BNP04_1; BNP05_1; BNP06_1; BNP09_1; CKGR01_1; CKGR02_1; CKGR03_1; CKGR05_1; CNP01_1; CNP02_1; CNP03_1; ENP04_1; ENP07_1; ENP08_1; ENP09_1; ENP11_1; ENP12_1; ENP14_1; ENP15_1; ENP16_1; ENP17_1; ENP18_1; ENP19_1; ENP20_1; ENP21_1; KKR01_1; KKR02_1; KKR03_1; KKR04_1; KKR05_1; KKR07_1; KKR08_1; MGR01_1; MGR02_1; MGR03_1; MGR04_1; MGR05_1; MTNP01_1; MTNP02_1; MTNP03_1; SNNP_1; SUN1_1; SUN2_1; SUN3_1; SUN4_1; V23_1; V24_1; V25_1; V26_1; V27_1; V28_1; V29_1; V30_1; V31_1; V36_1; V37_1; V38_1; V39_1
2	*thornicrofti*_LVNP18_1	19	LVNP18_1; LVNP19_1; LVNP20_1; LVNP21_1; LVNP22_1; LVNP23a_1; LVNP31_1; LVNP32_1; LVNP33_1; LVNP34_1; LVNP35_1; LVNP36_1; SGR01_1; SGR05_1; SGR06_1; SGR07_1; SGR12_1; SGR13_1; SGR14_2
3	*camelopardalis*_BaNP4_1	2	BaNP4_1; MF07_1
4	*camelopardalis*_ETH1_1	45	ETH1_1; ETH2_1; ETH3_1; GNP01_1; GNP02_1; GNP03_1; GNP04_1; GNP05_1; MF02_1; MF04_1; MF09_1; MF11_1; MF14_1; MF24_1; RET1_1; RET3_1; RET4_1; RET5_1; RETRot1_1; RETRot2_1; RETRot3_1; RETWil_1; ISC03_1; ISC04_1; ISC08_1; LWC01_1; SNR1_1; SNR2_1; WA026_1; WA036_1; WA117_1; WA606_1; WA609_1; WA612_1; WA614_1; WA619_1; WA621_1; WA622_1; WA623_1; WA700_1; WA705_1; WA707_1; WA708_1; WA720_1; ZNP01_1
5	*reticulata*_RETRot1_2	5	RETRot1_2; RETWil_2; ISC04_2; ISC08_2; RETWil2_1
6	*reticulata*_RET1_1	1	RET1_2; RET4_2; RET5_2; RET6_1
7	*reticulata*_RET3_2	6	RET3_2; RETRot3_2; LWC01_2; MF05_1; MF06_1; MF15_1
8	*rothschildi*_MF02_2	8	MF02_2; MF01_1; MF03_1; MF05_2; MF13_1; MF16_1; MF17_2; RETWil2_2

Table X. Overview of the alleles comprised by the haplotypes shown in the haploweb of *CCT2*. Groups of individuals sharing an exclusive allele pool [field for recombination: FFR] are highlighted by a grey box.

n	Representative allele	∑	Allele sharing information
1	*camelopardalis*_BaNP4_1	96	BaNP4_1; BNP02_1; BNP03_1; BNP04_1; BNP05_1; BNP06_1; BNP09_1; CKGR01_1; CKGR02_1; CKGR03_1; CKGR05_1; CNP01_1; CNP02_1; CNP03_1; ENP04_1; ENP07_1; ENP09_1; ENP11_1; ENP12_1; ENP14_1; ENP15_1; ENP16_1; ENP17_1; ENP18_1; ENP19_1; ENP20_1; ENP21_1; GNP01_1; GNP05_1; KKR01_1; KKR02_1; KKR03_1; KKR04_1; KKR05_1; KKR07_1; KKR08_1; LVNP18_1; LVNP19_1; LVNP20_1; LVNP21_1; LVNP22_1; LVNP23a_1; LVNP31_1; LVNP32_1; LVNP33_1; LVNP34_1; LVNP35_1; LVNP36_1; MF01_1; MF03_1; MF06_1; MGR01_1; MGR02_1; MGR03_1; MGR04_1; MGR05_1; MTNP01_1; MTNP02_1; MTNP03_1; RET1_1; RET5_1; RET6_1; RETWil_1; SGR01_1; SGR05_1; SGR06_1; SGR07_1; SGR12_1; SGR13_1; SGR14_1; SNNP_1; SNR1_1; SUN1_1; SUN2_1; SUN3_1; SUN4_1; V23_1; V24_1; V25_1; V26_1; V27_1; V28_1; V29_1; V30_1; V31_1; V36_1; V37_1; V38_1; V39_1; WA026_1; WA036_1; WA117_1; WA606_1; WA707_1; WA708_1; ZNP01_1

2	***camelopardalis*_BaNP4_2**	50	BaNP4_2; GNP01_2; MF01_2; MF03_2; MF06_2; RET1_2; RET5_2; RET6_2; RETWil_2; WA036_2; WA606_2; WA707_2; ZNP01_2; BNP01_1; ENP08_1; ETH1_1; ETH2_1; GNP02_1; GNP03_1; GNP04_1; MF02_1; MF04_1; MF05_1; MF07_1; MF09_1; MF11_1; MF13_1; MF14_1; MF15_1; MF16_1; MF17_1; MF24_1; RET3_1; RET4_1; RETRot3_1; ISC03_1; ISC04_1; ISC08_1; LWC01_1; SNR2_1; WA609_1; WA612_1; WA614_1; WA619_1; WA621_1; WA622_1; WA623_1; WA700_1; WA705_1; WA720_1
3	***antiquorum*_SNR1_2**	2	SNR1_2; ETH3_1
4	***reticulata*_RETWil2_2**	3	RETWil2_2; RETRot1_1; RETRot2_1
5	***reticulata*_RET4_2**	4	RET4_2; RETRot3_2; ISC04_2; RETWil2_1

Table Y. Overview of the alleles comprised by the haplotypes shown in the haploweb of *COL5A2*. Groups of individuals sharing an exclusive allele pool [field for recombination: FFR] are highlighted by a grey box.

n	Representative allele	∑	Allele sharing information
1	***camelopardalis*_BaNP4_1**	12	BaNP4_1; MF01_1; MF03_1; MF04_1; MF06_1; MF09_1; MF13_1; MF14_1; MF15_1; MF16_1; MF17_1; MF24_1
2	***rothschildi*_MF01_2**	31	MF01_2; MF03_2; MF06_2; MF17_2; MF24_2; RETRot1_1; ETH1_1; ETH2_1; ETH3_1; GNP01_1; GNP02_1; GNP03_1; GNP04_1; GNP05_1; MF05_1; MF07_1; MF11_1; RETRot2_1; SNR1_1; SNR2_1; WA606_1; WA609_1; WA612_1; WA614_1; WA619_1; WA621_1; WA700_1; WA705_1; WA708_1; WA720_1; ZNP01_1
3	***rothschildi*_MF09_2**	6	MF09_2; WA609_2; MF02_2; RET3_2; RETWil2_1; LWC01_2
4	***rothschildi*_MF13_2**	2	MF13_2; MF02_1
5	***peralta*_WA612_2**	8	WA612_2; WA619_2; WA700_2; WA720_2; WA026_1; WA036_1; WA622_1; WA623_1
6	***reticulata*_RET6_1**	7	RET6_1; ISC04_1; RET3_1; LWC01_1; RET4_1; ISC03_1; RET5_1
7	***reticulata*_RET1_2**	3	RET1_2; RET4_2; ISC03_2
8	***reticulata*_RETRot3_2**	2	RETRot3_2; RETWil_1
9	***giraffa*_BNP06_2**	6	BNP06_2; ENP17_2; MGR01_2; MGR04_2; V23_2; V29_2
10	***giraffa*_BNP01_1**	66	BNP01_1; BNP02_1; BNP03_1; BNP04_1; BNP05_1; BNP06_1; BNP09_1; CKGR01_1; CKGR02_1; CKGR03_1; CKGR05_1; CNP01_1; CNP02_1; CNP03_1; ENP04_1; ENP07_1; ENP08_1; ENP09_1; ENP11_1; ENP12_1; ENP14_1; ENP15_1; ENP16_1; ENP17_1; ENP18_1; ENP19_1; ENP20_1; ENP21_1; KKR01_1; KKR02_1; KKR03_1; KKR04_1; KKR05_1; KKR07_1; KKR08_1; MGR01_1; MGR02_1; MGR03_1; MGR04_1; MGR05_1; MTNP01_1; MTNP02_1; MTNP03_1; RET1_1; RET6_2; RETRot1_2; ISC04_2; ISC08_1; SNNP_1; SUN1_1; SUN2_1; SUN3_1; SUN4_1; V23_1; V24_1; V25_1; V26_1; V27_1; V28_1; V29_1; V30_1; V31_1; V36_1; V37_1; V38_1; V39_1
11	***peralta*_WA707_1**	1	WA707_1
12	***thornicrofti*_LVNP18_1**	19	LVNP18_1; LVNP19_1; LVNP20_1; LVNP21_1; LVNP22_1; LVNP23a_1; LVNP31_1; LVNP32_1; LVNP33_1; LVNP34_1; LVNP35_1; LVNP36_1; SGR01_1; SGR05_1; SGR06_1; SGR07_2; SGR12_1; SGR13_1; SGR14_1
13	***thornicrofti*_LVNP33_2**	2	LVNP33_2; LVNP34_2

Table Z. Overview of the alleles comprised by the haplotypes shown in the haploweb of *CTAGE5*. Groups of individuals sharing an exclusive allele pool [field for recombination: FFR] are highlighted by a grey box.

n	Representative allele	∑	Allele sharing information
1	*camelopardalis*_BaNP4_1	28	BaNP4_1; ETH3_1; MF01_1; MF02_1; MF03_1; MF04_1; MF05_1; MF06_1; MF07_1; MF09_1; MF11_1; MF13_1; MF14_1; MF15_1; MF24_1; RET1_1; RET4_1; RET5_1; RET6_1; RETRot1_1; RETRot2_1; RETRot3_1; RETWil_1; ISC03_1; ISC08_1; WA036_1; WA619_1; WA623_1
2	*camelopardalis*_ETH3_2	44	ETH3_2; MF01_2; MF03_2; MF04_2; MF05_2; MF06_2; MF07_2; MF09_2; MF11_2; MF13_2; MF14_2; MF15_2; RETRot2_2; RETRot3_2; RETWil_2; ISC03_2; ISC08_2; WA036_2; ETH1_1; ETH2_2; GNP01_1; GNP02_1; GNP03_1; GNP04_1; GNP05_1; MF16_1; MF17_1; RET3_1; SNR1_1; SNR2_1; WA026_1; WA117_1; WA606_1; WA609_1; WA612_1; WA614_1; WA621_1; WA622_1; WA700_1; WA705_2; WA707_1; WA708_2; WA720_1; ZNP01_1
3	*peralta*_WA619_2	4	WA619_2; WA623_2; WA705_1; WA708_1
4	*peralta*_WA026_2	2	WA026_2; WA720_2
5	*reticulata*_LWC01_1	1	LWC01_1
6	*reticulata*_RETWil2_2	2	RETWil2_2; ISC04_1
7	*thornicrofti*_LVNP18_1	16	LVNP18_1; LVNP19_1; LVNP20_1; LVNP21_1; LVNP22_1; LVNP23a_1; LVNP31_1; LVNP32_1; LVNP33_1; LVNP34_1; LVNP35_1; LVNP36_1; SGR01_2; SGR06_1; SGR12_2; SGR14_1
8	*giraffa*_BNP01_1	53	BNP01_1; BNP02_1; BNP03_1; BNP04_1; BNP05_1; BNP06_1; BNP09_1; CKGR01_1; CKGR02_1; CNP01_1; CNP03_1; ENP04_1; ENP07_1; ENP08_1; ENP09_1; ENP11_1; ENP12_1; ENP14_1; ENP15_1; ENP16_1; ENP17_1; ENP18_1; ENP19_1; ENP20_1; ENP21_1; KKR01_1; KKR02_1; KKR03_1; KKR04_1; KKR05_1; MGR01_1; MGR02_1; MGR03_1; MGR04_1; MGR05_1; MTNP01_1; MTNP02_1; MTNP03_1; SNNP_1; SUN1_1; SUN2_1; SUN3_1; V23_1; V24_1; V25_1; V26_1; V27_1; V28_1; V30_1; V31_1; V36_1; V37_1; V39_1
9	*giraffa*_BNP01_2	15	BNP01_2; BNP09_2; ENP04_2; ENP17_2; ENP18_2; KKR01_2; KKR03_2; SUN2_2; SUN3_2; V24_2; CKGR03_1; CKGR05_1; KKR08_1; SUN4_1; V29_1
10	*giraffa*_BNP04_2	4	BNP04_2; V29_2; KKR07_1; V38_2
11	*giraffa*_MTNP01_2	3	MTNP01_2; MTNP03_2; V38_1
12	*giraffa*_CNP02_1	1	CNP02_1

Table Aa. Overview of the alleles comprised by the haplotypes shown in the haploweb of *CWF19L1*. Groups of individuals sharing an exclusive allele pool [field for recombination: FFR] are highlighted by a grey box.

n	Representative allele	∑	Allele sharing information
1	***camelopardalis*_BaNP4_1**	48	BaNP4_1; ETH1_1; ETH2_1; ETH3_1; GNP01_1; GNP03_1; GNP04_1; GNP05_1, MF02_1; MF03_1; MF04_1; MF06_1; MF09_2; MF15_2; MF17_2; MF24_2; RET1_1; RET3_1; RET4_1; RET5_1; RET6_1; RETRot1_1; RETRot2_1; RETRot3_1; RETWil_1; RETWil2_1; ISC03_1; ISC04_1; ISC08_1; LWC01_1; SNR1_1; SNR2_1; WA026_1; WA036_1; WA117_1; WA606_1; WA609_1; WA612_1; WA614_1; WA619_1; WA621_1; WA622_1; WA623_1; WA700_1; WA705_1; WA707_1; WA720_1; ZNP01_1
2	***camelopardalis*_BaNP4_2**	22	BaNP4_2; ETH1_2; GNP01_2; GNP03_2; GNP05_2; MF02_2; MF03_2; MF04_2; MF06_2; MF09_1; MF15_1; MF17_1; MF24_1; RETWil_2; ISC03_2; SNR2_2; GNP02_1; MF01_1; MF05_1; MF07_1; MF11_1; MF16_1
3	***rothschildi*_MF13_1**	2	MF13_1; MF14_1
4	***peralta*_WA708_1**	1	WA708_1
5	***giraffa*_BNP01_1**	74	BNP01_1; BNP02_1; BNP04_1; BNP05_1; BNP09_1; CKGR02_1; CKGR03_1; CKGR05_1; CNP01_1; CNP02_1; CNP03_1; ENP04_1; ENP07_1; ENP08_1; ENP09_1; ENP11_1; ENP12_1; ENP14_1; ENP15_1; ENP16_1; ENP17_1; ENP20_1; ENP21_1; KKR01_1; KKR03_1; KKR04_1; KKR05_1; KKR07_1; KKR08_1; LVNP18_1; LVNP19_1; LVNP20_1; LVNP21_1; LVNP22_1; LVNP23a_1; LVNP31_1; LVNP32_1; LVNP33_1; LVNP34_1; LVNP35_1; LVNP36_1; MGR01_1; MGR02_1; MGR03_1; MGR04_1; MGR05_1; MTNP01_1; MTNP02_1; MTNP03_1; SGR01_1; SGR05_1; SGR06_1; SGR07_1; SGR12_1; SGR13_1; SGR14_1; SNNP_1; SUN1_1; SUN2_1; SUN3_1; SUN4_1; V23_1; V24_1; V25_1; V26_1; V27_1; V28_1; V29_1; V30_1; V31_1; V36_1; V37_1; V38_1; V39_1
6	***giraffa*_BNP02_2**	21	BNP02_2; CNP02_2; ENP04_2; ENP08_2; ENP15_2; ENP16_2; ENP20_2; ENP21_2; KKR04_2; MGR02_2; SGR07_2; SGR12_2; V28_2; V36_2; V38_2; BNP03_1; BNP06_1; CKGR01_1; ENP18_1; ENP19_1; KKR02_1
7	***giraffa*_V27_1**	2	V27_2; V39_2

Table Ab. Overview of the alleles comprised by the haplotypes shown in the haploweb of *DDX1*. Groups of individuals sharing an exclusive allele pool [field for recombination: FFR] are highlighted by a grey box.

n	Representative allele	∑	Allele sharing information
1	***camelopardalis*_BaNP4_2**	53	BaNP4_2; ETH1_1; ETH2_1; ETH3_1; GNP01_1; GNP02_1; GNP03_1; GNP04_1; GNP05_1; MF01_1; MF02_1; MF03_1; MF04_1; MF05_1; MF06_1; MF07_1; MF09_1; MF11_1; MF13_1; MF14_1; MF15_1; MF16_1; MF17_1; MF24_1; RET1_2; RET3_1; RET6_2; RETRot2_1; RETWil_1; RETWil2_1; ISC03_1; ISC04_1; ISC08_1; LWC01_1; SNR1_1; SNR2_1; WA026_1; WA036_1; WA117_1; WA606_1; WA609_1; WA612_1; WA614_1; WA619_1; WA621_1; WA622_1; WA623_1; WA700_1; WA705_1; WA707_1; WA708_1; WA720_1; ZNP01_1
2	***reticulata*_RETWil2_2**	2	RETWil2_2; ISC04_2
3	***reticulata*_RET1_1**	6	RET1_1; RET6_1; RET4_1; RET5_1; RETRot1_1; RETRot3_1
4	***giraffa*_BNP01_1**	50	BNP01_1; BNP02_1; BNP03_1; BNP04_1; BNP05_1; BNP06_1; BNP09_1; CKGR01_1; CKGR02_1; CKGR03_1; CKGR05_1; CNP01_1; CNP02_1;

			CNP03_1; ENP04_1; ENP07_1; ENP08_1; ENP09_1; ENP11_1; ENP12_1; ENP14_1; ENP15_1; ENP16_1; ENP17_1; ENP18_1; ENP19_1; ENP20_1; ENP21_1; MGR01_1; MGR02_1; MGR03_1; MGR04_1; MGR05_1; MTNP01_1; MTNP02_1; MTNP03_1; SNNP_1; V23_1; V24_1; V25_1; V26_1; V27_1; V28_1; V29_1; V30_1; V31_1; V36_1; V37_1; V38_1; V39_1
**5**	***giraffa*_MGR02_2**	**3**	MGR02_2; V27_2; V39_2
**6**	***giraffa*_KKR01_1**	**26**	KKR01_1; KKR02_1; KKR03_1; KKR04_1; KKR05_1; KKR07_1; KKR08_1; LVNP18_1; LVNP19_1; LVNP20_1; LVNP21_1; LVNP22_1; LVNP23a_1; LVNP31_1; LVNP32_1; LVNP34_1; LVNP35_1; SGR05_1; SGR06_1; SGR07_1; SGR12_1; SGR13_1; SUN1_1; SUN2_1; SUN3_1; SUN4_1
**7**	***thornicrofti*_LVNP32_2**	**5**	LVNP32_2; LVNP33_1; LVNP36_2; SGR01_2; SGR14_2
**8**	***thornicrofti*_LVNP36_1**	**3**	LVNP36_1; SGR01_1; SGR14_1

Table Ac. Overview of the alleles comprised by the haplotypes shown in the haploweb of *DHX36*. Groups of individuals sharing an exclusive allele pool [field for recombination: FFR] are highlighted by a grey box.

n	Representative allele	∑	Allele sharing information
**1**	***camelopardalis*_BaNP4_1**	**42**	BaNP4_1; ETH3_1; GNP03_1; LVNP18_1; LVNP19_1; LVNP20_1; LVNP21_1; LVNP22_1; LVNP23a_1; LVNP31_1; LVNP32_1; LVNP33_1; LVNP34_1; LVNP35_1; LVNP36_1; RET1_1; RET5_1; RET6_1; RETRot3_1; RETWil_1; RETWil2_1; ISC04_1; SGR01_1; SGR05_1; SGR06_1; SGR07_1; SGR12_1; SGR13_1; SGR14_1; SNR1_1; WA026_1; WA036_1; WA606_1; WA609_1; WA612_1; WA614_1; WA622_1; WA623_1; WA700_1; WA705_1; WA707_1; WA708_1
**2**	***camelopardalis*_ETH3_2**	**24**	ETH3_2; ETH1_1; ETH2_2; GNP02_2; GNP04_2; MF01_1; MF02_1; MF03_2; MF04_1; MF05_1; MF06_1; MF07_1; MF09_1; MF11_1; MF13_2; MF14_1; MF15_1; MF16_1; MF17_1; MF24_2; LWC01_1; SNR2_1; WA117_1; WA720_2
**3**	***reticulata*_LWC01_2**	**2**	LWC01_2; RETRot2_2
**4**	***reticulata*_RETWil_2**	**17**	RETWil_2; WA612_2; WA622_2; ETH2_1; GNP02_1; GNP04_1; MF03_1; MF13_1; MF24_1; WA720_1; GNP01_1; GNP05_1; RET3_1; RET4_1; RETRot1_1; WA619_1; ZNP01_1
**5**	***peralta*_WA036_2**	**4**	WA036_2; WA614_2; WA707_2; WA621_1
**6**	***reticulata*_RET5_2**	**6**	RET5_2; RET6_2; RET4_2; RETRot1_2; RETRot2_1; ISC03_1
**7**	***antiquorum*_GNP03_2**	**2**	GNP03_2; SNR1_2
**8**	***giraffa*_BNP01_1**	**61**	BNP01_1; BNP02_1; BNP03_1; BNP04_1; BNP05_1; BNP06_1; BNP09_1; CKGR01_1; CKGR02_1; CKGR03_1; CKGR05_1; CNP01_1; CNP02_1; CNP03_1; ENP04_1; ENP07_1; ENP08_1; ENP09_1; ENP11_1; ENP12_1; ENP14_1; ENP15_1; ENP16_1; ENP17_1; ENP18_1; ENP19_1; ENP20_1; ENP21_1; KKR01_1; KKR02_1; KKR03_1; KKR04_1; KKR05_1; KKR07_1; KKR08_1; MGR01_1; MGR02_1; MGR03_1; MGR04_1; MGR05_1; MTNP01_1; MTNP02_1; MTNP03_1; SNNP_1; SUN1_1; SUN2_1; SUN3_1; SUN4_1; V23_1; V24_1; V25_1; V26_1; V27_1; V28_1; V29_1; V30_1; V31_1; V36_1; V37_1; V38_1; V39_1

Table Ad. Overview of the alleles comprised by the haplotypes shown in the haploweb of *IGF2B1*. Groups of individuals sharing an exclusive allele pool [field for recombination: FFR] are highlighted by a grey box.

n	Representative allele	∑	Allele sharing information
1	*camelopardalis*_BaNP4_1	50	BaNP4_1; ETH1_1; ETH2_1; ETH3_1; GNP01_1; GNP02_1; GNP04_1; MF01_1; MF02_1; MF03_1; MF04_1; MF05_1; MF06_1; MF07_1; MF09_1; MF11_1; MF13_1; MF16_1; MF17_1; MF24_1; RET1_1; RET3_1; RET4_1; RET5_1; RET6_1; RETRot1_1; RETRot2_1; RETRot3_1; RETWil_1; RETWil2_1; ISC03_1; ISC04_1; ISC08_1; LWC01_1; SGR01_1; SGR05_1; SGR06_1; SGR07_1; SGR12_1; SGR13_1; SGR14_1; SNR1_1; SNR2_1; WA117_1; WA612_1; WA614_1; WA621_1; WA623_1; WA700_1; WA708_1
2	*antiquorum*_GNP01_2	2	GNP01_2; SNR2_2
3	*antiquorum*_GNP04_2	3	GNP04_2; GNP03_1; ZNP01_1
4	*peralta*_WA606_1	3	WA606_1; WA619_1; WA622_1
5	*peralta*_WA614_2	5	WA614_2; WA623_2; WA619_2; WA622_2; WA707_1
6	*peralta*_WA036_2	4	WA036_1; WA609_1; WA705_1; WA720_1
7	*rothschildi*_MF14_1	1	MF14_1
8	*rothschildi*_MF15_1	1	MF15_1
9	*thornicrofti*_LVNP18_2	12	LVNP18_2; LVNP19_1; LVNP20_1; LVNP21_1; LVNP22_1; LVNP23a_1; LVNP31_1; LVNP32_2; LVNP33_1; LVNP34_1; LVNP35_1; LVNP36_1
10	*giraffa*_BNP01_1	59	BNP01_1; BNP02_1; BNP03_1; BNP04_1; BNP05_1; BNP06_1; BNP09_1; CKGR01_1; CKGR02_1; CKGR03_1; CKGR05_1; CNP01_1; CNP02_1; CNP03_1; ENP04_1; ENP07_1; ENP08_1; ENP09_1; ENP11_1; ENP12_1; ENP14_1; ENP15_1; ENP16_1; ENP17_1; ENP18_1; ENP19_1; ENP20_1; ENP21_1; KKR01_1; KKR02_1; KKR03_1; KKR04_1; KKR05_1; KKR07_1; KKR08_1; MGR01_1; MGR02_2; MGR03_1; MGR04_1; MGR05_1; MTNP01_1; MTNP02_1; MTNP03_1; SNNP_1; SUN1_1; SUN2_1; SUN3_1; SUN4_1; V23_1; V24_1; V26_1; V27_1; V28_1; V29_2; V30_1; V31_1; V36_1; V37_1; V39_1
11	*giraffa*_ENP09_2	4	ENP09_2; ENP17_2; ENP20_2; ENP21_2
12	*giraffa*_MGR02_1	3	MGR02_1; V29_1; V25_1

Table Ae. Overview of the alleles comprised by the haplotypes shown in the haploweb of *MACF1*. Groups of individuals sharing an exclusive allele pool [field for recombination: FFR] are highlighted by a grey box.

n	Representative allele	∑	Allele sharing information
1	*camelopardalis*_BaNP4_1	68	BaNP4_1; BNP03_1; BNP06_1; CNP01_1; ETH1_1; ETH2_1; ETH3_1; GNP05_1; LVNP18_1; LVNP19_1; LVNP20_1; LVNP21_1; LVNP22_1; LVNP23a_1; LVNP31_1; LVNP32_1; LVNP33_1; LVNP34_1; LVNP35_1; LVNP36_1; MF01_1; MF02_1; MF03_1; MF05_1; MF06_1; MF07_1; MF09_1; MF11_1; MF13_1; MF14_1; MF16_1; MF17_1; MF24_1; MGR01_1; MGR02_1; MGR03_1; MGR04_1; MGR05_1; RET1_1; RET4_1; RET5_1; RET6_1; RETRot1_1; RETRot2_1; RETRot3_1; RETWil_1; RETWil2_1; ISC04_1; ISC08_1; LWC01_1; SGR05_1; SGR06_1; SGR07_1; SGR12_1; SGR14_1; SNR1_1; SNR2_1; V36_1; WA026_1; WA117_1; WA606_1; WA614_1; WA623_1; WA700_1; WA705_1; WA707_1; WA720_1; ZNP01_1

2	*camelopardalis*_ETH1_2	6	ETH1_2; WA606_2; WA614_2; GNP01_2; WA612_1; WA621_1
3	*camelopardalis*_ETH2_2	11	ETH2_2; ETH3_2; MF01_2; MF02_2; MF06_2; MF07_2; MF11_2; MF14_2; MF17_2; MF04_1; ISC03_1
4	*rothschildi*_MF03_2	18	MF03_2; MF05_2; MF09_2; MF16_2; RET5_2; RETRot1_2; RETWil_2; ISC08_2; ZNP01_2; GNP01_1; GNP02_1; GNP03_1; GNP04_1; MF15_1; RET3_1; WA619_1; WA622_1; WA708_1
5	*peralta*_WA623_2	6	WA623_2; WA700_2; WA621_2; WA708_2; WA036_1; WA609_1
6	*peralta*_WA612_2	2	WA612_2; WA619_2
7	*giraffa*_CNP02_1	2	CNP02_1; V37_1
8	*giraffa*_KKR03_1	16	KKR03_1; KKR05_1; ENP04_1; ENP08_1; ENP17_1; ENP19_1; ENP20_1; MTNP02_1; MTNP01_1; MTNP03_1; SGR01_1; SGR13_1; V25_1; V27_1; V30_1; V39_1
9	*giraffa*_BNP01_1	26	BNP01_1; BNP02_1; BNP04_1; BNP05_1; BNP09_1; CKGR01_1; CKGR02_1; CKGR03_1; CNP03_1; ENP21_1; KKR01_1; KKR03_2; KKR04_1; KKR05_2; KKR07_1; KKR08_1; SNNP_2; SUN1_1; SUN2_1; SUN3_1; SUN4_1; V23_1; V24_1; V26_1; V28_2; V31_1
10	*giraffa*_BNP03_2	24	BNP03_2; BNP06_2; CNP01_2; CKGR02_2; ENP21_2; CKGR05_1; CNP02_2; ENP04_2; ENP07_1; ENP08_2; ENP09_1; ENP11_1; ENP12_1; ENP14_1; ENP15_1; ENP16_1; ENP17_2; ENP18_1; ENP19_2; ENP20_2; MTNP02_2; V29_1; V37_2; V38_1

Table Af. Overview of the alleles comprised by the haplotypes shown in the haploweb of *NOTCH2*. Groups of individuals sharing an exclusive allele pool [field for recombination: FFR] are highlighted by a grey box.

n	Representative allele	∑	Allele sharing information
1	*camelopardalis*_BaNP4_1	62	BaNP4_1; ETH1_1; ETH2_1; ETH3_1; GNP01_1; GNP02_1; GNP03_1; GNP04_1; GNP05_1; LVNP18_1; LVNP19_1; LVNP20_1; LVNP21_1; LVNP22_1; LVNP23a_1; LVNP31_1; LVNP32_1; LVNP33_1; LVNP34_1; LVNP35_1; LVNP36_1; MF01_1; MF02_1; MF03_1; MF04_1; MF05_1; MF09_1; MF13_1; MF15_1; MF16_1; MF17_1; MF24_1; RET3_1; RET4_1; RET5_1; RET6_1; RETRot1_1; RETRot2_1; RETWil_1; ISC03_1; ISC04_1; ISC08_1; LWC01_1; SGR05_1; SGR06_1; SGR07_1; SGR12_1; SGR13_1; SGR14_1; SNR1_1; SNR2_1; WA036_1; WA117_1; WA609_1; WA612_1; WA614_1; WA619_1; WA622_1; WA705_1; WA707_1; WA708_1; WA720_1
2	*rothschildi*_MF04_2	10	MF04_2; MF09_2; MF15_2; MF17_2; SNR1_2; MF06_1; MF07_1; MF11_1; MF14_1; ZNP01_1
3	*peralta*_WA612_2	4	WA612_2; WA026_2; WA623_2; WA700_2
4	*reticulata*_RET3_2	4	RET3_2; LWC01_2; WA623_1; WA700_1
5	*reticulata*_RET4_2	8	RET4_2; RET6_2; RETRot1_2; RETRot2_2; RETWil_2; RET1_2; RETRot3_2; RETWil2_2
6	*reticulata*_ISC03_2	11	ISC03_2; WA609_2; WA622_2; WA707_2; WA720_2; RET1_1; RETRot3_1; RETWil2_1; WA026_1; WA606_2; WA621_1
7	*giraffa*_BNP01_1	62	BNP01_1; BNP02_1; BNP03_1; BNP04_1; BNP05_1; BNP06_1; BNP09_1; CKGR01_1; CKGR02_1; CKGR03_1; CKGR05_1; CNP01_1; CNP02_1; CNP03_1; ENP04_1; ENP07_1; ENP08_1; ENP09_1; ENP11_1; ENP12_1; ENP14_1; ENP15_1; ENP16_1; ENP17_1; ENP18_1; ENP19_1; ENP20_1; ENP21_1; KKR01_1; KKR02_1; KKR03_1; KKR04_1; KKR05_1; KKR07_1; KKR08_1; MGR01_1; MGR02_1; MGR03_1; MGR04_1; MGR05_1; MTNP01_1; MTNP02_1; MTNP03_1; SGR01_2; SNNP_1; SUN1_1; SUN2_1; SUN3_1; SUN4_1; V23_1; V24_1; V25_1; V26_1; V27_1; V28_1; V29_1; V30_1; V31_1; V36_1; V37_1; V38_1; V39_1

Table Ag. Overview of the alleles comprised by the haplotypes shown in the haploweb of *NUP155*. Groups of individuals sharing an exclusive allele pool [field for recombination: FFR] are highlighted by a grey box.

n	Representative allele	∑	Allele sharing information
**1**	***camelopardalis*_BaNP4_1**	**2**	BaNP4_1; SNR1_1
**2**	***camelopardalis*_BaNP4_2**	**2**	BaNP4_2; SNR1_2
**3**	***camelopardalis*_ETH1_1**	**2**	ETH1_1; ETH3_1
**4**	***camelopardalis*_ETH1_2**	**2**	ETH1_2; ETH3_2
**5**	***peralta*_WA036_2**	**3**	WA036_2; WA619_2; WA708_2
**6**	***peralta*_WA117_2**	**15**	WA117_2; WA707_2; WA036_1; WA606_1; WA609_1; WA612_1; WA614_1; WA619_1; WA621_1; WA622_1; WA623_1; WA700_1; WA705_1; WA708_1; WA720_1
**7**	***reticulata*_RET3_2**	**18**	RET3_2; MF01_2; MF03_2; MF17_2; MF24_2; RETWil2_2; MF02_1; MF04_1; MF05_1; MF06_1; MF07_1; MF11_1; MF13_1; MF14_1; MF15_1; WA026_1; WA117_1; WA707_1
**8**	***reticulata*_RETWil_2**	**12**	RETWil_2; LWC01_2; GNP01_1; GNP03_1; GNP05_1; RET3_1; RETRot1_1; RETRot2_1; RETRot3_2; ISC04_1; SNR2_1; ZNP01_1
**9**	***reticulata*_RETRot3_1**	**2**	RETRot3_1; RET6_2
**10**	***antiquorum*_GNP01_1**	**11**	GNP01_2; GNP03_2; GNP05_2; SNR2_2; GNP04_1; MF01_1; MF03_1; MF09_1; MF17_1; MF24_1; RETWil2_1
**11**	***thornicrofti*_**LVNP31_1	**1**	LVNP31_1
**12**	***giraffa*_BNP01_1**	**85**	BNP01_1; BNP02_1; BNP03_1; BNP04_1; BNP05_1; BNP06_1; BNP09_1; CKGR01_1; CKGR02_1; CKGR03_1; CKGR05_1; CNP02_1; CNP03_1; ENP04_1; ENP07_1; ENP08_1; ENP09_1; ENP11_1; ENP12_1; ENP14_1; ENP15_1; ENP16_1; ENP17_1; ENP18_1; ENP19_1; ENP20_1; ENP21_1; ETH2_1; GNP02_1; KKR01_1; KKR02_1; KKR03_1; KKR04_1; KKR05_1; KKR07_1; KKR08_1; LVNP18_1; LVNP19_1; LVNP20_1; LVNP21_1; LVNP22_1; LVNP23a_1; LVNP32_1; LVNP33_1; LVNP34_1; LVNP35_1; LVNP36_1; MGR01_1; MGR02_1; MGR03_1; MGR04_1; MGR05_1; MTNP01_1; MTNP02_1; MTNP03_1; RET1_1; RETWil_1; ISC03_1; ISC08_1; LWC01_1; SGR01_1; SGR05_1; SGR06_1; SGR07_1; SGR12_1; SGR13_1; SGR14_1; SNNP_1; SUN1_1; SUN2_1; SUN3_1; SUN4_1; V23_1; V24_1; V25_1; V26_1; V27_1; V28_1; V29_1; V30_1; V31_1; V36_1; V37_1; V38_1; V39_1
**13**	***giraffa*_CNP01_1**	**1**	CNP01_1

Table Ah. Overview of the alleles comprised by the haplotypes shown in the haploweb of *OTOF*. Groups of individuals sharing an exclusive allele pool [field for recombination: FFR] are highlighted by a grey box.

n	Representative allele	∑	Allele sharing information
1	*camelopardalis*_BaNP4_1	26	BaNP4_1; ETH1_1; GNP01_1; GNP02_1; GNP03_1; GNP04_1; GNP05_1; MF01_1; MF02_1; MF03_1; MF05_1; MF06_1; MF09_1; MF11_1; MF13_1; MF14_1; MF15_1; MF16_1; MF17_2; MF24_1; RETWil2_1; SNR1_1; SNR2_1; WA036_1; WA606_1; WA708_1
2	*camelopardalis*_BaNP4_2	4	BaNP4_2; WA026_2; WA705_1; WA720_1
3	*antiquorum*_GNP01_2	20	GNP01_2; GNP03_2; MF02_2; MF03_2; MF06_2; MF09_2; MF11_2; MF15_2; RET4_2; RETRot2_2; ETH2_1; ETH3_1; MF04_1; MF07_1; RET1_1; RET3_2; RET6_2; RETRot1_2; RETRot3_1; ISC04_1
4	*peralta*_WA700_1	4	WA700_1; WA117_1; WA614_1; WA622_1
5	*peralta*_WA606_2	6	WA606_2; WA708_2; WA619_2; WA612_1; WA614_2; WA609_1
6	*peralta*_WA705_2	2	WA705_2; WA720_2
7	*peralta*_WA036_2	7	WA036_2; WA026_1; WA117_2; WA612_2; WA621_1; WA623_1; WA707_1
8	*reticulata*_RETWil2_2	8	RETWil2_2; RET5_2; RETWil_2; ISC08_2; RET3_1; RET6_1; RETRot1_1; ISC03_1
9	*tippelskirchi*_SGR01_2	5	SGR01_2; SGR07_2; LVNP31_1; SGR12_1; SGR13_1
10	*giraffa*_BNP01_1	57	BNP01_1; BNP02_1; BNP03_1; BNP04_1; BNP05_1; BNP06_1; BNP09_1; CKGR03_1; CKGR05_1; CNP01_1; CNP02_1; CNP03_1; ENP04_1; ENP07_1; ENP08_1; ENP09_1; ENP11_1; ENP12_1; ENP14_1; ENP15_1; ENP16_1; ENP17_1; ENP18_1; ENP19_1; ENP20_1; ENP21_1; KKR01_1; KKR02_1; KKR03_1; KKR04_1; KKR05_1; KKR07_1; KKR08_1; MGR01_1; MGR02_1; MGR03_1; MGR04_1; MGR05_1; MTNP02_1; SNNP_1; SUN1_1; SUN2_1; SUN3_1; SUN4_1; V23_1; V24_1; V25_1; V26_1; V27_1; V28_1; V29_1; V30_1; V31_1; V36_1; V37_1; V38_1; V39_1
11	*giraffa*_BNP02_2	43	BNP02_2; BNP04_2; CKGR05_2; CNP02_2; ENP04_2; ENP07_2; ENP08_2; ENP09_2; ENP20_2; KKR07_2; MGR04_2; V24_2; V25_2; V26_2; V29_2; V30_2; V31_2; CKGR01_1; CKGR02_1; LVNP18_1; LVNP19_1; LVNP20_1; LVNP21_1; LVNP22_1; LVNP23a_1; LVNP32_1; LVNP34_1; LVNP36_1; MTNP01_1; MTNP03_1; RET4_1; RET5_1; RETRot2_1; RETWil_1; ISC08_1; LWC01_1; SGR01_1; SGR05_1; SGR06_1; SGR07_1; SGR14_1; WA619_1; WA700_2
12	*thornicrofti*_LVNP35_1	1	LVNP35_1
13	*antiquorum*_ZNP01_1	1	ZNP01_1

Table Ai. Overview of the alleles comprised by the haplotypes shown in the haploweb of *PLCE1*. Groups of individuals sharing an exclusive allele pool [field for recombination: FFR] are highlighted by a grey box.

n	Representative allele	∑	Allele sharing information
1	***camelopardalis*_BaNP4_1**	**56**	BaNP4_1; CKGR01_1; ETH1_1; ETH2_1; ETH3_1; GNP01_1; GNP02_1; GNP03_1; GNP04_1; GNP05_1; MF01_1; MF02_1; MF03_1; MF04_1; MF05_1; MF07_1; MF11_1; MF13_1; MF14_1; MF15_1; MF16_1; MF17_1; MF24_1; RET1_1; RET3_1; RET4_1; RET5_1; RET6_1; RETRot1_1; RETRot2_1; RETRot3_1; RETWil_1; RETWil2_1; ISC03_1; ISC04_1; ISC08_1; SNR1_1; SNR2_1; V30_1; WA026_1; WA036_1; WA117_1; WA606_1; WA609_1; WA612_1; WA614_1; WA619_1; WA621_1; WA622_1; WA623_1; WA700_1; WA705_1; WA707_1; WA708_1; WA720_1; ZNP01_1
2	***rothschildi*_MF02_2**	**37**	MF02_2; MF04_2; MF07_2; MF15_2; MF17_2; BNP01_2; BNP02_2; BNP04_2; BNP09_2; BNP03_1; BNP05_2; BNP06_1; CNP03_1; ENP04_2; ENP07_2; ENP08_1; ENP09_2; ENP21_2; KKR01_1; KKR02_2; KKR03_1; KKR04_2; KKR07_2; KKR08_1; MF06_1; MF09_1; MGR01_1; MGR03_2; MGR04_1; MTNP01_2; MTNP02_2; MTNP03_2; V23_2; V24_2; V25_1; V36_2; V38_1
3	***reticulata*_RET5_2**	**7**	RET5_2; RET6_2; RETRot1_2; RETRot2_2; RETWil_2; RETWil2_2; LWC01_1
4	***angolensis*_ENP15_1**	**52**	ENP15_1; ENP18_1; ENP19_1; V37_1; BNP05_1; ENP04_1; ENP07_1; ENP09_1; ENP21_1; KKR02_1; KKR04_1; KKR07_1; MGR03_1; MTNP01_1; MTNP02_1; MTNP03_1; V23_1; CKGR02_1; CKGR03_1; CKGR05_1; ENP11_1; ENP12_1; ENP14_1; ENP17_1; ENP20_1; KKR05_1; LVNP18_1; LVNP19_1; LVNP20_1; LVNP21_1; LVNP22_1; LVNP23a_1; LVNP31_1; LVNP32_1; LVNP33_1; LVNP34_1; LVNP35_1; LVNP36_1; SGR01_1; SGR05_1; SGR06_1; SGR07_1; SGR12_1; SGR13_1; SGR14_1; SUN1_1; SUN2_1; SUN3_1; SUN4_1; V26_1; V28_1; V29_1
5	***giraffa*_BNP01_1**	**11**	BNP01_1; BNP02_1; BNP04_1; BNP09_1; CNP01_1; ENP15_2; ENP16_1; ENP18_2; ENP19_2; MGR05_2; V37_2
6	***giraffa*_V30_2**	**9**	V30_2; V24_1; V26_2; V28_2; MGR02_2; SNNP_1; V27_1; V31_1; V39_1

Table Aj. Overview of the alleles comprised by the haplotypes shown in the haploweb of *RASSF4*. Groups of individuals sharing an exclusive allele pool [field for recombination: FFR] are highlighted by a grey box.

n	Representative allele	∑	Allele sharing information
1	***camelopardalis*_ETH1_1**	**14**	ETH1_1; LVNP18_1; LVNP19_1; LVNP20_1; LVNP21_1; LVNP22_1; LVNP23a_1; SGR01_2; SGR05_2; SGR06_2; SGR07_1; SGR12_2; SGR13_1; SGR14_1
2	***reticulata*_RETRot1_2**	**6**	RETRot1_2; RETRot3_1; RETWil2_1; ISC03_1; ISC04_1; ISC08_1
3	***reticulata*_RETRot2_2**	**3**	RETRot2_2; RETRot3_2; RETWil2_2
4	***reticulata*_ISC04_2**	**2**	ISC04_2; ISC08_2
5	***reticulata*_RET4_1**	**2**	RET4_1; RETRot2_1
6	***thornicrofti*_LVNP23a_2**	**3**	LVNP23a_2; LVNP33_1; RET6_2

7	***tippelskirchi*_SGR01_1**	2	SGR01_1; SGR12_1
8	***camelopardalis*_ETH3_2**	3	ETH3_2; MF02_1; MF06_1
9	***rothschildi*_MF11_2**	2	MF11_2; MF17_1
10	***thornicrofti*_LVNP35_1**	1	LVNP35_1
11	***thornicrofti*_LVNP36_1**	1	LVNP36_1
12	***rothschildi*_MF04_2**	3	MF04_2; MF05_2; MF07_2
13	***rothschildi*_MF15_2**	2	MF15_2; MF24_2
14	***camelopardalis*_ETH2_1**	15	ETH2_1; MF01_1; MF03_1; MF04_1; MF05_1; MF07_1; MF09_1; MF13_1; MF14_1; MF15_1; MF16_1; MF24_1; WA619_1; WA621_1; WA622_1
15	***peralta*_WA622_2**	2	WA622_2; WA614_1
16	***peralta*_WA700_1**	1	WA700_1
17	***peralta*_WA720_1**	1	WA720_1
18	***antiquorum*_GNP01_1**	4	GNP01_1; GNP03_1; GNP04_1; SNR2_1
19	***giraffa*_BNP01_1**	61	BNP01_1; BNP02_1; BNP03_1; BNP04_1; BNP05_1; BNP06_1; BNP09_1; CKGR01_1; CKGR02_1; CKGR03_1; CKGR05_1; CNP01_1; CNP02_1; CNP03_1; ENP04_1; ENP07_1; ENP08_1; ENP09_1; ENP11_1; ENP12_1; ENP14_1; ENP15_1; ENP16_1; ENP17_1; ENP18_1; ENP19_1; ENP20_1; ENP21_1; GNP02_1; KKR01_1; KKR02_1; KKR03_1; KKR04_1; KKR05_1; KKR07_1; KKR08_1; MGR03_1; MGR04_1; MGR05_1; MTNP01_1; MTNP02_1; MTNP03_1; RET1_1; SNNP_1; SUN1_1; SUN2_1; SUN3_1; SUN4_1; V23_1; V24_1; V25_1; V26_1; V27_1; V28_1; V29_1; V30_1; V31_1; V36_1; V37_1; V38_1; V39_1
20	***angolensis*_CKGR02_2**	13	CKGR02_2; CNP01_2; GNP02_2; GNP05_1; SNR1_1; WA026_1; WA036_1; WA606_1; WA609_1; WA623_1; WA707_1; WA708_1; ZNP01_1
21	***thornicrofti*_LVNP31_1**	1	LVNP31_1
22	***thornicrofti*_LVNP34_1**	1	LVNP34_1
23	***tippelskirchi*_SGR06_1**	2	SGR06_1; RETRot1_1
24	***reticulata*_LWC01_1**	1	LWC01_1
25	***giraffa*_MGR01_1**	2	MGR01_1; MGR02_1
26	***giraffa*_MGR01_2**	2	MGR01_2; MGR02_2

Table Ak. Overview of the alleles comprised by the haplotypes shown in the haploweb of *RFC5*. Groups of individuals sharing an exclusive allele pool [field for recombination: FFR] are highlighted by a grey box.

n	Representative allele	∑	Allele sharing information
1	***camelopardalis*_BaNP4_1**	47	BaNP4_1; ETH1_1; ETH2_1; ETH3_1; GNP01_1; GNP03_1; GNP04_1; GNP05_1; MF01_1; MF02_1; MF03_1; MF04_1; MF05_1; MF06_1; MF07_1; MF09_1; MF11_1; MF13_1; MF14_1; MF15_1; MF16_1; MF17_1; MF24_1; RET1_1; RET4_1; RET6_1; RETWil2_1; ISC04_1; LWC01_1; SNR1_1; SNR2_1; WA026_1; WA036_1; WA117_1; WA606_1; WA609_1; WA612_1; WA614_1; WA619_1; WA621_1; WA622_1; WA623_1; WA700_1; WA705_1; WA707_1; WA720_1; ZNP01_1
2	***camelopardalis*_BaNP4_2**	2	BaNP4_2; ETH2_2
3	***antiquorum*_GNP05_2**	3	GNP05_2; ZNP01_2; GNP02_1
4	***peralta*_WA036_2**	10	WA036_2; WA117_2; WA609_2; WA612_2; WA621_2; WA622_2; WA700_2;

			WA705_2; WA707_2; WA708_1
5	*reticulata*_RET1_2	11	RET1_2; RET4_2; RET6_2; RETWil2_2; ISC04_2; RET3_2; RET5_1; RETRot1_1; RETWil_2; ISC03_1; ISC08_2
6	*reticulata*_RET3_1	3	RET3_1; RETWil_1; ISC08_1
7	*giraffa*_KKR05_1	1	KKR05_1
8	*giraffa*_BNP01_1	53	BNP01_1; BNP02_1; BNP03_1; BNP04_1; BNP05_1; BNP06_1; BNP09_1; CKGR01_1; CKGR02_1; CKGR03_1; CKGR05_1; CNP01_1; CNP02_1; CNP03_1; ENP04_2; ENP07_1; ENP09_1; ENP14_2; ENP16_1; ENP18_2; ENP19_1; KKR01_1; KKR02_1; KKR03_1; KKR04_1; KKR07_1; KKR08_1; MGR01_1; MGR02_1; MGR03_1; MGR04_1; MGR05_1; MTNP01_1; MTNP02_1; MTNP03_1; SNNP_1; SUN1_1; SUN2_1; SUN3_1; SUN4_1; V23_1; V24_1; V25_1; V26_1; V27_1; V28_1; V29_1; V30_1; V31_1; V36_1; V37_1; V38_1; V39_1
9	*angolensis*_ENP08_1	2	ENP08_1; ENP12_1
10	*angolensis*_ENP12_2	2	ENP12_2; ENP15_2
11	*angolensis*_ENP14_1	6	ENP14_1; ENP08_2; ENP11_1; ENP15_1; ENP20_1; ENP21_1
12	*angolensis*_ENP18_1	2	ENP18_1; ENP17_1
13	*thornicrofti*_LVNP18_1	19	LVNP18_1; LVNP19_1; LVNP20_2; LVNP21_1; LVNP22_1; LVNP23a_1; LVNP31_1; LVNP32_1; LVNP33_2; LVNP34_1; LVNP35_1; LVNP36_1; SGR01_1; SGR05_1; SGR06_1; SGR07_1; SGR12_1; SGR13_1; SGR14_1
14	*thornicrofti*_LVNP20_1	2	LVNP20_1; LVNP33_1

Table AI. Overview of the alleles comprised by the haplotypes shown in the haploweb of *SAP130*. Groups of individuals sharing an exclusive allele pool [field for recombination: FFR] are highlighted by a grey box.

n	Representative allele	∑	Allele sharing information
1	*camelopardalis*_BaNP4_1	47	BaNP4_1; ETH1_1; ETH2_1; ETH3_1; GNP01_1; GNP02_1; GNP03_1; GNP04_1; GNP05_1; MF01_1; MF02_1; MF03_1; MF04_1; MF05_1; MF06_1; MF07_1; MF09_1; MF11_1; MF13_1; MF14_1; MF15_1; MF16_1; MF17_1; MF24_1; RET1_1; RET3_1; RET4_1; RETWil2_1; ISC03_1; LWC01_1; SNR1_1; WA026_1; WA036_1; WA117_1; WA606_1; WA609_1; WA612_1; WA614_1; WA619_1; WA622_1; WA623_1; WA700_1; WA705_1; WA707_1; WA708_1; WA720_1; ZNP01_1
2	*peralta*_WA036_2	3	WA036_2; WA612_2; WA622_2
3	*peralta*_WA606_2	2	WA606_2; WA621_1
4	*reticulata*_RET1_2	8	RET1_2; RET6_2; RETRot1_2; RETRot2_1; RETRot3_2; RETWil_1; ISC04_1; ISC08_1
5	*reticulata*_LWC01_2	5	LWC01_2; RET6_1; RETRot1_1; RETRot3_1; RET5_2
6	*antiquorum*_SNR2_1	1	SNR2_1
7	*tippelskirchi*_SGR05_1	15	SGR05_1; SGR06_1; SGR14_1; LVNP18_1; LVNP19_1; LVNP20_1; LVNP21_1; LVNP22_1; LVNP23a_1; LVNP31_1; LVNP32_1; LVNP33_1; LVNP34_1; LVNP35_1; LVNP36_1
8	*giraffa*_BNP01_1	67	BNP01_1; BNP02_1; BNP03_1; BNP04_1; BNP05_1; BNP06_1; BNP09_1; CKGR01_1; CKGR02_1; CKGR05_1; CNP01_1; CNP02_1; CNP03_1; ENP04_1; ENP07_1; ENP08_1; ENP09_1; ENP11_1; ENP12_1; ENP14_1; ENP15_1; ENP16_1; ENP17_1; ENP18_1; ENP19_1; ENP20_1; ENP21_1; KKR01_1; KKR02_1; KKR03_1; KKR04_1; KKR05_1; KKR07_1; KKR08_1; MGR01_1;

			MGR02_1; MGR03_1; MGR04_1; MGR05_1; MTNP01_1; MTNP02_1; MTNP03_1; SGR01_1; SGR05_2; SGR06_2; SGR07_1; SGR12_1; SGR13_1; SGR14_2; SNNP_1; SUN1_1; SUN2_1; SUN3_1; SUN4_1; V23_1; V24_1; V25_1; V26_1; V27_1; V28_1; V29_1; V30_1; V31_1; V36_1; V37_1; V38_1; V39_1
**9**	***angolensis*_CKGR03_1**	**1**	CKGR03_1

Table Am. Overview of the alleles comprised by the haplotypes shown in the haploweb of *SOS1*. Groups of individuals sharing an exclusive allele pool [field for recombination: FFR] are highlighted by a grey box.

n	Representative allele	∑	Allele sharing information
**1**	***camelopardalis*_BaNP4_1**	**49**	BaNP4_1; ETH1_1; ETH2_1; ETH3_1; GNP01_1; GNP02_1; GNP03_1; GNP04_1; GNP05_1; MF01_1; MF02_1; MF03_1; MF04_1; MF05_1; MF06_1; MF07_1; MF09_1; MF11_1; MF13_1; MF15_1; MF16_1; MF17_1; MF24_1; RET1_1; RET3_1; RET4_1; RET5_1; RET6_1; RETRot1_1; RETRot2_1; RETRot3_1; RETWil_1; RETWil2_1; ISC04_1; ISC08_1; LWC01_1; SNR1_1; SNR2_1; WA026_1; WA036_1; WA621_1; WA622_1; WA623_1; WA700_1; WA705_1; WA707_1; WA708_1; WA720_1; ZNP01_1
**2**	***rothschildi*_MF05_2**	**5**	MF05_2; MF07_2; ISC04_2; ISC08_2; LWC01_2
**3**	***reticulata*_RET5_2**	**4**	RET5_2; RET6_2; RETRot1_2; RETWil_2
**4**	***rothschildi*_MF14_1**	**1**	MF14_1
**5**	***reticulata*_ISC03_1**	**1**	ISC03_1
**6**	***thornicrofti*_LVNP32_1**	**1**	LVNP32_1
**7**	***giraffa*_BNP01_1**	**20**	BNP01_1; BNP03_1; BNP04_1; BNP05_1; BNP06_1; BNP09_1; CKGR05_1; KKR01_1; KKR03_1; MGR01_1; MGR02_1; MGR04_1; MTNP01_1; MTNP02_1; MTNP03_1; V29_1; V31_1; V36_1; V37_1; V38_1
**8**	***giraffa*_BNP03_2**	**51**	BNP03_2; BNP05_2; BNP06_2; MGR02_2; MTNP02_2; V29_2; V31_2; V36_2; V37_2; V38_2; BNP02_1; CKGR01_1; CKGR02_1; CKGR03_1; CNP01_1; CNP02_1; CNP03_1; ENP04_1; ENP07_1; ENP08_1; ENP09_1; ENP11_1; ENP12_1; ENP14_1; ENP15_1; ENP16_1; ENP17_1; ENP18_1; ENP19_1; ENP20_1; ENP21_1; KKR02_1; KKR04_1; KKR05_1; KKR07_1; KKR08_1; MGR03_2; MGR05_1; SNNP_1; SUN1_1; SUN2_1; SUN3_1; SUN4_1; V23_1; V24_1; V25_1; V26_1; V27_1; V28_1; V30_1; V39_2
**9**	***giraffa*_KKR03_2**	**20**	KKR03_2; MGR03_1; LVNP18_1; LVNP19_1; LVNP20_1; LVNP21_1; LVNP22_1; LVNP23a_1; LVNP31_1; LVNP33_1; LVNP34_1; LVNP35_1; LVNP36_1; SGR01_1; SGR05_1; SGR06_1; SGR07_1; SGR12_1; SGR13_1; SGR14_1
**10**	***angolensis*_CKGR01_2**	**5**	CKGR01_2; CNP03_2; ENP16_2; V26_2; V39_1

Table An. Overview of the alleles comprised by the haplotypes shown in the haploweb of *UBN2*. Groups of individuals sharing an exclusive allele pool [field for recombination: FFR] are highlighted by a grey box.

n	Representative allele	∑	Allele sharing information
1	***camelopardalis*_BaNP4_1**	25	BaNP4_1; ETH1_2; ETH2_1; ETH3_1; GNP01_1; GNP02_2; GNP04_1; GNP05_2; MF03_2; MF11_1; MF15_1; MF16_1; MTNP03_1; RET1_1; RET3_2; RET5_1; RET6_1; RETRot3_1; SNNP_2; SNR1_1; SNR2_1; WA036_1; WA612_2; WA614_2; ZNP01_1
2	***camelopardalis*_ETH1_1**	35	ETH1_1; ETH2_2; ETH3_2; GNP02_1; GNP05_1; MF03_1; MF15_2; RET3_1; RETRot3_2; SNNP_1; WA612_1; WA614_1; GNP03_1; MF01_2; MF02_1; MF04_1; MF05_2; MF06_1; MF07_2; MF09_1; MF13_1; MF14_1; MF17_1; MF24_1; RETWil_1; WA026_1; WA117_1; WA606_1; WA609_1; WA619_1; WA621_1; WA622_1; WA623_1; WA700_1; WA705_1
3	***rothschildi*_MF01_1**	3	MF01_1; MF05_1; MF07_1
4	***reticulata*_RET4_1**	8	RET4_1; RETRot1_1; RETRot2_1; RETWil2_1; ISC03_1; ISC04_1; ISC08_1; LWC01_1
5	***peralta*_WA707_1**	1	WA707_1
6	***peralta*_WA720_1**	1	WA720_1
7	***giraffa*_BNP01_1**	43	BNP01_1; BNP03_1; BNP04_1; BNP06_1; BNP09_1; CKGR01_1; CKGR02_1; CKGR03_1; CKGR05_1; CNP03_1; ENP04_1; ENP07_1; ENP08_1; ENP09_1; ENP11_1; ENP12_1; ENP14_1; ENP15_1; ENP16_1; ENP17_1; ENP18_1; ENP19_1; ENP20_1; ENP21_1; KKR01_1; KKR02_1; KKR03_1; KKR04_1; KKR05_1; KKR07_1; KKR08_1; MGR01_1; MGR03_1; MTNP01_1; MTNP02_1; SUN1_1; SUN3_1; SUN4_1; V24_1; V25_1; V28_1; V36_1; V37_1
8	***giraffa*_BNP03_2**	30	BNP03_2; BNP06_2; CKGR03_2; ENP04_2; ENP08_2; ENP11_2; ENP14_2; ENP20_2; MGR03_2; SUN1_2; SUN4_2; V24_2; V36_2; V37_2; BNP02_2; BNP05_1; CNP01_1; CNP02_1; MGR02_1; MGR04_1; MGR05_1; SUN2_1; V23_1; V26_1; V27_1; V29_1; V30_1; V31_1; V38_1; V39_1
9	***thornicrofti*_LVNP18_1**	19	LVNP18_1; LVNP19_1; LVNP20_1; LVNP21_1; LVNP22_1; LVNP23a_1; LVNP31_1; LVNP32_1; LVNP33_1; LVNP34_1; LVNP35_1; LVNP36_1; SGR01_1; SGR05_1; SGR06_1; SGR07_1; SGR12_1; SGR13_1; SGR14_2

Table Ao. Overview of the alleles comprised by the haplotypes shown in the haploweb of *USP33*. Groups of individuals sharing an exclusive allele pool [field for recombination: FFR] are highlighted by a grey box.

n	Representative allele	∑	Allele sharing information
1	***camelopardalis*_ETH1_1**	29	ETH1_1; ETH2_1; ETH3_1; GNP02_1; GNP04_1; MF02_1; MF03_1; MF04_1; MF05_1; MF06_1; MF11_1; MF13_1; MF14_1; MF16_1; MF17_1; MF24_1; RET4_1; RET5_1; RET6_1; RETWil2_1; ISC04_1; LWC01_1; SNR1_1; SNR2_1; WA036_1; WA606_1; WA612_1; WA621_1; ZNP01_1
2	***camelopardalis*_ETH1_2**	20	ETH1_2; ETH2_2; GNP02_2; GNP04_2; MF06_2; MF11_2; MF13_2; MF16_2; WA612_2; GNP01_1; GNP03_1; GNP05_1; RET3_2; RETRot1_2; WA026_2; WA117_2; WA622_2; WA623_2; WA705_2; WA707_1

3	*camelopardalis*_ETH3_2	13	ETH3_2; RETWil2_2; WA036_2; RET3_1; RETRot1_1; WA622_1; WA623_1; MF01_1; RETRot2_1; RETWil_1; ISC03_1; ISC08_1; WA614_1
4	*rothschildi*_MF03_2	8	MF03_2; MF05_2; MF14_2; MF17_2; MF01_2; MF07_1; MF09_1; MF15_1
5	*reticulata*_RET4_2	4	RET4_2; RET6_2; RETWil_2; ISC03_2
6	*reticulata*_ISC04_2	5	ISC04_2; LWC01_2; RETRot2_2; RET1_1; RETRot3_2
7	*peralta*_WA117_1	6	WA117_1; WA705_1; WA619_1; WA700_1; WA708_1; WA720_1
8	*peralta*_WA614_2	3	WA614_2; WA708_2; WA720_2
9	*peralta*_WA621_2	4	WA621_2; WA619_2; WA700_2; WA609_1
10	*thornicrofti*_LVNP18_1	19	LVNP18_1; LVNP19_1; LVNP20_1; LVNP21_1; LVNP22_1; LVNP23a_1; LVNP31_1; LVNP32_1; LVNP33_1; LVNP34_1; LVNP35_1; LVNP36_1; SGR01_1; SGR05_1; SGR06_1; SGR07_1; SGR12_1; SGR13_1; SGR14_1
11	*giraffa*_BNP01_1	56	BNP01_1; BNP02_1; BNP03_1; BNP04_1; BNP05_1; BNP06_1; BNP09_1; CKGR03_1; CKGR05_1; CNP01_1; CNP02_1; CNP03_1; ENP04_1; ENP07_1; ENP09_1; ENP11_1; ENP12_1; ENP14_1; ENP17_1; ENP18_1; ENP19_1; ENP20_1; ENP21_1; KKR01_1; KKR02_1; KKR03_1; KKR04_1; KKR05_1; KKR07_1; KKR08_1; MGR01_1; MGR02_1; MGR03_1; MGR04_1; MGR05_1; MTNP01_1; MTNP02_1; MTNP03_1; SNNP_1; SUN1_1; SUN2_1; SUN3_1; SUN4_1; V23_1; V24_1; V25_1; V26_1; V27_1; V28_1; V29_1; V30_1; V31_1; V36_1; V37_1; V38_1; V39_1
12	*angolensis*_CKGR05_2	13	CKGR05_2; CNP03_2; ENP07_2; ENP09_2; ENP14_2; ENP17_2; ENP19_2; V29_2; CKGR01_1; CKGR02_1; ENP08_1; ENP15_1; ENP16_1

Table Ap. Overview of the alleles comprised by the haplotypes shown in the haploweb of *USP54*. Groups of individuals sharing an exclusive allele pool [field for recombination: FFR] are highlighted by a grey box.

n	Representative allele	∑	Allele sharing information
1	*camelopardalis*_BaNP4_1	26	BaNP4_1; ETH1_1; ETH2_1; GNP05_1; MF01_1; MF02_1; MF03_1; MF05_1; MF06_1; MF07_1; MF09_1; MF11_1; MF13_1; MF14_1; MF15_1; MF16_1; MF17_1; MF24_1; RETRot2_1; SNR1_1; SNR2_1; WA026_1; WA036_1; WA606_1; WA700_1; WA720_1
2	*camelopardalis*_BaNP4_2	25	BaNP4_2; ETH1_2; ETH2_2; GNP05_2; MF01_2; MF06_2; MF07_2; MF11_2; MF13_2; MF14_2; SNR1_2; SNR2_2; GNP01_1; GNP02_1; GNP03_1; GNP04_1; MF04_1; WA612_1; WA614_1; WA621_1; WA622_1; WA623_1; WA705_1; WA708_1; ZNP01_2
3	*peralta*_WA036_2	14	WA036_2; WA700_2; WA720_2; WA612_2; WA614_2; WA621_2; WA622_2; WA623_2; WA705_2; LWC01_1; WA117_1; WA609_1; WA619_1; WA707_1
4	*reticulata*_RET1_1	8	RET1_1; RET4_1; RET5_1; RET6_1; RETRot1_1; RETRot3_1; RETWil_1; RETWil2_1
5	*reticulata*_ISC03_1	3	ISC03_1; ISC04_1; ISC08_1
6	*giraffa*_BNP01_1	38	BNP01_1; BNP02_1; BNP03_1; BNP04_1; BNP05_1; BNP06_1; BNP09_1; CKGR01_1; CKGR02_1; CKGR03_1; CKGR05_2; CNP01_2; CNP02_2; CNP03_2; ENP09_2; ENP12_1; ENP14_1; ENP15_2; ENP20_2; ENP21_2; KKR02_1; KKR03_2; KKR05_2; KKR07_2; KKR08_1; MGR04_2; MTNP02_1; SNNP_1; V23_2; V24_1; V25_1; V26_2; V27_2; V28_2; V31_2; V36_1; V37_2; V39_2

**7**	***angolensis*_CKGR05_1**	**44**	CKGR05_1; CNP01_1; CNP02_1; CNP03_1; ENP09_1; ENP15_1; ENP20_1; ENP21_1; KKR03_1; KKR05_1; KKR07_1; MGR04_1; V23_1; V26_1; V27_1; V28_1; V31_1; V37_1; V39_1; ENP04_1; ENP07_1; ENP08_1; ENP11_1; ENP16_1; ENP17_1; ENP18_1; ENP19_1; KKR01_1; KKR04_1; LVNP31_1; LVNP34_1; MGR01_1; MGR02_1; MGR03_1; MGR05_1; MTNP01_1; MTNP03_1; SUN1_1; SUN2_1; SUN3_1; SUN4_1; V29_1; V30_1; V38_1
**8**	***tippelskirchi*_SGR07_1**	**2**	SGR07_2; SGR12_1
**9**	***thornicrofti*_LVNP21_1**	**13**	LVNP21_1; LVNP23a_1; LVNP32_1; LVNP36_1; LVNP19_1; LVNP22_1; LVNP33_1; LVNP35_1; SGR01_1; SGR06_1; SGR07_1; SGR13_1; SGR14_1
**10**	***thornicrofti*_LVNP31_2**	**7**	LVNP31_2; LVNP18_1; LVNP20_1; LVNP21_2; LVNP23a_2; LVNP32_2; LVNP36_2
**11**	***thornicrofti*_LVNP34_2**	**2**	LVNP34_2; SGR05_1
